# Ultra‐Radiostable Covalent Conformationally Interlocked Networks Enabling a Universal Radiometal‐Labeling Platform for Cancer Radioembolization

**DOI:** 10.1002/advs.76278

**Published:** 2026-06-23

**Authors:** Xiao Xu, Zhenwen Zhao, Yangjie Wang, Zhigang Liu, Zhijie Liu, Xiaoling Li, Zhichen Mao, Lu Xu, Gan Lin, Gang Liu, Hongjuan Ma

**Affiliations:** ^1^ Cancer Center Guangdong Engineering Research Center of Boron Neutron Therapy and Application in Malignant Tumors Dongguan Key Laboratory of Precision Diagnosis and Treatment for Tumors The Tenth Affiliated Hospital, Southern Medical University (Dongguan People’s Hospital) Dongguan China; ^2^ State Key Laboratory of Vaccines for Infectious Diseases Xiang An Biomedicine Laboratory National Innovation Platform for Industry‐Education Integration in Vaccine Research School of Public Health Xiamen University Xiamen China; ^3^ Shanghai Advanced Research Institute Chinese Academy of Sciences Shanghai China; ^4^ Shanghai Applied Radiation Institute School of Environmental and Chemical Engineering Shanghai University Shanghai China; ^5^ Shanghai Institute of Applied Physics Chinese Academy of Sciences Shanghai China

**Keywords:** covalent conformational interlocked networks, radionuclides, theranostics, ultra‐radiostability

## Abstract

Transcatheter arterial embolization (TARE) has emerged as a highly effective locoregional treatment for advanced liver cancer, which represents a significant advancement in clinical practice. Nonetheless, the utilization of traditional radioactive microspheres in TARE is hindered by various material and technical challenges that necessitate resolution. Herein, we present, for the first time, well‐designed and synthesized conjugated poly(imide dioxime) ligand‐based microspheres (PID‐Ms) with a remarkable radiometal coordinate covalent conformational interlocked network. PID‐Ms can be radiolabeled with various radionuclides, including ^177^Lu, ^90^Y, ^188^Re, ^68^Ga, and ^99m^Tc, can be achieved at low temperatures (40°C), and for diagnostic imaging and radiotherapy applications. The combined strategy of extended x‐ray absorption fine structure spectroscopy (EXAFS), DFT calculations, and in vitro and in vivo radiostability experiments collectively confirmed that radiometal‐PID complexes exhibit ultra‐high radiostability. ^177^Lu‐PID‐Ms were employed to accurately predict normal organ shunts and radiotherapy in rat and rabbit VX2 orthotopic live tumor models, demonstrating their ultra‐radiostability and remarkable anti‐tumor efficacy. Furthermore, Radiometals‐PID‐Ms have an adjustable shelf life, enabling on‐demand radiolabeling for drug preparation, achieving rapid ‘on‐demand’ capability. This innovative approach has the potential to inspire the synthesis of numerous coordination polymeric materials for various applications in PET/SPECT‐mediated radiopharmaceutical therapy, alpha‐nucleophile‐targeted radiotherapy, and MRI contrast agents.

## Graphical abstract

Conjugated poly(imide dioxime)‐based microspheres establish a radiometal coordination‐driven conformational interlocked network with ultra‐high radiostability. This platform enables low‐temperature, multi‐radionuclide labeling for SPECT/PET/MRI imaging and radionuclide therapy. Mechanistic insights from EXAFS and DFT reveal enhanced stability, while in vivo studies demonstrate effective tumor targeting, supporting clinical translation for liver cancer theranostics.

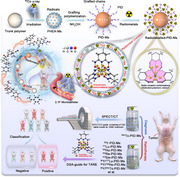

## 1 Introduction

Cancer remains one of the leading causes of death worldwide, with nearly 10 million deaths and nearly 20 million new cases of cancer each year [1]. As an important antitumor treatment, radiotherapy is received by more than 50% of cancer patients for curative and palliative purposes [2]. Radiotherapy comprises external‐beam techniques and internal approaches; internal radiotherapy delivers ionizing radiation via radioactive sources placed adjacent to or within the tumor (brachytherapy) or administered systemically as radiopharmaceuticals [3]. The radioactive material is intended for selective accumulation in cancer cells or the vascular beds of vascular‐rich solid tumors and can be used for therapy or imaging, depending on the radionuclide. *γ^−^* and *β^+^* emitting radionuclides (e.g., ^99m^Tc, ^111^In, ^68^Ga, or ^64^Cu) are used for single‐photon emission computed tomography (SPECT) or positron emission tomography (PET) imaging, respectively [4, 5, 6]. *β‐* or *α*‐emitting radionuclides (e.g., ^177^Lu, ^90^Y, ^188^Re, ^166^Ho, ^223^Ra, and ^225^Ac) directly or indirectly disrupt the tumor cell's DNA double‐strand structure for effective radiotherapy. Radioactive materials generally consist of four components: a radioisotope, a bifunctional chelate, a linker, and a targeting vector (e.g., an antibody or peptide) [7, 8]. Covalent bond formation (e.g., ^18^F‐fluorodeoxyglucose) is one way to achieve radiolabeling, and a wider variety of radiopharmaceuticals can be obtained through the use of radioactive metals and chelators. Thus, developing ligands that form strong coordination bonds with radiometals is essential. Bifunctional chelators are key to radiopharmaceutical performance. The 1,4,7,10‐tetraazacyclododecane‐1,4,7,10‐tetraacetic acid (DOTA) is the most widely used chelator in nuclear medicine, yet it often underperforms because it does not adequately account for the fundamental properties of radiometal ions (e.g., atomic number, charge, radius) [9, 10]. Macrocyclic chelators (e.g., DOTA, 1,4,7‐triazacyclononane‐1,4,7‐triacetic acid (NOTA)) generally offer higher kinetic inertness than acyclic ones due to their rigid structures [11], but they suffer from slow binding kinetics, require high temperatures and long reaction times, and are incompatible with heat‐sensitive biomolecules or short‐lived isotopes [12, 13]. Acyclic chelators such as diethylenetriaminepentaacetic acid (DTPA) allow rapid room‐temperature labeling but tend to be less stable in vivo [14, 15]. To address this issue, researchers have developed a large number of bifunctional chelators such as macropa [16], py‐macrodipa [17, 18], H_4_noneunpa [19], H4py4pa [20], H4octapa [21, 22], and H4octox [23], and some radiotherapy results are promising.

The precise delivery of large doses of radionuclides into the tumor is one essential factor for the success of radiotherapy. Unlike systemic radionuclide therapy, brachytherapy delivers therapeutic doses of radionuclides through direct intratumoral injection for the treatment of unresectable solid tumors [24, 25]. Compared to systemic radionuclide therapy, interventional radionuclide therapy, such local delivery enables higher doses of radiopharmaceuticals to solid tumors while reducing non‐specific radiation exposure to normal organs. Furthermore, the radioactive material for transcatheter arterial radiotherapy embolization (TARE) of vascular‐rich solid tumors consists only of radionuclide and macromolecular chelator substrate material, benefiting from interventional precision delivery techniques without the need for linkers and targeting carriers. Recently, radioactive materials for TARE have emerged as a promising radiopharmaceutical for cancer radiotherapy, such as ^90^Y, ^166^Ho, or ^188^Re microspheres [26, 27, 28, 29]. However, the utilization of traditional radioactive microspheres is hindered by a range of material and technical challenges that necessitate resolution. These challenges encompass issues like inadequate tumor‐to‐normal liver uptake (T/N), dependency on high‐flux neutron reactors, intricate manufacturing procedures, restricted capability for radionuclide labeling, short shelf life, and a scarcity of mechanistic studies.

**FIGURE 1:**
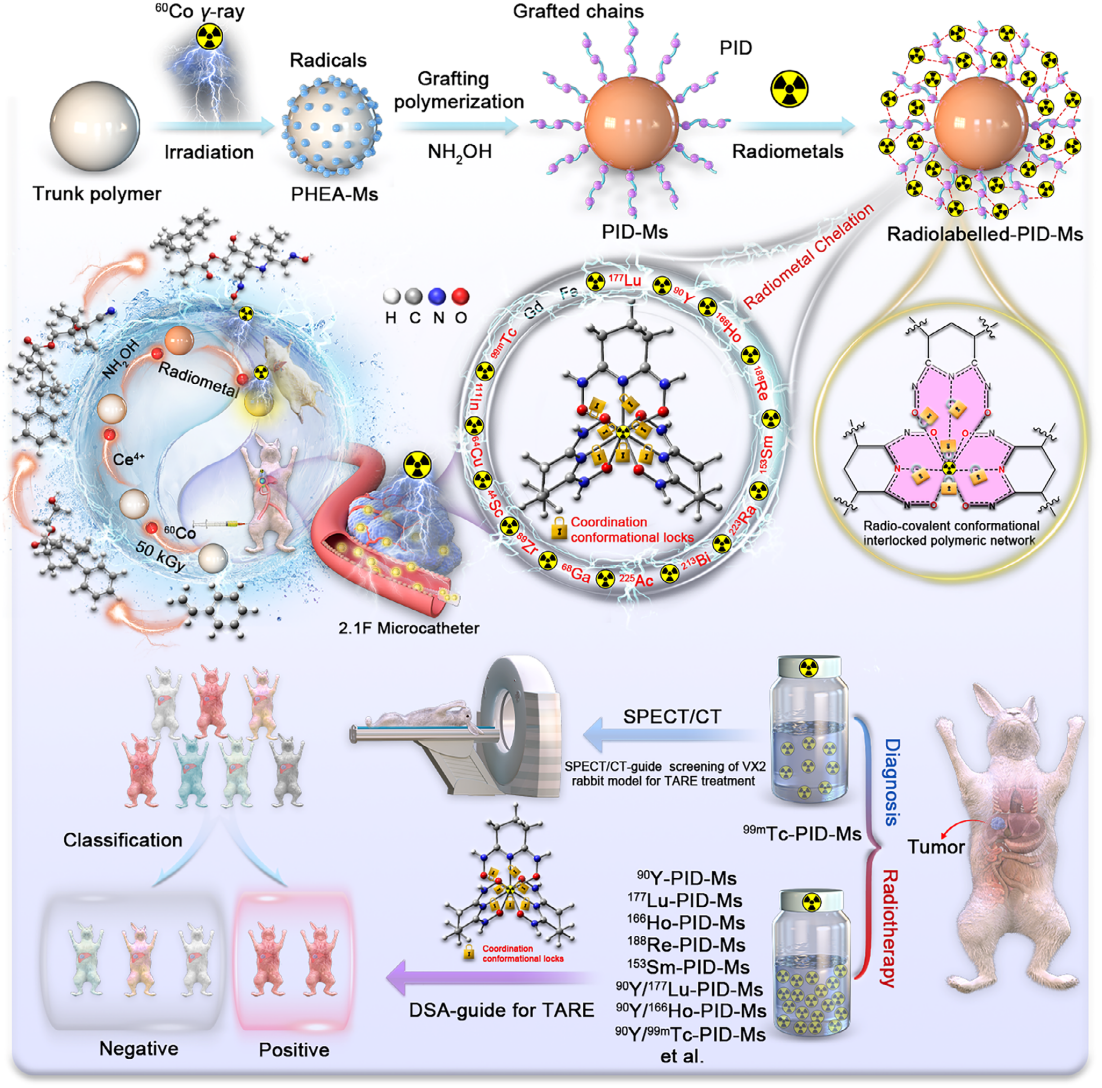
Schematic diagram depicting the synthetic strategy to achieve ultra‐radiostability conjugated complexes via constructing radio‐covalent conformational interlocked polymeric networks. Our synthetic PID‐Ms have a wide range of radiometal labeling universality and can be used in biological imaging (SPECT, PET, MRI, etc.) and in vivo radionuclide therapy. The chelating coordination configuration of PID ligand and radionuclide as well as the mechanism of in vitro kinetics and thermodynamic inertness were revealed through radioactive (“hot”) and nonradioactive (“cold”) metal ion labeling experiments combined with DFT theoretical calculation. The diagnostic integration radionuclides such as ^177^Lu and ^90^Y were labeled at low temperatures for the diagnosis and treatment of orthotopic live tumor models in rats and rabbits, showing significant antitumor efficacy. It meets the clinical demand of interventional internal radiation therapy, provides the theoretical and experimental basis for clinical transformation, and has a far‐reaching influence in the interdisciplinary field of science, engineering, and medicine. Schematic diagram of the clinical translation of engineering Radiometals‐PID‐Ms for liver cancer theranostics. A novel multi‐functional radioactive conjugated polymeric microspheres library has been constructed, and a radiometal coordination covalent conformational interlocked network based on radiometal coordination atom conformational lock.

Here, we first report a novel conjugated poly(imide dioxime) (PID) ligand functions as a radiometal chelator, and as a radioactive microspheres embolization agent with radio‐covalent conformational interlocked networks. This conjugated PID ligand–based chelator microspheres (PID‐Ms) could efficiently label one or more radioisotopes, including ^177^Lu (^177^Lu‐PID‐Ms), ^90^Y (^90^Y‐PID‐Ms), ^188^Re (^188^Re‐PID‐Ms), ^68^Ga (^68^Ga‐PID‐Ms), and ^99m^Tc (^99m^Tc‐PID‐Ms), under mild conditions (pH 6–7, 40°C), which benefits from the high flexibility of bridged organic ligands in PID. In this radiometal‐PID‐Ms complex material, radiometal ions form covalent bonds with the imide group of PID ligand and the coordination bonds with the oxime group, transforming the two‐dimensional symmetric structure of PID into a unique 3D tridentate symmetric conformation (Figure 1a). The PID molecular chains construct the ultra‐radiostability radiometal‐mediated covalent conformational interlocked network. As a result, the planar structure of PID molecular chains converts to a 3D tridentate chelated structure, in which three imide dioxime chains are bridged by radiometal to form a radio‐covalent conformational interlocked network, oriented along the PID chain direction. The radiometal‐PID‐Ms complex exhibits excellent labeling kinetic and thermodynamic inertness in vivo, which enables it to be used as a radio‐complexes with clinical application. These characteristics enable radiometal‐PID‐Ms to act as an effective radiopharmaceutical for antitumor, as demonstrated in different preclinical rodent orthotopic live tumor models (Figure 1a).

## 2 Results and Discussion

### 2.1 Synthesis and Characterization of the PID‐Ms

The synthesis routes and synthetic mechanism of PID‐Ms and radiometal‐PID‐Ms (^177^Lu‐PID‐Ms, ^90^Y‐PID‐Ms, ^188^Re‐PID‐Ms, ^68^Ga‐PID‐Ms, and ^99m^Tc‐PID‐Ms) are described in the Methods and Figure S1. In brief, the PID‐Ms were fabricated by grafted polymerization involving the emulsion suspension polymerization for obtained polystyrene microspheres (PS‐Ms) and the ^60^Co γ‐rays (adsorbed dose: 50 kGy) radiation‐induced graft polymerization of 2‐hydroxyethyl acrylate (HEA) onto a PS‐Ms followed by the Ce^4+^‐induced graft polymerization of acrylonitrile (AN) onto the poly‐HEA (PHEA‐Ms) chains to obtained PAN‐Ms, and then obtained by reaction with hydroxylamine in DMSO for three days. Subsequently, the PID‐Ms were labeled directly with ^177^LuCl_3_, ^90^YCl_3_, Na^188^ReO4 (SnCl_2_ reduction), ^68^GaCl_3_, Na^99m^TcO_4_ (SnCl_2_ reduction) to obtained the radio‐PID‐Ms (^177^Lu‐PID‐Ms, ^90^Y‐PID‐Ms, ^188^Re‐PID‐Ms, ^223^Ra‐PID‐Ms, ^68^Ga‐PID‐Ms, and ^99m^Tc‐PID‐Ms). Labeling method are described in the Methods and Supporting Information.

**FIGURE 2:**
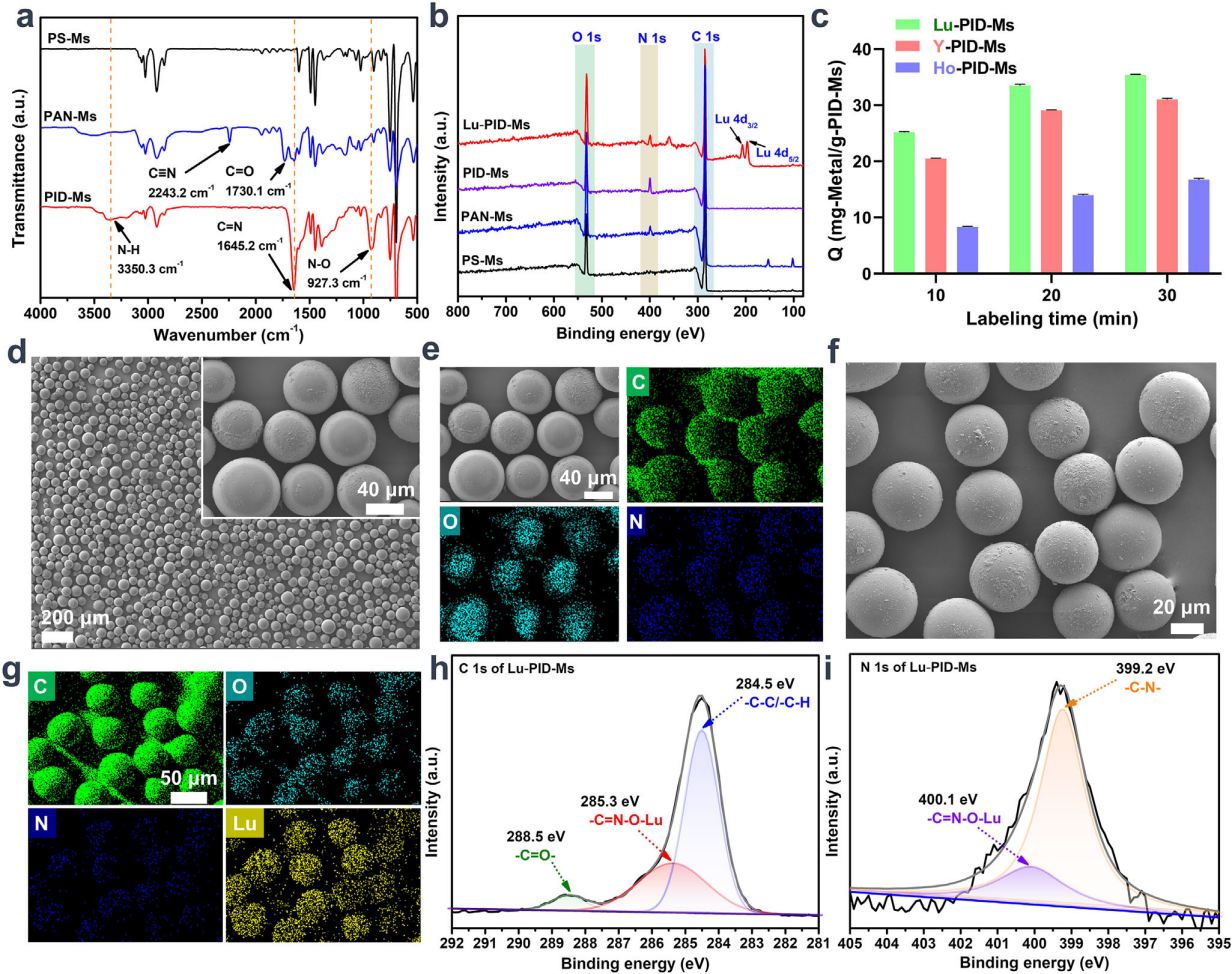
Physiochemical characterization of PID‐Ms. (a) FT‐IR characterization of PS‐Ms, PAN‐Ms, and PID‐Ms. (b) XPS spectrum of PS‐Ms, PAN‐Ms, PID‐Ms, and Lu‐PID‐Ms, respectively. (c) Complexation kinetics of PID‐Ms in an initial Lu^3+^, Y^3+^, and Ho^3+^ concentration of 10 ppm. (d) SEM images of PID‐Ms at different magnifications. (e) EDS mapping of PID‐Ms. (f) SEM images of Lu‐PID‐Ms. (g) EDS mapping of Lu‐PID‐Ms. High‐resolution XPS of (h) C 1s and (i) N 1s for Lu‐PID‐Ms, respectively.

Fourier transform infrared spectrometry (FT‐IR) and x‐ray photoelectron spectroscopy (XPS) was employed to characterize the chemical structures of the obtained materials. The FT‐IR of functional group‐modified materials are shown in Figure 2a. Compared with the PS‐Ms spectra, the new stretching vibrations of C═O occurred at 1,730.1 cm^−1^, indicating that HEA were all successfully grafted onto the PS‐Ms. The disappearance of the nitrile stretch peak (C≡N) at 2,243.2 cm^−1^ and the emergence of peaks at 927.3 cm^−1^ (N─O), 1645.2 cm^−1^(C═N), and 3350.3 cm^−1^ (N─H), which were related to the stretching vibrations of the conjugated PID ligand, confirmed the successful synthesis of PID‐Ms. On the XPS spectra, compared with the PS‐Ms, PAN‐Ms showed a new peak at 398.6 eV (N 1s) indicating the successful grafting of PAN onto the PHEA‐Ms. Following the reaction with hydroxylamine and heat treatment with DMSO, the intensity of N 1S in the PID‐Ms spectra was significantly increased (Figure 2b and Table S1). High‐resolution XPS of C 1s spectrum of PID‐Ms shown in Figure S2a consists of three peaks that corresponded to C─C or C─H (284.6 eV), C═N─OH (285.7 eV), and C═O (288.6 eV) species. The high‐resolution XPS of the N 1s spectrum of PID‐Ms in Figure S2b has two emerging peaks, which were attributed to C─N─C (399.4 eV) and C═N (400.5 eV) species. The high‐resolution XPS of the O1s spectrum of PID‐Ms in Figure S2c has three emerging peaks, which were attributed to C═O, C═N─OH, and C─O species, further illustrating that the PID‐Ms were successfully synthesized. One characteristic diffraction peak of the crystalline structure of typical polystyrene was observed at 19.6° in all the obtained x‐ray diffraction (XRD) spectra, suggesting that the crystal structure was well maintained after the radiation‐induced and chemical modification, and grafting polymerization and other reactions may occur mainly in the non‐crystalline region of PS‐Ms (Figure S2d). Thermogravimetric analysis (TGA) was performed to investigate the thermal stabilities of PID‐Ms. The PS‐Ms showed high thermal stabilities up to 305.6°C, without any residue at 461.2°C (Figure S3a). Compared with PHEA‐Ms (Figure S3b), the TGA curves of the PAN‐Ms (Figure S3c) and PID‐Ms (Figure S3d) revealed three main decomposition processes (approximately 200°C–300°C, 300°C–400°C, and 400°C–472°C), due to the complex thermal decomposition of the grafted polymer chains and PS. This result confirmed that the conjugated PID ligands are thermally stable in the conventional temperature range corresponding to TARE therapy.

The scanning electron microscopy (SEM) images reveal well‐dispersed, and uniform sizes for PS‐Ms with an average size of 40.02 µm (Figure S4a,b). The SEM images show similar polymer morphologies for the pristine PAN‐Ms (Figure S4c) and the PID‐Ms (Figure 2d) with monodisperse micron‐sized microspheres with good sphericity, indicating that the initial microstructure of the polymer was not damaged during the radiation chemical modification process. The gas displacement method was employed to measure the true density of PID‐Ms as 1.15 g/mL. These results suggest that the radio‐particle diameter and density of the PID‐Ms were consistent with the clinical size requirement of radioactive microspheres for transarterial radioembolization (TARE). The density of PID‐Ms (1.15 g/mL) is close to that of blood (1.05 g/mL), they are more uniformly distributed within the bloodstream and therefore potentially more uniformly distributed throughout the tumor vascular bed. Furthermore, compared with the energy dispersion spectroscopy (EDS) mapping of PS‐Ms (Figure S4d), EDS mapping of C, O, and N for PAN‐Ms indicated the successful grafting polymerization (Figure S4e). The EDS mapping of PID‐Ms showed that conjugated PID ligands were widely distributed entire surface of the PID‐Ms (Figure 2e).

Before the thermal experiments (i.e., the radiometallic labeling experiments), a full‐scale cold test is required to facilitate the effective achievement of results of practical value. Complexation kinetics experimental of PID‐Ms was performed in an initial Lu^3+^, Y^3+^, and Ho^3+^ concentration of 10 ppm. As shown in Figure 2c, the complexation kinetics process begins with rapid complexation of metals during the initial 20 min; the adsorption capacity (Q) of Lu^3+^, Y^3+^, and Ho^3+^ reaches 33.53 mg‐Lu/g‐PID‐Ms, 29.08 mg‐Y/g‐PID‐Ms, 14.00 mg‐Ho/g‐PID‐Ms, respectively, followed by a slow complexation period, indicating the radioactivity of Radiometals‐PID‐Ms can be regulated to meet the needs of clinical radiotherapy. These results indicate that the metal complexation kinetics of the PID‐Ms involves chemical adsorption; which confirms that PID‐Ms can label ^177^Lu, ^90^Y, and ^166^Ho. Furthermore, compared with the SEM image and EDS mapping of PID‐Ms, the morphology of PID‐Ms complexation with Lu^3+^, Y^3+^, and Ho^3+^ did not change significantly, and the EDS mapping results showed that the metal ions covered the surface of PID‐Ms uniformly (Figure 2f,g, and Figure S5a). The XPS spectra of the Lu‐PID‐Ms displays two new Lu 4d peaks at 196.7 and 206.5 eV are assigned to Lu 4d_5/2_ and Lu 4d_3/2_, respectively, and show that the valence of bound Lu was not changed (Figure S5b, Table S1). Compared with high‐resolution XPS spectra for PID‐Ms, the position of C─N─C and C═N─O was shifted to lower binding energy, indicating the increase in electron density for N atoms (Figure 2h,i and Figure S5c). This demonstrated that the Lu mainly interacted with not only C─N─C but also C═N─O of the conjugated PID ligand.

### 2.2 Radiolabeling and Radiostability of the Radiometal‐PID‐Ms

**FIGURE 3:**
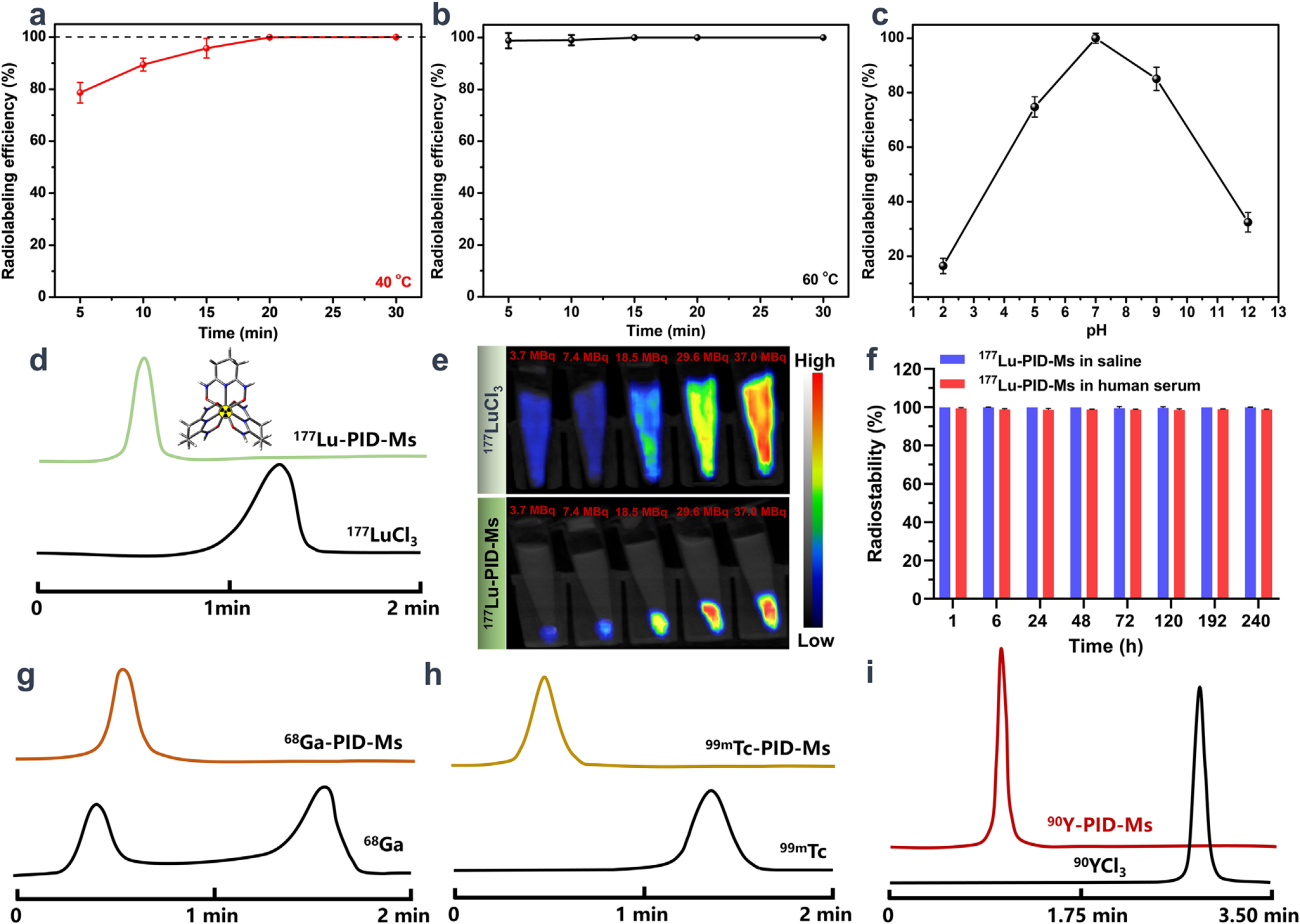
Complexation kinetics assay and radiostability of ^177^Lu‐PID‐Ms. Radiolabeling efficiency of ^177^Lu‐PID‐Ms as a function of labeling time at (a) 40°C and (b) 60°C, respectively. (c) Radiolabeling efficiency of ^177^Lu‐PID‐Ms as a function of pH. (d) Radio‐TLC analysis of free ^177^LuCl_3_ and ^177^Lu‐PID‐Ms. (e) 3D reconstruction SPECT/CT images for ^177^LuCl_3_ and ^177^Lu‐PID‐Ms dispersed in saline with radioactivity ranging from 1.85 to 37 MBq. (f) Radiostability of the ^177^Lu‐PID‐Ms in saline and human serum at different contacting periods. (g) Radio‐TLC analysis of free ^68^GaCl_3_ and ^68^Ga‐PID‐Ms. (h) Radio‐TLC analysis of free Na^99m^TcO_4_ and ^99m^Tc‐PID‐Ms. (i) Radio‐TLC analysis of free ^90^YCl_3_ and ^90^Y‐PID‐Ms.

To quickly obtain the target radioactivity of ^177^Lu‐PID‐Ms, a quantitative study was designed using different radiolabeling temperatures, pH, and time. The radiolabeling efficiency was tested by thin‐layer chromatography (TLC). At room temperature, the complexation kinetics of ^177^Lu with PID‐Ms was slow, and low labeling efficiency (<40.00%) could be obtained after 30 min of radiolabeling (Figure S6a). When the temperature was 40°C, the labeling efficiency of ^177^Lu‐PID‐Ms increased with increasing reaction time, and the labeling efficiency reached 99.99% after 20 min of labeling (Figure 3a). Significantly higher than that of ^177^Lu‐PHEA‐Ms and ^177^Lu‐PAN‐Ms (lower than 48.00%), indicating PID ligand strong binding ^177^Lu (Figure S6c,d). When the labeling temperature was 60°C, PID‐Ms had fast complexation kinetics with ^177^Lu, and the labeling efficiency reached 98.80% after 5 min of labeling (Figure 3b). The experimental results verified that PID‐Ms had fast complexation kinetics performance on radiometals at lower temperatures (40°C–60°C).

As shown in Figure 3c,d, the radiolabeling efficiency of ^177^Lu‐PID‐Ms increased rapidly and linearly with the increase of pH and reached a steady state of about 99.99% at pH 7. At high pH (pH 9–12), the free ^177^Lu would form precipitation with hydroxide ions leading to a decrease in labeling efficiency. Similarly, the radiolabeling yield of ^90^Y‐PID‐Ms increases rapidly in a linear manner with increasing pH. The radiolabeling yields at pH 3 and 5 are 21.2% and 55.8%, respectively, while the highest radiolabeling yield of 99.7% is achieved at pH 7 (Figure S7a). ^177^Lu having not only β‐ or emission auger electrons for achieving therapy, but also emitting several accompanying signals γ‐208 keV (11%) and 113 keV (6.4%) photons for diagnostic evaluation and dosimetry, suitable for in vivo SPECT imaging. SPECT/CT image showing the SPECT signal intensity increased with the radioactivity of ^177^Lu‐PID‐Ms. The result indicates that ^177^Lu‐PID‐Ms can be applied to SPECT/CT imaging in vivo and quantified accurately (Figure S6b). Furthermore, free ^177^Lu ions in ^177^LuCl_3_ was dispersed in saline, while ^177^Lu in the ^177^Lu‐PID‐Ms were bound to the PID‐Ms and was at the bottom of EP tube as the microspheres settled, indirectly indicating that ^177^Lu‐PID‐Ms have high radiostability (Figure 3e). These results suggest that SPECT/CT can track and localize ^177^Lu‐PID‐Ms in vivo and is a non‐invasive imaging method.

The radiostability of ^177^Lu‐PID‐Ms and in vitro was investigated by the amount of free ^177^Lu shed from the ^177^Lu‐PID‐Ms obtained by radioactivity counting in saline and human serum. As shown in Figure 3f, after shaking at 37°C for 240 h in saline, less than 0.01% of free ^177^Lu was detected from the ^177^Lu‐PID‐Ms, and the radiostability performance in human serum is almost similar to that in saline, indicating that ^177^Lu‐PID‐Ms has ultra‐high radiostability in saline and human serum. Furthermore, not only ^177^Lu but also ^90^Y‐ PID‐Ms exhibited ultra‐high radiostability. Less than 0.01%free ^90^Y was detected in for shaking at 37°C for 168 h in saline solution (Figure S7b). The ^177^Lu‐PID‐Ms and ^90^Y‐PID‐Ms complex exhibits excellent kinetic and thermodynamic inertness in saline and human serum.

Meanwhile, the radiostability properties of PID‐Ms with ^68^Ga, ^99m^Tc, and ^188^Re were investigated. The radiolabeling efficiencies of ^68^Ga‐PID‐Ms (Figure 3g), ^99m^Tc‐PID‐Ms (Figure 3h), and ^188^Re‐PID‐Ms (Figure S7c) were found to be 99.99%, 99.98%, and 99.99%, respectively. Furthermore, under the conditions of 1 mol/L sodium citrate as an unfolding agent, the radiolabeling efficiency of ^90^Y‐PID‐Ms remains at 99.99% (Figure 3i). This further confirms the stable radiolabeling of the conjugated PID ligand with ^90^Y, which is consistent with the results obtained from non‐radiometal Y^3+^ labeled PID‐Ms. The above results of PID‐Ms labeling with various radio‐metals confirmed the broad‐spectrum labeling ability of PID ligands with different radioactive metals, and the corresponding radiometal‐PID complexes have ultra‐high radiostability properties and excellent kinetic and thermodynamic inertness, which led to the experimental and theoretical investigation of the stability mechanism in a later paper (see below).

### 2.3 Coordination Mechanism and Thermodynamic Stabilities of the Radiometals‐PID Complexes

**FIGURE 4:**
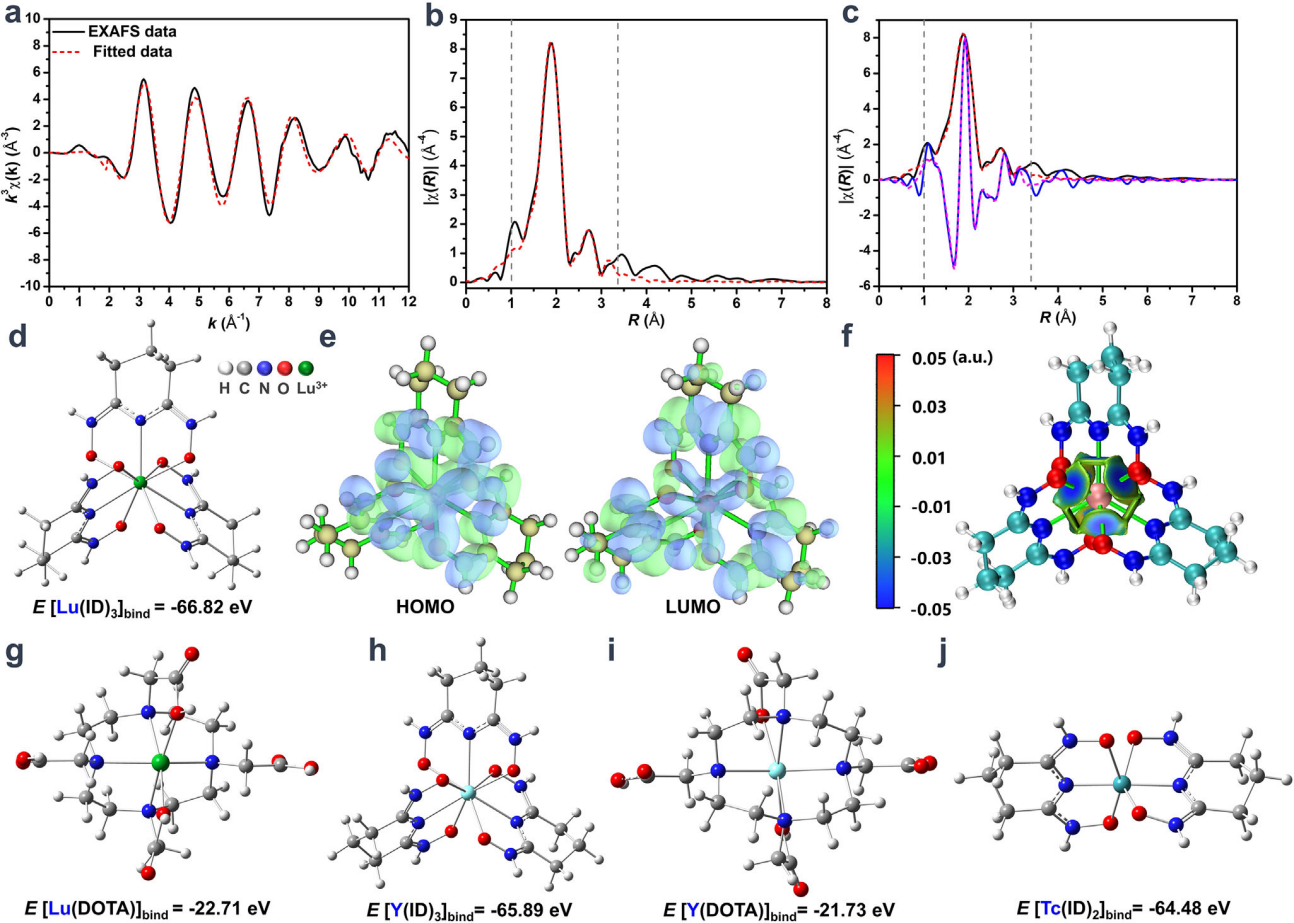
In situ XAFS analyses of the ^177^Lu‐PID‐Ms reveal the mechanism of the coordination mode of Lu^3+^ and comparison of the binding energy with gold standard Lu‐DOTA complex. (a) Lu *L*_III_‐edge *k* ^3^‐weighted EXAFS spectra (black line) and the best theoretical fits (red dotted line) of the Lu‐PID‐Ms. (b) Corresponding nonphase shift corrected Fourier transforms. EXAFS data (black line) and fitted data (red dotted line) of the Lu‐PID‐Ms in *R*‐space. (c) EXAFS analysis of the *R*‐space data and fitting results of the Lu‐PID‐Ms. The upper plot is the magnitude of the Fourier transform, while the lower plot is the real‐space component of the Fourier transform. (d) A nine‐coordinate 3: 1 [Lu(ID)_3_] complex with mixed coordination of the three imide dioxime ligands with O,N,O‐tridentate ultra‐stable chelation, and the binding energy is up to 66.82 eV. (e) HOMO and LUMO distribution on the [Lu(ID)_3_] complex. (f) IGMH maps of [Lu(ID)_3_] complex. VMD renders IGMH quantitative analysis data to obtain a visualization of the interaction intensity between of Lu and conjugated PID ligand. The bond critical points and bond paths corresponding to Lu─N/O interactions are also shown in the IGMH image. (g) A six‐coordinate 1: 1 Coordination configuration of gold standard [Lu(DOTA)] complex, and the binding energy is 22.71 eV. (h) Coordination configuration of [Y(ID)_3_] complex, and the binding energy is 65.89 eV. (i) Coordination configuration of [Y(DOTA)] complex, and the binding energy is 21.73 eV. (j) Coordination configuration of [Tc(ID)_2_] complex, and the binding energy is 64.48 eV.

The coordination environment of the Lu‐PID complex was first studied by in situ x‐ray absorption fine structure (XAFS) spectra, which provide metrical information about the interatomic interactions between Lu and the PID ligands. Notably, Figure S8 reveals the XAFS oscillation modes of Lu^3+^ at the *L*_III_‐edge of the Lu‐PID complex in *k* space. The Lu^3+^ absorption edge in the Lu‐PID complex is found at 9251.24 eV, and the maximum energy is 9285.76 eV, which suggests the coordination of Lu onto the PID‐Ms. The upper limit of the selected effective function of lutetium ion reaches 12 Å^−1^, and the large range of the selected effective function ensures the accuracy and reliability of the fitting results (Figure 4a). *R*‐space spectrum shows that in addition to the main peak between 1 and 2.3 Å, there are peaks of moderate intensity between 2.5 and 3.3 Å, indicating that Lu^3+^ in the mixed‐ligand environment has a more ordered coordination structure at a long distance. Therefore, we build a complex fitting model with the multi‐shell structure for Lu^3+^ in *R*‐space fitting to finally get the information on the scattering path, coordination number, bond length, etc. Among them, the average distance between Lu^3+^ and O atoms of the first coordination shell layer is 2.34 Å, which can be attributed to the strong complexation of O in the PID ligand, and the O coordination number of the first shell layer is six, which can be determined that Lu^3+^ is surrounded by six O atoms are involved in the Lu^3+^ coordination in a distorted octahedral geometry. The scattering path of the second coordination shell layer reveals that the disorder of imine N is higher than that of oxime N, indicating that imine N exists as a coordination atom to participate in Lu^3+^ coordination, and imine N has a high electron‐giving ability to strongly bind Lu^3+^ (Figure 4b, Table S2). Moreover, the quantitative XAFS fit data were in high agreement with the experimental XAFS data in the selected fit range (1 to 3.3 Å) (Figure 4c).

The density functional theory (DFT) calculation of the Lu‐PID complex shows that the coordination mechanism fits well with the result of the XAFS analysis. The binding energy (*E*_bind_ = −66.82 eV) were obtained via DFT calculation indicating a nine‐coordinate Lu‐PID complex with mixed coordination of the three ID ligands to form a tridentate (O,N,O‐tridentate chelate coordination) conjugated configuration, and the planar structure of PID converts to a 3D tridentate chelated structure, in which three ID chains are bridged by metal to form a covalent conformational interlocked network with a diameter of ∼1 nm, oriented along the PID chain direction (Figure 4d and Figure S9a). Furthermore, the results of the front‐line orbital analysis of the Lu‐PID complex molecule confirm that the off‐domain π state of the PID ligand structure is significantly coupled to the orbital of Lu, and the electron highest occupied state (HOMO) orbital is dispersed throughout the Lu‐PID complex molecular system, indicating that there is a significant π‐electron conjugation between the PID ligand and the Lu^3+^ center, and this π‐electron off‐domain effect can reduce the electron cloud density thus further enhance the kinetic and thermodynamic inertness of the radiometal‐PID complexes (Figure 4e). The independent gradient model based on Hirshfeld partition (IGMH)was employed to analyze the coordination bond interaction strength of the PID ligand of the configuration with Lu [30]. As shown in Figure 4f, it can be seen from all the intermolecular interactions revealed by the IGMH maps, that it is noteworthy that the *δg^inter^* values on the Lu─O/N bond range from −0.03 to −0.05 a.u., indicating that the PID has strong chemical bonding with Lu.

Binding energy studies for gold standard ^177^Lu‐DOTA complex (*E*_bind_ = −22.71 eV) by DFT calculation further reveal that the thermodynamic inertness of ^177^Lu‐PID complex (*E*_bind_ = −66.82 eV) is better than that of the ^177^Lu‐DOTA complex (Figure 4g and Figure S9b). As shown in Figure 4h,i, DFT calculations were performed for the ^90^Y‐PID complex and the ^90^Y‐DOTA complex under the same generalized and basis group conditions, and the results showed that the binding energy of the ^90^Y‐PID complex (*E*[Y(ID)_3_]_bind_ = −65.89 eV) is more than three times that of the ^90^Y‐DOTA complex (*E*[Y(DOTA)]_bind_ = −21.73 eV), and even the binding energy of the ^90^Y‐ID single ligand complex (*E*[Y(ID)]_bind_ = −32.40 eV) and the tridentate‐like complex (*E*[Y(ID)_2_]_bind_ = −52.19 eV) are also much larger than the binding energy of the ^90^Y‐DOTA complex (Figure S10a,b). Furthermore, the binding energy of the ^99m^Tc‐PID complex is nearly four times higher than that of the ^99m^Tc‐DOTA complex, up to 64.48 eV (Figure 4j), and even the binding energy of the ^99m^Tc‐ID single ligand complex is much higher than that of the ^99m^Tc‐DOTA (Figure S11a,b). These results encouraged us to investigate the binding energy for a large number of metal isotopes, including therapeutic metal radionuclides for nuclear medicine applications and magnetic resonance imaging (MRI) probes for metallic elements. As shown in Figures S12–S24, the binding energies of the ^68^Ga‐PID, ^188^Re‐PID, ^223^Ra‐PID, ^213^Bi‐PID, ^225^Ac‐PID, ^166^Ho‐PID, ^44^Sc‐PID, ^153^Sm‐PID, ^64^Cu‐PID, ^111^In‐PID, ^89^Zr‐PID, Fe‐PID, and Gd‐PID complexes are all much higher than the corresponding ^68^Ga‐DOTA, ^188^Re‐DOTA, ^223^Ra‐DOTA, ^213^Bi‐DOTA, ^225^Ac‐DOTA, ^166^Ho‐DOTA, ^44^Sc‐DOTA, ^153^Sm‐DOTA, ^64^Cu‐DOTA, ^111^In‐DOTA, ^89^Zr‐DOTA, Fe‐DOTA, and Gd‐DOTA complexes, respectively (Figure S25). These results once again confirm that the PID ligands have excellent labeling kinetic and thermodynamic inertness compared with the conjugate complexes formed by the gold standard DOTA ligands after chelating the metal radionuclides, which benefitted from the formation of covalent bond conformational locks between the radiometal and the ligand atoms in the PID ligands, thus constructing an ultra‐radiostability radiometal‐covalent conformational interlocked crystal network. In addition, the radiometal chelate ligand‐mediated cross‐linking between the PID ligand molecular chains enhances the mechanical properties as well as thermodynamic inertness of the conjugated system. Moreover, the PID ligands have universal metal‐radionuclide labeling, which is an important milestone in the development of nuclear medicine.

Well‐designed polymeric PID ligand molecules not only chelate radiometals but also firmly bind the decay product metals of radiometals, enhancing the biosafety of PID ligand‐linked radiopharmaceuticals, especially alpha‐emitting metal radionuclides. As an example, ^177^Lu is decayed 100% to ^177^Hf. The FTIR spectra of ^177^Hf‐PID‐Ms show a shift of the corresponding C─NH─C and N─O group peaks to lower wave numbers, indicating a strong chemical binding interaction of the PID ligand with ^177^Hf (Figure S26a). The SEM images of the ^177^Hf‐PID‐Ms showed that the regular and spherical with good monodispersity (Figure S26b), and the EDS mapping results showed a uniform distribution of ^177^Hf elements on the surface of PID‐Ms (Figure S26c). The results indicated that PID‐Ms could bind ^177^Lu decay products, which could further improve the in vivo safety of the organisms.

### 2.4 Cytotoxicity and Biocompatibility of PID‐Ms

**FIGURE 5:**
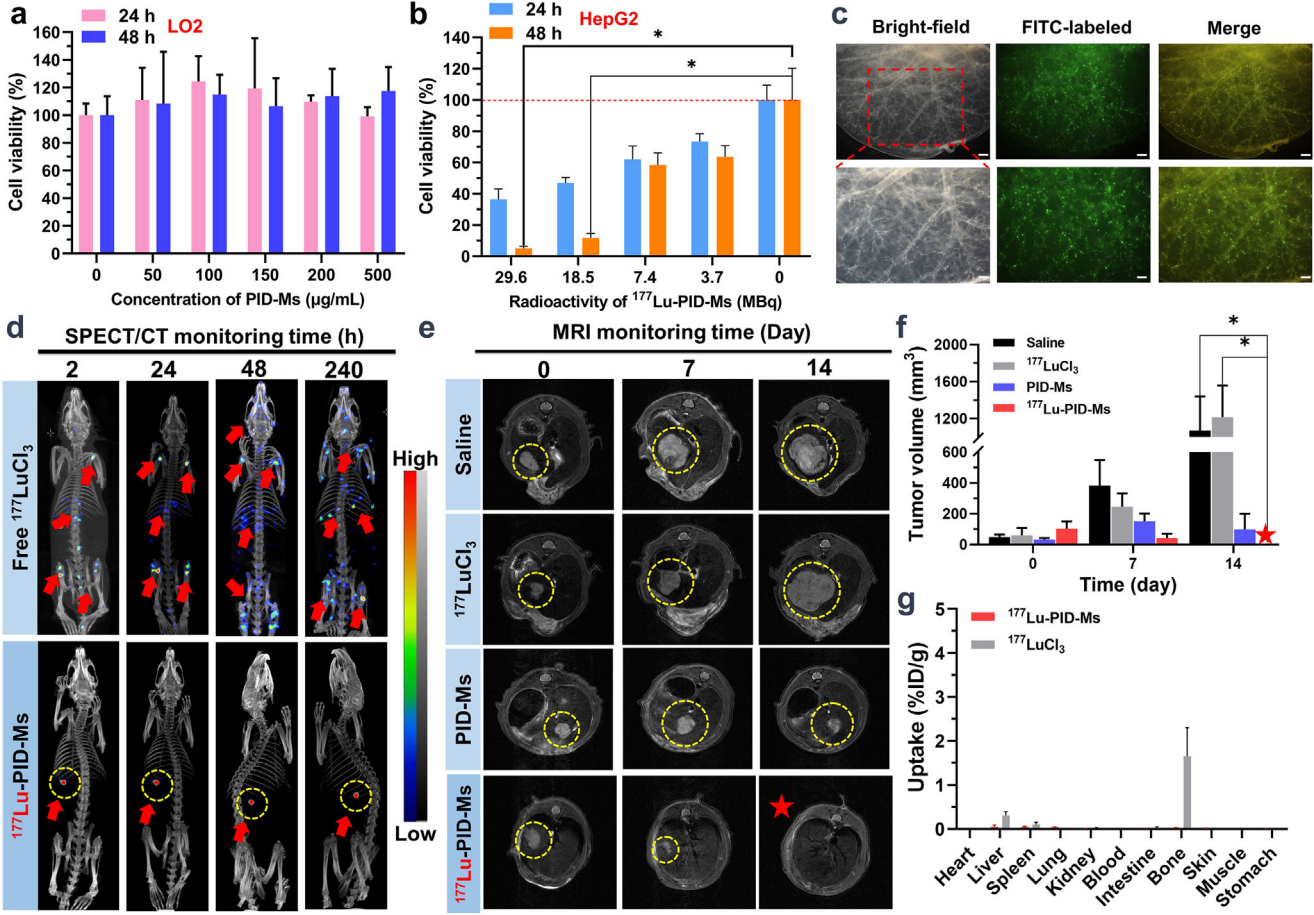
In vitro and In vivo TARE treatment ^177^Lu‐PID‐Ms for rat N1S1 orthotopic live tumor model. (a) Cell viability for LO2 cells after incubation with different concentrations of PID‐Ms suspension for 24 and 48 h, respectively. (b) Cell viability for HepG2 cells after incubation with different radioactivity ranging from 0 to 29.6 MBq of ^177^Lu‐PID‐Ms suspension for 24 and 48 h, respectively. (c) Fluorescence stereomicroscope images of intact rat decellularized liver models after injection of FITC‐labeled PID‐Ms at different magnifications, showing that the PID‐Ms can embolize into the distal end vessels of rat liver. Scale bar: 200 µm. (d) Representative SPECT/CT 3D‐imaging depicting biodistribution of free ^177^LuCl_3_ and ^177^Lu‐PID‐Ms in the orthotopic liver tumor‐bearing rat at different monitoring times after TARE. The red arrows indicate the location of the tissue uptake for ^177^Lu. (e) Representative MRI imaging of tumors after precision delivery of the saline, free ^177^LuCl_3_, PID‐Ms, and ^177^Lu‐PID‐Ms for 0, 7, and 14 days. The yellow dashed circles show the location of the tumors. (f) Tumor volume of rat N1S1 orthotopic liver tumor model after therapy of the saline, free ^177^LuCl_3_, PID‐Ms, and ^177^Lu‐PID‐Ms, respectively. (g) The biodistribution of free ^177^LuCl_3_ group and ^177^Lu‐PID‐Ms group in the orthotopic liver tumor‐bearing rat (*n* = 3) for 10 days after TARE. All the data are presented as mean ± SD (*n* = 3). Statistical significances were calculated via Welch's t‐test or Mann−Whitney U test. *p < 0.05, **p < 0.01, and ***p < 0.001.

The cytotoxicity of PID‐Ms on Human normal liver cells (LO2) and ^177^Lu‐PID‐Ms on human hepatocellular carcinoma cells (HepG2) were evaluated by cell counting kit‐8 (CCK‐8) assay before in vivo studies. The CCK‐8 results validated the proliferative activity of LO2 between different concentrations of PID‐Ms (Figure 5a). The LO2 cells maintained more than 100% viability even when the concentration of PID‐Ms was increased to 500 µg/mL and incubated for 48 h. However, the ^177^Lu‐PID‐Ms induced significant cytotoxicity against HepG2 at the same concentration of PID‐Ms under different radioactivities (Figure 5b).

In vitro coagulation and hemolysis experiments verified the hemocompatibility of PID‐Ms. There was almost no coagulation of PID‐Ms compared with the positive control calcium chloride solution, indicating that PID‐Ms did not cause coagulation (Figure S27a). Furthermore, the results of the hemolysis test indicated that PID‐Ms had low hemolysis values, similar to saline and PBS, which were both below 2% (Figure S27b). Coagulation and hemolysis assays showed that PID‐Ms do not have procoagulant or hemolytic properties and may exhibit comparable hemocompatibility with saline and PBS in blood. Thus, PID‐Ms have good biocompatibility and non‐toxicity and have a promising application as a radiopharmaceutical precursor material. We established an in vitro evaluation model of the acellular liver of SD rats to determine the distribution behavior of PID‐Ms in the vascular bed. The fluorescein isothiocyanate (FITC)‐labeled PID‐Ms were injected into the transparent hepatic vascular bed, and exhibit effectively deposit and embolize the corresponding vascular bed, especially the distal peripheral vessels of the liver (Figure 5c).

Cy5.5‐labeled PID‐Ms were injected into rabbit VX2 tumor lesions via catheter arterial embolization, and the heart, liver, spleen, lung, and kidney as well as the tumor were dissected and removed for in vivo imaging system (IVIS) imaging. A clear fluorescence signal was observed in the liver tumor lesions, and the fluorescence signal was not observed in the heart, spleen, lung, and kidney, indicating that PID‐Ms could exist stably in the tumor lesions without shunting to other normal organs (Figure S27c). Subsequently, PID‐Ms were injected into the right suprarenal artery of normal pigs for vascular embolization via femoral artery puncture and transcatheter arterial embolization under DSA mediation (Figure S27d). Iohexol contrast agent was injected before and after embolization to observe the vascular embolization effect, respectively. DSA images showing the PID‐Ms had been successfully injected into the vascular bed of the right superior renal artery of normal pigs, which impeded the passage of the iohexol contrast agent to some extent, resulting in the disappearance of vascular visualization (Figure S27e).

### 2.5 SPECT Studies and TARE Therapeutic Effect Monitoring for Radiometal‐PID‐Ms

The anti‐tumor effect, SPECT imaging performance, and in vivo kinetic stability of ^177^Lu‐PID‐Ms was investigated by constructing a rat N1S1 liver tumor model (Figure S28). As shown in Figure 5d, for the free ^177^LuCl_3_ and ^177^Lu‐PID‐Ms rats, SPECT/CT imaging was performed at 2, 24, 48, and 240 h after drug administration, respectively. Representative 3D‐imaging SEPCT images showed that the free ^177^LuCl_3_ group had the highest uptake of ^177^Lu in the bone joint cavity, followed by its shoulder, hip, spine, skull, and abdominal cavity sites. Furthermore, the representative 3D‐imaging SEPCT images of the ^177^Lu‐PID‐Ms group showed that the enrichment in the liver tumor lesion area only and was maintained from 2 to 240 h after drug administration, and no ^177^Lu uptake was observed in other normal organ tissues throughout the body. Compared with free ^177^LuCl_3_ and blank control, the liver tumors in the ^177^Lu‐PID‐Ms group completely disappeared after 14 days for therapy, and the MRI images indicated that ^177^Lu‐PID‐Ms had a significant anti‐tumor effect (Figure 5e,f). To accurately quantify the distribution of ^177^Lu‐PID‐Ms in rat major organs, we measured the biodistribution of ^177^Lu‐PID‐Ms in the orthotopic liver tumor‐bearing rat after 14 days of therapy. The biodistribution results largely confirmed the results of the SPECT imaging study. Compared to free ^177^LuCl_3_ group, the heart, liver, spleen, lung, stomach, bone, muscle, and intestine of rats in the ^177^Lu‐PID‐Ms group showed almost no uptake after 14 days (Figure 5g). H&E staining for the heart, liver, spleen, lung, and kidney tissues revealed that no inflammation and injury was observed (Figure S29). These results confirmed that ^177^Lu‐PID‐Ms had excellent anti‐tumor effects, SPECT imaging performance, and in vivo kinetic inertness.

**FIGURE 6:**
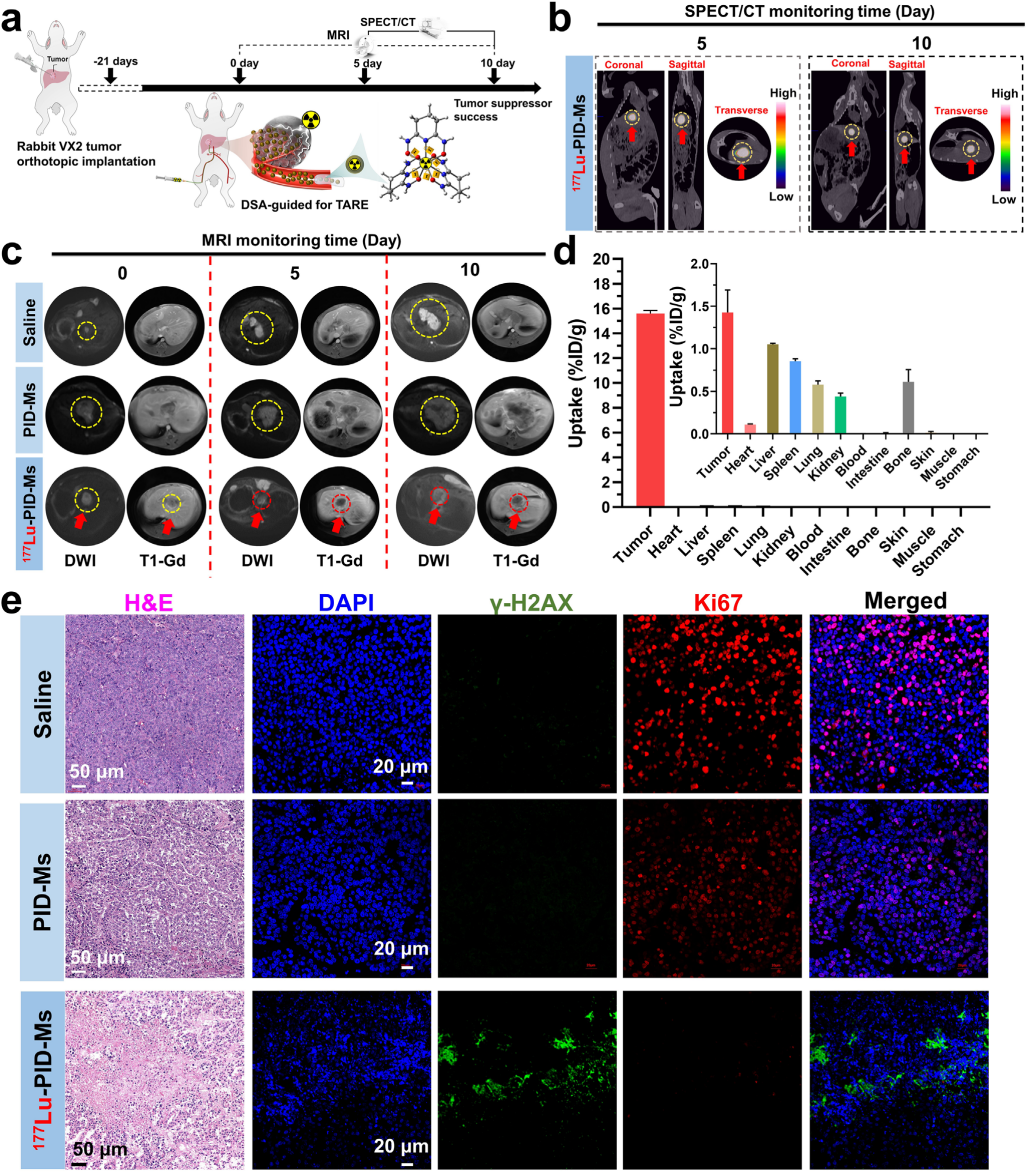
In vivo radiostability and anti‐tumor efficacy in rabbit VX2 orthotopic live tumor model evaluation for ^177^Lu‐PID‐Ms. (a) Schematic illustration shows the TARE therapeutic procedure via DSA‐guided precision delivery of ^177^Lu‐PID‐Ms in orthotopic live VX2 tumor‐bearing rabbits. (b) Representative SPECT/CT images of orthotopic liver VX2 tumor‐bearing rabbit at 5 and 10 days after DSA‐guided precision delivery of ^177^Lu‐PID‐Ms for tumor TARE therapy. (c) Representative MRI imaging of orthotopic liver VX2 tumor‐bearing rabbit after therapy with the saline, PID‐Ms, and ^177^Lu‐PID‐Ms for 0, 5, and 10 days. The yellow and red dashed circle shows the location of the orthotopic VX2 liver tumor. (d) Biodistribution study of ^177^Lu‐PID‐Ms and free ^177^LuCl_3_ (Inset) in orthotopic liver VX2 tumor‐bearing rabbit for 10 days after TARE therapy. (e) H&E, γ‐H2AX, and Ki67 staining of representative resected tumor lesions in orthotopic liver VX2 tumor‐bearing rabbit model after therapy with saline, PID‐Ms, and ^177^Lu‐PID‐Ms, respectively, at 10 days.

The ^177^Lu‐PID‐Ms were further used as a radiopharmaceutical for DSA‐mediated TARE treatment of orthotopic live VX2 tumor‐bearing rabbits to evaluate the therapeutic efficacy, SPECT imaging performance, and in vivo kinetic inertness (Figure 6a). Free ^177^LuCl_3_, PID‐Ms, and ^177^Lu‐PID‐Ms were injected into orthotopic live VX2 tumor‐bearing rabbits via catheter for TARE treatment under the guidance of DSA, respectively. The microcatheter was entered into the hepatic artery and then injected with an iohexol contrast agent, and the location, size, and vascular supply of the tumor could be observed by DSA imaging (Figure S30). After the injection of free ^177^LuCl_3_ solution, the vessels can still be clearly shown under injection of iohexol contrast agent. In contrast, after injection of PID‐Ms or ^177^Lu‐PID‐Ms, the tiny blood supply vessels of the tumor disappeared from the visualization, indicating that the material completely entered the tumor blood supply vascular bed. Compared to the high uptake of ^177^Lu in the major organs of rabbits in the free ^177^LuCl_3_ group, and representative SPECT/CT images of the ^177^Lu‐PID‐Ms group showed high uptake of ^177^Lu in the tumor lesion area except ^177^Lu uptake was not observed in any other normal organs and tissues, confirming the excellent in vivo kinetic and thermodynamic inertness as well as ultra‐radiostability of ^177^Lu‐PID‐Ms (Figure 6b and Figure S31a). Compared to the free ^177^LuCl_3_ group and the blank control group, MRI images of the ^177^Lu‐PID‐Ms group showing the rabbit VX2 liver tumors were effectively inhibition (Figure 6c and Figures S30b and S31b). Furthermore, the quantitative biodistribution results confirmed the results of the SPECT imaging study, where the heart, liver, spleen, lung, kidney, blood, intestine, stomach, bone, muscle, and skin of rabbits in the ^177^Lu‐PID‐Ms group were almost free of ^177^Lu uptake after 10 days compared to the free ^177^LuCl_3_ group, and the radioactive uptake values in the tumor tissues were as high as 15.60%ID/g, and tumor‐to‐normal liver uptake (T/N) ratio was high reach 192.6 (Figure 6d). Clinical trials have shown that the T/N ratio is the single best prognostic indicator for tumor progression and patient response. The greater the T/N ratio, the faster the patient responds to the big tumor‐absorbed dose, and the longer the patient's survival time [31, 32]. However, the radioactive uptake value of the tumor tissue in VX2 in situ liver cancer rabbits treated with free ^177^LuCl3 was only 1.18% ID/g, and the T/N ratio was 1.4, which is much lower than the T/N ratio of ^177^Lu‐PID‐Ms group (192.6). As seen in the H&E‐stained tumor tissue sections, the ^177^Lu‐PID‐Ms treatment group exhibited significant tumor cell injury and apoptosis compared to the Saline group, ^177^LuCl_3_ group, and PID‐Ms group. Moreover, the Ki‐67 immunohistochemical staining showed the highest proliferation rate of rabbit VX2 liver tumor cells in the Saline group and free ^177^LuCl_3_ group, and tumor cells in the ^177^Lu‐PID‐Ms treatment group were almost not proliferating. From the results of γ‐H2AX immunohistochemical staining, it was found that ^177^Lu‐PID‐Ms killed tumor cells mainly by damaging the DNA of tumor cells through radioactive β particles (Figure 6e and Figure S31c). Interestingly, because the PID ligands are rich in oxime and imine groups, which facilitate the coupling of various organic dyes, ^177^Lu‐PID‐Ms can be found to be regularly ordered and stable within the tumor tissues (Figure S32). After H&E staining of the heart, liver, spleen, lung, and kidney of all groups of rabbits, no significant cellular damage and inflammation were found in normal organ tissues of all groups of rabbits, except for the organ tissues of free ^177^LuCl_3_ group, where significant inflammation and cellular damage occurred (Figure S33). These results confirm that ^177^Lu‐PID‐Ms can achieve effective internal radiotherapy for rabbit VX2 liver tumors by TARE treatment, which benefit from the formation of a covalent bond conformational lock between the metal radioisotope and the ligand atoms in the PID ligand, thus constructing ultra‐radiostability radiometal‐covalent conformational interlocked network. The radiometal‐PID‐Ms offers an effective platform with ultra‐radiostability for image‐guided effectively inhibit tumor cell proliferation and cause tumor cell apoptosis.

## 3 Discussion

PID‐Ms radioactive metal chelators can be used to simultaneously label one or two or more radioactive metals to obtain new functional radioactive polymeric microspheres. PID‐Ms possess outstanding physicochemical properties, including good monodispersity, flawless sphericity, excellent mechanical properties, outstanding irradiation resistance, close to blood density, simple synthesis process and can be prepared on a large scale for quantitative production. Thanks to the excellent thermodynamic and kinetic inertness of PID‐Ms in vitro and in vivo after radiometallic labeling, Radiometals‐PID‐Ms can be further combined with nuclear medicine imaging technology to achieve SPECT or PET imaging in cancer diagnosis and treatment. Therefore, PID‐Ms can be independent from the double restrictions of radioactive metal types and high flux neutron reactors in nuclear medicine departments. According to the needs of clinicians and patients, the PID‐Ms functional carriers can be labeled with ^177^Lu, ^90^Y, ^188^Re, ^166^Ho, ^99m^Tc, ^68^Ga and other diagnostic radioactive metals, and can be prepared into the following radioactive microsphere products: (1) ^177^Lu‐PID‐Ms, ^188^Re‐PID‐Ms, and ^166^Ho‐PID‐Ms, which can be used for the diagnosis and treatment of intermediate and advanced hepatocellular carcinoma; (2) ^99m^Tc‐PID‐Ms and ^68^Ga‐PID‐Ms, which can be used for SPECT or PET imaging to assess the patient's hepatopulmonary and hepatogastric shunts and determine the suitability of TARE and the required radiation dose; (3) ^90^Y‐PID‐Ms can also be prepared for the treatment of intermediate and advanced liver cancer; (4) ^90^Y/^99m^Tc‐PID‐Ms and ^90^Y/^68^Ga‐PID‐Ms can be prepared by simultaneously labeling PID‐Ms with dual radioactive metals for the quantitative assessment of precise tumor absorbed dose during the treatment of intermediate and advanced liver cancer (Figure 1b).

Radiometals‐PID‐Ms has controllable shelf life, convenient transportation at home and abroad, does not rely on high flux neutron reactor irradiation, can be labeled and dispensed at any time according to clinical needs (to achieve “current matching”), and its radioactivity can be adjusted according to tumor size and tumor blood supply, which can ensure the therapeutic effect of advanced liver cancer and tumor progress can be controlled. At the same time, Radiometals‐PID‐Ms can not only benefit patients with advanced liver cancer through diagnosis and treatment of TARE or TARE combined with chemotherapy and immunotherapy, but also achieve intratumoral radiotherapy in situ through direct intratumoral injection. In recent years, researchers have carried out a large number of experimental studies on the treatment of liver tumor, kidney tumor and head and neck tumor with radioactive microspheres, and made some preclinical research progress [33, 34, 35, 36, 37]. This direct intra‐tumoral injection can also be used for other tumor types, including pancreatic tumors and even lung tumors. Therefore, Radiometals‐PID‐Ms has a wide range of clinical applications. The clinical application of Radiometals‐PID‐Ms intravascular radioembolization may significantly improve the survival rate of patients with advanced liver cancer and connect more patients to descending treatment procedures such as liver transplantation and surgical resection. In particular, radiation lobectomy and radiation segmental lobectomy are expected to develop the future clinical application of Radiometals‐PID‐Ms (especially high absorbed dose> 400 Gy) from palliative treatment to transformational and radical therapy. The preclinical basic studies have confirmed that Radiometals‐PID‐Ms has a very high potential for clinical transformation, and now we are continuing to promote the clinical transformation process of the results of this work.

Currently, targeted radionuclide therapy dependent on the location of the target (cell surface, cytosol, nucleus, specifically RNA and DNA) result in objective responses in only 30%–60% of patients. Nuclide‐labeled small molecules, nanoparticles, peptides, and/or antibodies are administered intravenously to enhance the in vivo blood circulation time for targeted intra‐tumor irradiation therapy, but the lack of tumor perfusion resulting from the radiopharmaceutical with low tumor aggregation, heterogeneity of target expression on the tumor cell surface and/or antigens, and the type of nuclide used to lead to poor clinical oncologic outcomes [38]. Furthermore, the low degree of radiopharmaceutical retention in tumor tissue (due to the residence time of some small molecular imaging and therapeutic agents in tumor tissue is short and the molecules can be easily dispersed) may limit efficacy, and the relative lack of specific targets for clinically aggressive cancers, leading to a particularly poor prognosis [38, 39]. Different from other forms of brachytherapy, the radioactive sources are placed within tumor tissue, precision delivery of radioactive microspheres via the hepatic artery branches results in primary and metastatic tumors of the liver being targeted irrespective of drug resistance, independent of a target, and the drug absorption rate is nearly 100%. The accurate delivery of tumoricidal radiation doses to the primary and metastatic tumors protected healthy liver tissue from irradiation [24, 40]. TARE therapeutics are of increasing interest for the treatment of vascular‐rich solid tumors. ^90^Y glass microspheres and ^90^Y resin microspheres have all garnered FDA approval for selectively delivering the radioactive sources to the tumor while sparing healthy tissues.

Our study shows that the polymeric PID ligands have multiple radiometallic chelating universals, and the Radiometals‐PID complexes have excellent ex vivo thermodynamic inertness. Our polymeric PID ligand materials are designed to be easy to use in nuclear medicine with medical metal nuclides including ^99m^Tc, ^90^Y, ^177^Lu, ^188^Re, ^166^Ho, etc., on a “ready‐to‐use” basis. Among them, ^99m^Tc‐PID‐Ms and ^177^Lu‐PID‐Ms can be used for lung shunt and gastric shunt before TARE and precise assessment of patient's required activity dose; ^90^Y‐PID‐Ms and ^188^Re‐PID‐Ms can be used for TARE therapeutics. Furthermore, the polymeric PID ligands can be flexibly designed as small molecule ligands, dimeric ligands, block copolymer ligands, and nanoparticle polymeric ligands for nuclear medicine treatment of cancer and as MRI contrast agents. A direct comparison of our PID‐Ms platform with clinically approved TARE microspheres (Table S3) and with conventional radiolabeling strategies (Table S4) clearly demonstrates its advantages in terms of labeling mildness, nuclide versatility, and in vivo stability.

We have rationally designed and synthesized a conjugated PID polymeric ligand for strong binding of therapeutic and imaging radionuclides (^177^Lu, ^90^Y, ^99m^Tc, ^68^Ga, ^223^Ra, and ^188^Re), and obtained an ultra‐radiostability radionuclide mediated covalent conformational interlocked network. The thermodynamic and kinetic inertness of Radiometals‐PID complexes is superior to that of the gold standard Radiometal‐DOTA complexes, which was verified by XAFS experimental and DFT calculations. Notably, the conjugated PID polymeric ligand with the advantage of mild and rapid radiolabeling, which is important for the subsequent development of conjugated small molecule PID ligands, conjugated dimeric ligands, conjugated PID block copolymer ligands, and conjugated PID nanoparticle polymeric ligands for binding to heat‐sensitive biological proteins. The radiolabeled complexes of conjugated PID ligands with ^177^Lu, ^90^Y, ^99m^Tc, and ^68^Ga showed high radiolabeling efficiency (all > 99.90%). In vitro saline and human serum stability studies demonstrated the excellent kinetic and thermodynamic inertness of ^177^Lu‐PID‐Ms and ^90^Y‐PID‐Ms. Importantly, the radiometal‐mediated covalent conformational interlocked network in the Radiometals‐PID complexes ensures the structural stability of the complexes, even in complex organisms. The results of ^177^Lu‐PID‐Ms for TARE treatment in rat and rabbit orthotopic live tumor models presented here are of proof‐of‐principle that ^177^Lu‐PID‐Ms were successfully radioembolized at the hepatic artery with a good and stable localization distribution in the liver, indicating a good in vivo biodistribution and biosafety, showing that radionuclide‐mediated covalent conformational interlocked network with excellent antitumor effect and in vivo kinetic thermodynamic inertness. Moreover, the N‐/O‐mixed donor macromolecular polymeric ligands described in this study offer great flexibility in design, and can be tailored as small molecular ligands, block copolymer ligands, and polymeric nanoparticle for the theranostic treatment of a wide range of diseases. Very encouraging results suggest that this interesting conjugated polymeric chelator has a high degree of metal radionuclide labeling universality and mild labeling conditions that allow it to bind to heat‐sensitive antibodies or other thermally degradable bioproteins, indicating this conjugated polymeric PID ligand chelator is very promising and may be useful for SPECT imaging (^177^Lu and ^99m^Tc), PET imaging (^68^Ga), radiotherapy (^177^Lu, ^90^Y, and ^188^Re,), and MRI probes opening new application possibilities.

Despite the promising results, several limitations of the current PID‐Ms system should be acknowledged. First, the mean diameter of PID‐Ms (∼40 µm) may not be optimal for tumors supplied by very small‐caliber capillaries (<20 µm), potentially leading to incomplete embolization in certain microvascular beds. Second, while the PID polymer backbone exhibits excellent radiostability over the experimental timeframe (14 days), its long‐term (>6 months) degradation profile and the corresponding fate of the degraded products in vivo remain to be elucidated. Third, although no acute or subacute toxicity was observed, the potential for chronic radiation‐induced fibrosis or local inflammation due to permanent microsphere retention requires careful evaluation in large‐animal models with extended follow‐up. Finally, the current multi‐step synthesis of PID‐Ms, although scalable, could benefit from further optimization to reduce production complexity and cost. Addressing these limitations will be an important direction for our future studies toward clinical translation.

## 4 Experimental Section

### 4.1 Chemicals and Reagents

Styrene, azobisisobutyronitrile (AIBN), polyvinyl alcohol, 2‐hydroxyethyl acrylate (HEA), acrylonitrile (AN), ceric ammonium nitrate, N, N‐dimethylformamide (DMF), sodium carbonate (Na_2_CO_3_), Dimethylsulfoxide (DMSO), hydrochloric acid (HCl), nitric acid (HNO_3_), hydroxylamine hydrochloride (NH_2_OH·HCl), phosphate‐buffered saline (PBS), ethanol, lawrencium trichloride (LuCl_3_), yttrium trichloride hexahydrate (YCl_3_), Holmium(III) chloride (HoCl_3_), Tin(II) chloride (SnCl_2_), sodium citrate, and methanol were purchased from Sinopharm Chemical Reagent Company. All chemicals from commercial sources were reagent grade and used without further purification. Nitrogen gas (99.99% purity) was obtained from Shanghai Louyang Gas Canned Co., Ltd. ^177^LuCl_3_ solution (pH 1.5) and ^90^YCl_3_ was purchased from Chengdu Mo Sen Fu Yuan Technology Co., Ltd. The Na^99m^TcO_4_ solution was provided from The First Affiliated Hospital of Xiamen University. The ^68^Ge−^68^Ga generator was used to elute ^68^GaCl_3_ (IREElit, Belgium). Na^188^ReO_4_ solution was provided from Shanghai Institute of Applied Physics, Chinese Academy of Sciences. Pentobarbital sodium was purchased from Lulong Biotechnology Co., Ltd. (Shanghai, China). iohexol was purchased from Jiangsu Hengrui Medicine Co., Ltd. Interventional medical devices were available from the Radiology Department of Xiang'an Hospital of Xiamen University.

### 4.2 Preparation of PS‐Ms

The PVA solution was prepared as a stabilizer for the monodisperse suspension emulsion polymerization reaction by weighing 30 g of PVA and dissolving it in 600 mL of deionized water, which was added into a 1 kg glass reactor and stirred at 200 rpm/min for 6 h to form a homogeneous system. Subsequently, the system temperature was raised to 80°C by passing N_2_ bubble for 20 min, and then a mixture of styrene (8 wt.%) and AIBN (0.5 wt.%) was added dropwise (0.8 mL/min) by syringe pump, and the stirring speed was kept constant, and the reaction was terminated by natural cooling under N_2_ atmosphere for 3 h to obtain a white emulsion. The white emulsion was centrifuged and settled to remove the supernatant, and the bottom microspheres were cleaned by adding anhydrous ethanol for ultrasonication, then centrifuged and settled again to remove the supernatant, and the above operation was repeated three times, and finally the white powdered PS‐Ms was obtained by vacuum drying for 24 h.

### 4.3 Preparation of PHEA‐Ms

The PS‐Ms (200 g) was cleaned and dried with deionized water, put into PE self‐sealing bags, fixed with tape on an iron irradiation stand, and placed in a cobalt source (^60^Co irradiation source) for γ‐ray irradiation at a dose rate of 1.7 kGy/h and an absorbed dose of 50 kGy under room temperature and air conditions, and the irradiated PS‐Ms was stored in a refrigerator at −20°C. Then, 40 wt.% of HEA monomer and 60 wt.% of deionized water were added to a 1 kg glass reactor, and the speed was adjusted to 200 rpm/min and stirred at room temperature for 30 min, followed by N_2_ bubbling for 30 min to remove oxygen. After the reaction, the reaction product was centrifuged and settled at 6000 rpm/min to remove the supernatant, and the bottom modified microspheres were washed with deionized water to remove the homopolymer and residual HEA monomer by ultrasonication, and then the operation was repeated five times by centrifugal settling and washing, and finally PHEA‐Ms was obtained by vacuum drying for 24 h.

### 4.4 Preparation of PAN‐Ms

The reaction solution was prepared in a 1 kg glass reactor and weighed PHEA‐Ms was added to the reaction solution containing 40 wt.% of AN and 60 wt.% of DMF. First, the oxygen in the reaction solution was removed by bubbling with N_2_ for 20 min. Then, a certain amount of ceric ammonium nitrate solution (1 mol/L ceric ammonium nitrate dispersed in HNO_3_) was quickly added to the reaction system. Cerium ammonium nitrate was used to initiate the covalent grafting polymerization of AN monomer with PHEA‐Ms at a certain water bath temperature. After the reaction, the covalently grafted PAN‐Ms were taken out and centrifuged at 6000 rpm/min to remove the supernatant reaction solution, and the bottom modified material was washed by ultrasonication with DMF to remove the homopolymer and residual AN monomer, and then the operation was repeated five times by centrifugal sedimentation and washing, and the PAN‐Ms were dried under vacuum for 24 h.

### 4.5 Synthesis of PID‐Ms

Add a certain amount of NH_2_OH·HCl to the beaker, then add a mixture of deionized water and anhydrous ethanol (1:1), stir until completely dissolved, add a certain amount of Na_2_CO_3_, adjust the pH of the reaction solution to 7.0, transfer the prepared reaction solution to a 1 kg glass reactor with PAN‐Ms, adjust the rotation speed to 200 rpm/min. The reaction system was stirred at room temperature for 30 min to mix well, and then the temperature was adjusted to 60°C. After reaction, the reaction product was centrifuged and settled at 6000 rpm/min to remove the supernatant reaction solution, and deionized water was added to ultrasonically clean the modified material on the bottom layer, and then the operation was repeated five times with centrifugal settling and deionized water washing to remove the residual NH_2_OH, and vacuum drying for 24 h. The yellow powder PID‐Ms was obtained.

### 4.6 Radiosynthesis of Radiometals‐PID‐Ms

In this work, the radiolabeling efficiencies of ^177^Lu, ^90^Y, ^99m^Tc, ^68^Ga, and ^188^Re labeled PID‐Ms were investigated. The effects of labeling time at different labeling temperatures and different pH values on the radiolabeling efficiency of ^177^Lu‐labeled PID‐Ms (^177^Lu‐PID‐Ms) were investigated. The effects of different pH values on the radiolabeling efficiency of ^90^Y‐labeled PID‐Ms (^90^Y‐PID‐Ms) were investigated.

We verified the effect of labeling time at different labeling temperatures on the radiolabeling efficiency of ^177^Lu‐PID‐Ms. The specific experimental steps were: 1 mL of PID‐Ms (10 mg/mL) was added to the EP tube, the PID‐Ms dispersion solution (deionized water) was removed by centrifugation, and then 10 µL of ^177^LuCl_3_ solution (37 MBq) was added, due to ^177^LuCl_3_ solution contained 0.04 M HCl, so another 10 µL of NaOH solution with a concentration of 0.04 M was added to the EP tube to neutralize the HCl and mixed well, and the reaction was immediately shaken at room temperature for 5, 10, 15, 20, and 30 min before sampling for Radio‐TLC detection method, with silica gel glass fiber strips as support; the unfolding agent was 1 mol/L sodium citrate solution; The samples were spotted on the silica glass fiber strips about 1 cm away from the bottom, and then the silica glass fiber strips were unfolded in a glass vial containing the unfolding agent, and when the front of the unfolding agent reached 8 cm, the silica glass fiber strips were removed and dried, and then the ^177^Lu‐PID‐Ms radiolabeling efficiency was measured with a Mini‐Scan thin‐layer chromatography scanner. Under the same conditions, samples were taken after shaking the reaction at 40°C and 60°C for 5, 10, 15, 20 and 30 min, respectively, to measure the ^177^Lu‐PID‐Ms radiolabeling efficiency by Radio‐TLC assay. We verified the effect of different pH values on the radiolabeling efficiency of ^177^Lu‐PID‐Ms. 1 mL of PID‐Ms (10 mg/mL) was added to each of the five EP tubes, the PID‐Ms dispersion solution (deionized water) was removed by centrifugation, and then 10 µL of ^177^LuCl_3_ solution (37 MBq) was added separately, due to Since the ^177^LuCl_3_ solution contained 0.04 M HCl, 10 µL of NaOH solution with a concentration of 0.04 M was added to the EP tube to neutralize the HCl and mixed well, followed by 1 mL of deionized water solution with pH 2, 5, 7, 9, and 12, respectively, and then shaken well and immediately shaken at 40°C for 20 min before sampling and analyzed by the above TLC assay. The efficiency of ^177^Lu‐PID‐Ms radiolabeling was measured.

The detailed experimental procedure for ^177^Lu labeling of PHEA‐Ms (^177^Lu‐PHEA‐Ms) and PAN‐Ms (^177^Lu‐PAN‐Ms), respectively, was as follows: 1 mL of PHEA‐Ms (10 mg/mL) and PAN‐Ms (10 mg/mL) were added to 2 EP tubes, respectively, and the dispersion of PHEA‐Ms and PAN‐Ms was removed by centrifugation solution, then 10 µL of ^177^LuCl_3_ solution (37 MBq) and 10 µL of 0.04 M NaOH solution were added to neutralize the HCl and mixed well, followed by 1 mL of deionized water solution with pH 7, respectively. After shaking well, the reaction was immediately shaken at 40°C for 20 min and then the samples were analyzed and measured for ^177^Lu‐PHEA‐Ms and ^177^Lu‐PAN‐Ms radiolabeling efficiency by the above TLC assay.

The detailed experimental procedure for ^90^Y‐labeled PID‐Ms (^90^Y‐PID‐Ms) was as follows: 1 mL of PID‐Ms (10 mg/mL) was added to each of the four EP tubes, the dispersion solution of PID‐Ms was removed by centrifugation, and then 1 mL of 0.2 mol/L acetate buffer solution with pH 3, 5, and 7 was added to the EP tubes. Add 10 µL of ^90^YCl_3_ solution (18.5 MBq) solution to each EP tube with different pH values. Since the ^90^YCl_3_ solution contained 0.04 M HCl, another 10 µL of NaOH solution with a concentration of 0.04 M was added to each EP tube and shaken well. The reaction was immediately shaken and sonicated at 40°C for 20 min, and the supernatant was taken after centrifugation to measure and calculate the radiolabeling efficiency of ^90^Y‐PID‐Ms by liquid scintillation counter. Meanwhile, the samples were taken by TLC assay with 1 mol/L sodium citrate solution as the unfolding agent. The radiolabeling efficiency of ^90^Y‐PID‐Ms was measured by a radioactive thin‐layer chromatography scanner (Eckert & Ziegler, model: B‐MS‐2000FP).

The detailed experimental procedure for ^99m^Tc‐labeled PID‐Ms (^99m^Tc‐PID‐Ms) was as follows: 1 mL of PID‐Ms (10 mg/mL) was added to an EP tube, the dispersion solution of PID‐Ms was removed by centrifugation, and then 1 mL of deionized water solution with pH 7 was added, followed by the addition of Na^99m^TcO_4_ solution (37 MBq) and SnCl_2_ (1 mg/mL, 0.1 mol/L HCl) were added and mixed well, and the reaction was immediately shaken at 40°C for 20 min and then sampled by TLC. The support was a silica glass fiber strip; the unfolding agent was 1 mol/L sodium citrate solution; the sample was taken and spotted on the silica glass fiber strip about 1 cm from the bottom, and then the silica glass fiber strip was placed into the glass vial with the unfolding agent. When the front of the unfolding agent reached 8 cm, the silica glass fiber strips were dried and the ^99m^Tc‐PID‐Ms radiolabeling efficiency was measured with a Mini‐Scan thin‐layer chromatography scanner.

The detailed experimental procedure for ^68^Ga‐labeled PID‐Ms (^68^Ga‐PID‐Ms) was as follows: 1 mL of PID‐Ms (10 mg/mL) was added to the EP tube, the dispersion solution of PID‐Ms was removed by centrifugation, and then 1 mL of deionized water solution with pH 7 was added, and then ^68^GaCl_3_ solution (37 MBq) diluted from ^68^Ge‐^68^Ga generator was added separately. The sample was immediately shaken at 40°C for 20 min. The support was a silica glass fiber strip; the unfolding agent was 1 mol/L sodium citrate solution; the sample was spotted on the silica glass fiber strip about 1 cm from the bottom, and then the silica glass fiber strip was unfolded in the glass vial with the unfolding agent, and when the front of the unfolding agent reached 8 cm, the silica glass fiber strip was removed. The glass fiber strips were dried and the ^68^Ga‐PID‐Ms radiolabeling efficiency was measured with a Mini‐Scan thin‐layer chromatography scanner.

The detailed experimental procedure for ^188^Re‐labeled PID‐Ms (^188^Re‐PID‐Ms) was as follows: 1 mL of PID‐Ms (10 mg/mL) was added to the EP tube, the dispersion solution of PID‐Ms (deionized water) was removed by centrifugation, and then 1 mL of deionized water solution with pH 7 was added, followed by the addition of Na^188^ReO_4_ solution (37 MBq) and SnCl_2_ (1 mg/mL, 0.1 mol/L HCl) respectively and mixed well. The reaction was immediately shaken at 40°C for 20 min and then sampled by TLC detection method with silica gel glass fiber strips as support; the unfolding agent was 1 mol/L sodium citrate solution. The radiolabeling efficiency of ^188^Re‐PID‐Ms was measured with a radioactive thin‐layer chromatography scanner (Eckert & Ziegler, model: B‐MS‐2000FP). The conditions for each radionuclide PID‐Ms labeling strategy are summarized in Table S5.

### 4.7 Physiochemical Characterization

A certain amount of PS‐Ms, PAN‐Ms, and PID‐Ms were weighed separately for FT‐IR (Bruker Vertex 70V) testing. In the transmission mode, the scanning range was 4000–500 cm^−1^, the resolution was 4 cm^−1^, and the scanning frequency was 32 times.

The XPS (Thermo Scientific ESCALAB Xi+) spectra were performed by weighing 0.02 g of PS‐Ms, PAN‐Ms, PID‐Ms and Lu‐PID‐Ms, respectively, with a test voltage of 10 kV, a full‐spectrum flux energy of 160 eV, a scan range of 0–1200 eV and an analysis chamber pressure of 1 × 10^−9^ Torr. The XPS data were processed and analyzed by XPS peak 4.1 software.

0.5 g of PS‐Ms, PAN‐Ms, PID‐Ms, Lu‐PID‐Ms, and Hf‐PID‐Ms were weighed separately for XRD testing.

The PS‐Ms, PAN‐Ms, and PID‐Ms were weighed at 0.02 g and placed in the thermogravimetric analyzer for testing. The thermal degradation curves of each sample were measured under N_2_ atmosphere with an N_2_ flux of 20 mL/min, a measurement temperature range of 40°C–800°C, and a temperature rise rate of 10°C·min^−1^.

The PS‐Ms, PAN‐Ms, PID‐Ms, Lu‐PID‐Ms and Hf‐PID‐Ms were dried in an oven at 60°C for 6 h before the test, and a certain amount of PS‐Ms and PCMs were weighed and fixed on the sample table with conductive adhesive, and the surface was treated with conductivity (gold spray: current 20 mA, 150 s), and the morphological changes of the microspheres were observed under SEM After the microspheres were photographed, the voltage was increased to 15 KV and energy dispersive x‐ray spectroscopy (EDS) was performed to obtain the elemental distribution of the samples.

The true density of PID‐Ms was tested by gas displacement method, the specific steps were: firstly, the volume of the empty tube was tested; with helium as the medium, the free volume of the sample was measured after the gas displacement pressure equilibrium, and the difference between the front and back volumes was the volume of PID‐Ms. Finally, the true density of PID‐Ms could be obtained according to the ratio of the mass of PID‐Ms and the volume of PID‐Ms.

### 4.8 PID‐Ms Complexation of Non‐Radioactive Metals Experiment

At room temperature, 10 mg of PID‐Ms were added to 100 mL of aqueous solutions of Lu^3+^, Y^3+^, and Ho^3+^ at an initial concentration of 10 ppm in a triangular flask, where the pH of the non‐radioactive metal solutions of Lu^3+^, Y^3+^, and Ho^3+^ was adjusted to 6–7 using 0.5 mol/L NaOH and 0.5 mol/L HCl, respectively, before the addition of PID‐Ms. The complexation reaction was carried out by placing the triangular flask into a preheated 40°C water bath and adjusting the rotational speed to 200 rpm/min. The precipitate was centrifuged after 10, 20, and 30 min, respectively. 1 mL of supernatant was added to 2% HNO3 solution with a pipette gun. Subsequently, the initial concentration of each metal ion and the metal ion concentration at different time points of complexation were determined by inductively coupled plasma emission spectrometry (ICP‐OES), and the complexation capacity of PID‐Ms on non‐radioactive metals was determined using the following equation.

$$Q=\frac{\left(C_{0}-C_{t}\right)\times 0.1}{W}$$

where Q is the complexation capacity of PID‐Ms on non‐radioactive metals at different complexation time points (unit: mg‐Metal/g‐PID‐Ms, i.e., how many mg of non‐radioactive metals are complexed per 1 g of PID‐Ms), *C*_0_ and *C*_t_ are the initial concentration of non‐radioactive metals and the concentration after complexation at different time points (unit: mg/L, measured by ICP‐OES), respectively; 0.1 is the volume (L) of the non‐radioactive metal solution.

### 4.9 Radiostability of ^177^Lu‐PID‐Ms and ^90^Y‐PID‐Ms

^177^Lu‐PID‐Ms in vitro saline and human serum radioactivity stability experiments: ^177^Lu‐PID‐Ms were divided into two groups of three parallels each, and the radioactivity of ^177^Lu‐PID‐Ms was 18.5 MBq for each parallel sample. Two milliliters of saline and human pure serum were added to the two groups (human pure serum was sourced from the author of this paper, Xiao Xu) and subsequently placed into 37°C shaker, and then ^177^Lu‐PID‐Ms suspension and supernatant were taken at 1, 6, 24, 48, 72, 120, 192, and 240 h, respectively. Subsequently, the results of the radioactivity stability of ^177^Lu‐PID‐Ms in saline and human pure serum were obtained by analyzing the assay with a γ‐counter.

In vitro saline and fetal bovine serum (FBS) radiostability experiments of ^90^Y‐PID‐Ms: ^90^Y‐PID‐Ms were divided into two groups of three parallels each, and the radioactivity of each parallel sample of ^90^Y‐PID‐Ms was 18.5 MBq. The samples of both groups were added to 1 mL of saline and 5% FBS, respectively, and subsequently placed in a 37°C water bath and shaken, and then the supernatants were centrifuged at 24, 96, and 168 h, respectively, and the radiostability of ^90^Y‐PID‐Ms was measured and calculated by liquid scintillation counter (Perkin Elmer, USA).

### 4.10 SPECT imaging performance of the ^177^Lu‐PID‐Ms

SPECT/CT imaging of ^177^Lu‐PID‐Ms and ^177^LuCl_3_ with different radioactivities (3.7, 7.4, 18.5, 29.6, and 37.0 MBq) were performed after mixing with saline in EP tubes, respectively. After centrifugal precipitation, SPECT/CT imaging was performed with a nanoScan‐SPECT/CT scanner (Mediso, Hungary).

### 4.11 Investigation of the Mechanism for Radiometals‐PID Complexes With Ultra‐Radiostability

This work combines extended x‐ray absorption fine structure (EXAFS) experiments, the independent gradient model based on Hirshfeld partition (IGMH), front‐line molecular orbital analysis, and DFT calculations to elucidate the mechanism for ^177^Lu‐PID‐Ms with ultra‐radiostability. The ultra‐radiostability mechanism of ^177^Lu‐PID‐Ms was investigated from single atom (EXAFS experiments) and molecular level (IGMH front‐line molecular orbital analysis), and the specific experimental steps are as follows: EXAFS spectra were collected on the Taiwan Synchrotron Radiation Research Center's Taiwan Light Source 21A x‐ray nano‐diffraction beamline station for EXAFS spectral acquisition of Lu‐PID‐Ms, where the photon flux range was 1 × 10^11^–3 × 10^9^ photons/s and the x‐ray energy was 6–27 keV. The EXAFS data were analyzed using the Demeter software package (Athena and Artemis programs), and the backscattering amplitudes and phase shifts were calculated using the FEFF 956 program. The Fourier transform (FT) was performed on the k3‐weighted EXAFS oscillations. A window of 1.0–3.3 Å in R‐space was used for curve fitting of the FT‐EXAFS data. The fine structure parameters of Lu‐PID‐Ms, such as coordination number (CN), interatomic bond length (R), Debye–Waller factor (σ^2^), energy zero shift (Δ*E_0_*), and scattering amplitude intensity (amp), were obtained by the fitting.

To improve the efficiency of the DFT calculation, a repeating unit in the PID polymer ligand and DOTA are chosen as the calculation models, respectively. In order to compare the kinetic inertness of the PID polymer ligand with that of the commercially used DOTA ligand bound to a radiometal, this study used DFT to calculate binding energies of ^177^Lu‐PID, ^90^Y‐PID, ^68^Ga‐PID, ^99m^Tc‐PID, ^188^Re‐PID, ^223^Ra‐PID, ^213^Bi‐PID, ^225^Ac‐PID, ^166^Ho‐PID, ^44^Sc‐PID, ^153^Sm‐PID, ^64^Cu‐PID, ^111^In‐PID, ^89^Zr‐PID, Fe‐PID, Gd‐PID, and ^177^Lu‐DOTA, ^90^Y‐DOTA, ^68^Ga‐DOTA, ^99m^Tc‐DOTA, ^188^Re‐DOTA, ^223^Ra‐DOTA, ^213^Bi‐DOTA, ^225^Ac‐DOTA, ^166^Ho‐DOTA, ^44^Sc‐DOTA, ^153^Sm‐DOTA, ^64^Cu‐DOTA, ^111^In‐DOTA, ^89^Zr‐DOTA, Fe‐DOTA, Gd‐DOTA.

In addition, DFT calculations were implemented in Gaussian 09 using the B3LYP generalized level [41, 42], which has been widely used and proved to be sufficiently accurate for a wide range of systems. 6–31G(d) basis groups were used for H, C, N, and O atoms; among them, Lu, Y, Tc, Ga, Re, Ra, Bi, Ac, Ho, Sc, Sm, Cu, In, Zr, Fe, and Gd SDD pseudopotential basis sets were used for geometry optimization and frequency analysis. Frequency calculations were performed to verify the minimum energy of the obtained geometric configurations. The binding energy (*E*_bind_) of the complexes is calculated as follows:

$$E_{bind}=E\left(Metal-ligands\right)-E\left(Metal\right)-E\left(ligands\right)$$

where *E*(Metal‐ligands) represents the total energy of the complex (Metal‐PID or Metal‐DOTA), *E*(Metal) is the energy of the metal, and *E*(ligands) is the energy of the ligand (PID or DOTA). The effect of aqueous solvation was established by using the integral equation formulation of the polarization continuum model (IEF‐PCM) for a single‐point energy calculation of the previously optimized geometric configuration. To further analyze the structural stability of the optimized complexes obtained by EXAFS experiments combined with DFT calculations, the strength of the coordination bonds was analyzed by IGMH method [30]. The IGMH quantitative analysis was implemented by Multiwfn 3.7 [43], while the visualization of the interaction strength was obtained by the VMD 1.9.3 program [44]. The electronic highest occupied (HOMO) and electronic lowest unoccupied (LUMO) states of the molecular orbitals of the complexes were also calculated and analyzed simultaneously by first calculating the frequencies of the coordination configurations and subsequently processing them analytically by Multiwfn 3.7 to obtain the frontline molecular orbitals.

### 4.12 Cytotoxicity Analysis

Different concentrations of PID‐Ms were prepared and assayed for toxicity to human normal hepatocytes (LO2) cells (RRID: CVCL_6926) from the American Type Culture Collection (ATCC, Manassas, VA, USA). The cell viability was measured using Cell Counting Kit‐8 (CCK‐8) after co‐incubation with LO2 cells (5000 cells/well) in 96‐well plates for 24 h and 48 h using pre‐sterilized PID‐Ms at different concentrations (0, 20, 50, 100, 150, 200, and 500 µg/mL). The specific experimental procedure was as follows: 96‐well plates were spread with LO2 cells, 5000 cells per well. The cells were incubated in a CO_2_ incubator at 37°C for 24 h. After 24 h of incubation, the medium was changed to one containing the corresponding concentration of PID‐Ms and incubated for another 24 h. CCK‐8 solution was added to the 96‐well plates, and 10 µL of CCK‐8 solution was added per 100 µL of the medium. Incubate in a 37°C incubator for 2 h. Measure the optical density at 450 nm (OD450) of each well with a microplate reader and calculate the corresponding cell viability. All experiments were performed in 4 groups in parallel.

### 4.13 Radioactive Killing Assay of Human Liver Cancer Cells

We examined the killing effect of PID‐Ms with different radioactivities on human hepatocellular carcinoma cells (HepG2) cells (RRID: CVCL_0027) from ATCC. After co‐incubation with HepG2 cells (5000 cells/well) in 96‐well plates for 24 h and 48 h using ^177^Lu‐PID‐Ms of different radioactivities (0, 3.7, 7.4, 18.5, and 29.6 MBq) prepared in advance in the long half‐life laboratory. The viability of HepG2 cells was measured using CCK‐8 (Cell Counting Kit‐8). HepG2 cells were plated in 96‐well plates with 5000 cells per well. The cells were incubated in a CO_2_ incubator at 37°C for 24 h. After 24 h of incubation, the medium was replaced with a medium containing ^177^Lu‐PID‐Ms at the corresponding activity level and incubated for another 24 h. CCK‐8 solution was then added to the 96‐well plates, with 10 µL of CCK‐8 solution per 100 µL of the medium. The optical density at 450 nm (OD450) values of each well were measured using with a microplate reader and the corresponding cell viability was calculated.

### 4.14 Clotting Time and Hemolysis Test

#### 4.14.1 Clotting Time Test

PBS (50 µL), saline (9 g/L, 50 µL), calcium chloride (0.1 mol/L, 50 µL) solution, and PID‐Ms were added to 96‐well plates. Subsequently, 50 µL of human whole blood was added to each well. The plate was rinsed with saline at selected time points to stop clotting, and the liquid was repeatedly washed and withdrawn until the solution became clear, indicating that all soluble blood components were removed. After the test was completed, the clotting time and clot formation in each well were recorded using OPPO Find X3 cell phone photography to obtain the results of whether PCMs prompted clotting in human whole blood.

#### 4.14.2 Hemolysis Test

Three milliliters of human whole blood was taken into a centrifuge tube, washed with 1% sodium heparin, mixed with an equal amount of saline, and then centrifuged. The supernatant was removed, and the precipitate was mixed with saline, and the above procedure was repeated three times to obtain the erythrocyte suspension. Saline (100 µL), deionized water (100 µL), and PID‐Ms were added to a 96‐well plate, and 100 µL of erythrocyte suspension solution was added to each well, and the above steps were repeated 6 times, and the plate was placed in a 37°C incubator for 2 h before being removed and centrifuged. The supernatant of each well was taken 100 µL in a new well and used to measure the optical density (OD) at 540 nm to calculate the hemolysis rate. All experiments using human blood from healthy adult volunteers were carried out with informed consent and were approved by the Medical Ethics Committee of the Tenth Affiliated Hospital, Southern Medical University (Dongguan People's Hospital) (No. KYKTSB2025‐032).

### 4.15 Distribution of PID‐Ms in Decellularized Liver Vessels

A rat decellularized liver model was prepared and the intravascular distribution of fluorescein isothiocyanate (FITC)‐labeled PID‐Ms (FITC‐PID‐Ms) in the rat liver was evaluated. First, after complete excision of the normal rat liver, the portal and inferior vena cava of the rat liver was washed with prepared 0.5% to 1% sodium dodecyl sulfate (SDS) solution using a peristaltic pump (speed: 4 mL/min) for 12 h until the liver was translucent, and then rinsed with saline to remove residual SDS. Subsequently, PID‐Ms were mixed with a concentration of 1 mg/mL FITC was mixed well and reacted at 40°C for 2 h to obtain FITC‐PID‐Ms. Finally, 0.6 mL of FITC‐PID‐Ms (20 mg/mL) was slowly injected into the inferior vena cava of rat decellularized liver using a 1 mL syringe, respectively, and the images were immediately captured by fluorescence microscopy.

### 4.16 Renal Artery Embolization on Normal Pigs

The experiments were performed with the permission of the Xiamen University Animal Care and Use Committee and approved by the Xiamen University Animal Experimentation Ethics Committee (No. XMULAC20210102). Healthy Yorkshire pigs weighing approximately 25–30 kg were purchased from Shanghai Jagan Biotechnology Co., Ltd. in Shanghai, China. Yorkshire pigs were allowed a 2‐week acclimatization period at the Xiamen University Animal Center. A 5F arterial sheath was placed via femoral artery puncture under DSA guidance, followed by the introduction of a 2.1F microcatheter and a coaxial microguide wire, followed by iohexol injection for renal arteriography to locate the renal arterial vascular bed in pigs. An amount of PID‐Ms was manually pushed through the microcatheter into the right kidney of the pig at a uniform and slow rate. After administration, iohexol was injected in DSA subtraction mode to detect the effect of renal vascular embolization, and DSA imaging images were collected.

### 4.17 TARE Treatment of Rats Orthotopic Live Tumor Model

The experiments were performed with the permission of the Xiamen University Animal Care and Use Committee and approved by the Xiamen University Animal Experimentation Ethics Committee (No. XMULAC20210102). Wistar rats (100–120 g, no specific pathogens, RRID: RGD 13508588) were used as experimental animals. When the cultured rat hepatocellular carcinoma N1S1 cells (RRID: CVCL_3552) proliferated to an approximate concentration of 1 × 10^6^ cells/mL, the cells were collected and washed three times with PBS buffer. The rats were anesthetized by intraperitoneal injection of 100 µL of 3% pentobarbital sodium injection and then fixed on the surgical table after complete anesthesia, and the abdominal hair was removed with hair removal cream. An opening of about 1 cm in length was opened below the raphe with scissors, and the left outer lobe of the rat liver was plucked out of the body with a sterile cotton swab, then about 5 × 10^6^ cells were aspirated with an insulin needle and slowly injected into the middle of the left outer lobe of the rat liver. The needle hole was pressed with a cotton swab to avoid the flow of N1S1 cells, and then the syringe was withdrawn. The liver was returned to the rat's abdominal cavity after making sure that the needle hole was no longer bleeding, and then the wound was sutured and completely sterilized before being placed in the rat cage for further rearing until the seed tumor grew. The rats were housed in a clean environment at the Experimental Animal Center, and after 8 days, MRI imaging was performed on a 9.4 T small animal magnetic resonance imaging device to monitor the size of the liver tumors in situ in the rats.

The rat in situ liver cancer model treatment experiment was divided into 4 groups (saline group, free ^177^LuCl_3_ group, PID‐Ms group, and ^177^Lu‐PID‐Ms group), with 4 rats in each group. After the rats were anesthetized, the abdominal cavity of the rats was opened, and the abdominal artery, hepatic artery, and gastroduodenal artery were gently picked out with cotton swabs and forceps, and each vessel was separated. Then, two ligatures were placed around the gastroduodenal artery, and the distal portion of the gastroduodenal artery was also ligated. A ligature was placed around the celiac artery to temporarily interrupt blood flow. The gastroduodenal artery is punctured upstream of the distal ligature using a homemade needle, and a catheter is placed into the hepatic artery. After drug injection, the proximal portion of the gastroduodenal artery (upstream of the puncture site) is ligated. The ligature around the celiac artery was then untied to restore blood flow to the hepatic artery. Rats in the control group were injected with 200 µL of saline and ^177^LuCl_3_ (18.5 MBq, diluted in saline); rats in the PID‐Ms group were injected with 200 µL of PID‐Ms (10 mg/mL, dispersed in saline). Rats in the ^177^Lu‐PID‐Ms group were injected with 200 µL of ^177^Lu‐PID‐Ms (10 mg/mL, 18.5 MBq, dispersed in saline). Subsequently, rats with hepatocellular carcinoma in situ were diagnostically evaluated by nanoScan‐SPECT/CT scanner and 9.4 T animal MRI scanner to assess the treatment effect. In addition, the tumor size was estimated by the maximum (L) and minimum (S) diameters using the formula: Tumor volume/mm^3^ = (L × S^2^)/2. All experimental rats were euthanized after 14 days of treatment. Heart, normal liver, spleen, lung, and kidney tissues were collected separately and immediately immersed in 4% paraformaldehyde for H&E‐stained sections. Among them, the experimental rats in the free ^177^LuCl_3_ group and ^177^Lu‐PID‐Ms group were euthanized, and then the heart, liver, spleen, lung, kidney, blood, intestine, bone, skin, muscle, and stomach were taken for gamma counter test to obtain the results of ^177^LuCl_3_ and ^177^Lu‐PID‐Ms in vivo biodistribution.

### 4.18 TARE Treatment of Rabbits Orthotopic Live Tumor Model

The experiments were performed with the permission of the Xiamen University Animal Care and Use Committee and approved by the Xiamen University Animal Experimentation Ethics Committee (No. XMULAC20210102). For the orthotopic liver VX2 (RRID:CVCL_3864) tumor‐bearing rabbit, New Zealand white rabbits (2.5–3.0 kg) were obtained from Shanghai SLAC Laboratory Animal. VX2 tumor mass (Shanghai Lalan Biotechnology, Shanghai, China) was grafted on the hind leg of the rabbit. After 14 days, the VX2 tumor was obtained and cut into 1 mm^3^ piece to implant into the left liver lobe of the rabbit under abdominal ultrasound for orthotopic models. The tumors were confirmed using a 3.0T magnetic resonance imaging (MRI Magnetom Skyra scanner (Siemens Healthcare GmbH, Erlangen, Germany)). The TARE procedure of the rabbits orthotopic live tumor model was as follows. The orthotopic liver VX2 tumor‐bearing rabbit TARE treatment experiment was divided into four groups (saline group, free ^177^LuCl_3_ group, PID‐Ms group, and ^177^Lu‐PID‐Ms group), with three rabbits in each group. The orthotopic liver VX2 tumor‐bearing rabbit were anesthetized by intravenous injection of pentobarbital through the ear margins of the rabbits, the femoral artery was isolated and exposed, and after puncturing the artery with an 18‐gauge puncture needle, a microcatheter was fed into the artery through a vascular sheath. Then, under the real‐time guidance of DSA, the 2.1F microcatheter, and coaxial microguide wire were inserted through the 5F vascular sheath and selectively advanced to the nearest blood supply artery to the tumor. Then, iohexol was injected for hepatic arteriography to localize the orthotopic liver VX2 tumor‐bearing rabbit. Subsequently, saline, free ^177^LuCl_3_ (29.6 MBq), PID‐Ms (10 mg/mL), and ^177^Lu‐PID‐Ms (29.6 MBq) were pushed into the tumor lesions of rabbits in each group through the microcatheter at a uniform and slow rate to avoid reflux, respectively. After administration, iohexol was injected under DSA subtraction mode to detect the deposition of radioactive microspheres in the tumor vascular bed. The microcatheter was withdrawn and the wound was sutured, and antibiotic ampicillin was administered intramuscularly to the experimental rabbits at 3 days postoperatively to prevent wound infection and inflammation. After the 5th and 10th day of TARE treatment, each orthotopic liver VX2 tumor‐bearing rabbit was subjected to SPECT/CT imaging in a prone position to monitor the distribution of ^177^Lu‐PID‐Ms in vivo and the shunting of normal tissues and organs. Meanwhile, the tumor size of experimental rabbits at day 5 and day 10 of TARE treatment was monitored diagnostically using diffusion‐weighted imaging (DWI) and gadolinium contrast‐enhanced T1‐weighted images (T1WI‐Gd) sequences of 3.0 T MRI, and subsequently, after rabbits were euthanized 10 d after TARE treatment, tumors and major organs such as heart, liver, spleen, lungs, and kidneys were collected and treated with PBS They were rinsed clean and immediately fixed with 4.0% paraformaldehyde, followed by H&E staining and Ki‐67 (RRID:AB_302459) and γ‐H2AX (RRID:AB_420030) immunofluorescence staining, and scanned and photographed with fully automated section microscopy and high‐sensitivity laser confocal microscopy, respectively, to analyze the therapeutic mechanism of ^177^Lu‐PID‐Ms on the orthotopic liver VX2 tumor‐bearing rabbit. In addition, experimental rabbits in the free ^177^LuCl3 group and ^177^Lu‐PID‐Ms group were euthanized and tumors, heart, liver, spleen, lung, kidney, blood, intestine, bone, skin, muscle, and stomach were taken for γ‐counter testing to obtain in vivo biodistribution results of ^177^LuCl_3_ and ^177^Lu‐PID‐Ms. Throughout the study, tumor volumes were monitored and remained within the limits recommended by the ARRIVE guidelines.

### 4.19 Animal Welfare Monitoring

Throughout the study, all animals, including rats, rabbits, and pigs, were monitored at least twice daily by trained personnel in accordance with institutional animal care and use guidelines. Monitoring included assessment of general health status and clinical signs of distress, such as activity level, posture, gait, grooming behavior, food and water intake, responsiveness to external stimuli, and indicators of pain or discomfort (e.g., piloerection, lethargy, or abnormal movement). Humane endpoints were predefined prior to study initiation and included excessive tumor burden, severe deterioration in general condition, persistent inability to access food or water, or other signs of significant distress. For rodent and rabbit models, an additional criterion of body weight loss exceeding 15% of baseline was applied. Animals reaching any humane endpoint criteria would have been immediately euthanized in accordance with institutional ethical regulations. No animals reached the predefined humane endpoints before the planned study endpoint.

### 4.20 AI Tool and Graphic Copyright Disclosures

The authors declare that no generative AI tools were used in the preparation of this manuscript.

### 4.21 Statistical Analysis

All the results in this work were presented as mean values ± SD. Statistical analyses were performed with GraphPad Prism 8.3 software. Statistical significances were calculated via Student's t‐test or Mann−Whitney U test. *p < 0.05, **p < 0.01, and ***p < 0.001.

## Author Contributions

X.X., H.M., and G.L., conceived the idea and designed the research. X.X., Z.Z., Y.W., H.M., and G.L., designed the experiments. X.X., Z.Z., C.M., Y.W., and H.M performed the experiments. X.X., Z.Z., and L.X. analyzed the data and co‐wrote the paper. X.X., G.L., and H.M. supervised the project. All authors discussed the results and their implications and revised the manuscript at all stages.

## Conflicts of Interest

The authors declare no conflicts of interest.

## Supporting information




**Supporting File**: advs76278‐sup‐0001‐SuppMat.docx

## Data Availability

The data that supports the findings of this study are available in the supplementary material of this article.

## References

[1] F. Bray , M. Laversanne , H. Sung , et al., “Global Cancer Statistics 2022: GLOBOCAN Estimates of Incidence and Mortality Worldwide for 36 Cancers in 185 Countries,” CA: A Cancer Journal for Clinicians 74 (2024): 229–263. 38572751 10.3322/caac.21834

[2] H. Zhu , M. L. K. Chua , I. Chitapanarux , et al., “Global Radiotherapy Demands and Corresponding Radiotherapy‐Professional Workforce Requirements in 2022 and Predicted to 2050: A Population‐Based Study,” The Lancet Global Health 12 (2024): e1945–e1953. 39401508 10.1016/S2214-109X(24)00355-3

[3] H. Westerveld , N. Nesvacil , L. Fokdal , et al., “Definitive Radiotherapy With Image‐Guided Adaptive Brachytherapy for Primary Vaginal Cancer,” The Lancet Oncology 21 (2020): e157–e167. 32135119 10.1016/S1470-2045(19)30855-1

[4] T. I. Kostelnik and C. Orvig , “Radioactive Main Group and Rare Earth Metals for Imaging and Therapy,” Chemical Reviews 119 (2019): 902–956. 30379537 10.1021/acs.chemrev.8b00294

[5] E. Boros and A. B. Packard , “Radioactive Transition Metals for Imaging and Therapy,” Chemical Reviews 119 (2019): 870–901. 30299088 10.1021/acs.chemrev.8b00281

[6] P. J. Gawne , F. Man , P. J. Blower , and T. M. D. Rosales , “Direct Cell Radiolabeling for in Vivo Cell Tracking With PET and SPECT Imaging,” Chemical Reviews 122 (2022): 10266–10318. 35549242 10.1021/acs.chemrev.1c00767 PMC9185691

[7] I. Carbo‐Bague , C. Li , B. L. McNeil , et al., “Comparative Study of a Decadentate Acyclic Chelate, HOPO‐O_10_, and Its Octadentate Analogue, HOPO‐O_8_, for Radiopharmaceutical Applications,” Inorganic Chemistry 62 (2023): 20549–22056. 36608341 10.1021/acs.inorgchem.2c03671

[8] C. F. Ramogida and C. Orvig , “Tumour Targeting With Radiometals for Diagnosis and Therapy,” Chemical Communications 49 (2013): 4720–4739. 23599005 10.1039/c3cc41554f

[9] E. Horak , F. Hartmann , K. Garmestani , et al., “Radioimmunotherapy Targeting of HER2/Neu Oncoprotein on Ovarian Tumor Using Lead‐212‐DOTA‐AEl,” Journal of Nuclear Medicine 38 (1997): 1944. 9430475

[10] E. J. Rolleman , E. P. Krenning , B. F. Bernard , et al., “Long‐Term Toxicity of ^177^Lu‐DOTA^0^,Tyr^3^Octreotate in Rats,” European Journal of Nuclear Medicine and Molecular Imaging 34 (2007): 219–227. 17021812 10.1007/s00259-006-0232-1

[11] D. K. Cabbiness and D. W. Margerum , “Effect of Macrocyclic Structures on the Rate of Formation and Dissociation of Copper(II) Complexes,” Journal of the American Chemical Society 92 (1970): 2151–2153.

[12] M. S. Cooper , M. T. Ma , K. Sunassee , et al., “Comparison of ^64^Cu‐Complexing Bifunctional Chelators for Radioimmunoconjugation: Labeling Efficiency, Specific Activity, and in Vitro/in Vivo Stability,” Bioconjugate Chemistry 23 (2012): 1029–1039. 22471317 10.1021/bc300037w PMC4756438

[13] E. W. Price and C. Orvig , “Matching Chelators to Radiometals for Radiopharmaceuticals,” Chemical Society Reviews 43 (2014): 260–290. 24173525 10.1039/c3cs60304k

[14] G. J. Förster , M. Engelbach , J. Brockmann , et al., “Preliminary Data on Biodistribution and Dosimetry for Therapy Planning of Somatostatin Receptor Positive Tumours: Comparison of ^86^Y‐DOTATOC and ^111^In‐DTPA‐octreotide,” European Journal of Nuclear Medicine 28 (2001): 1743–1750. 11734910 10.1007/s002590100628

[15] L. Camera , S. Kinuya , K. Garmestani , et al., “Evaluation of the Serum Stability and in Vivo Biodistribution of CHX‐DTPA and Other Ligands for Yttrium Labeling of Monoclonal Antibodies,” Journal of Nuclear Medicine 35 (1994): 882. 8176477

[16] N. A. Thiele , V. Brown , J. M. Kelly , et al., “An Eighteen‐Membered Macrocyclic Ligand for Actinium‐225 Targeted Alpha Therapy,” Angewandte Chemie International Edition 56 (2017): 14712–14717. 28963750 10.1002/anie.201709532

[17] A. Hu , E. Aluicio‐Sarduy , V. Brown , et al., “Py‐Macrodipa: A Janus Chelator Capable of Binding Medicinally Relevant Rare‐Earth Radiometals of Disparate Sizes,” Journal of the American Chemical Society 143 (2021): 10429–10440. 34190542 10.1021/jacs.1c05339 PMC8491276

[18] A. Hu and J. J. Wilson , “Advancing Chelation Strategies for Large Metal Ions for Nuclear Medicine Applications,” Accounts of Chemical Research 55 (2022): 904–915. 35230803 10.1021/acs.accounts.2c00003 PMC9364011

[19] L. Wharton , E. Kurakina , V. Radchenko , P. Schaffer , and C. Orvig , “Chemical Promiscuity of Non‐Macrocyclic Multidentate Chelating Ligands for Radiometal Ions: H_4_neunpa‐NH_2_ vs H_4_noneunpa,” Inorganic Chemistry 60 (2021): 4076–4092. 33635057 10.1021/acs.inorgchem.1c00152

[20] L. Li , J. Rousseau , M. D. G. Jaraquemada‐Peláez , et al., “ ^225^Ac‐H4py4pa for Targeted Alpha Therapy,” Bioconjugate Chemistry 32 (2021): 1348–1363. 32216377 10.1021/acs.bioconjchem.0c00171

[21] E. W. Price , J. F. Cawthray , G. A. Bailey , et al., “H4octapa: An Acyclic Chelator for ^111^In Radiopharmaceuticals,” Journal of the American Chemical Society 134 (2012): 8670–8683. 22540281 10.1021/ja3024725

[22] M. G. Jaraquemada‐Peláez , X. Wang , T. J. Clough , et al., “H4octapa: Synthesis, Solution Equilibria and Complexes With Useful Radiopharmaceutical Metal Ions,” Dalton Transactions 46 (2017): 14647–14658. 28853751 10.1039/c7dt02343j

[23] X. Wang , M. D. G. Jaraquemada‐Peláez , C. Rodríguez‐Rodríguez , et al., “H4octox: Versatile Bimodal Octadentate Acyclic Chelating Ligand for Medicinal Inorganic Chemistry,” Journal of the American Chemical Society 140 (2018): 15487–15500. 30394734 10.1021/jacs.8b09964

[24] N. H. Nicolay , D. P. Berry , and R. A. Sharma , “Liver Metastases From Colorectal Cancer: Radioembolization With Systemic Therapy,” Nature Reviews Clinical Oncology 6 (2009): 687–697. 10.1038/nrclinonc.2009.165 19884901

[25] E. O. Aboagye , T. D. Barwick , and U. Haberkorn , “Radiotheranostics in Oncology: Making Precision Medicine Possible,” CA: A Cancer Journal for Clinicians 73 (2023): 255–274. 36622841 10.3322/caac.21768

[26] J. E. Dancey , F. A. Shepherd , K. Paul , et al., “Treatment of Nonresectable Hepatocellular Carcinoma With Intrahepatic 90Y‐Microspheres,” Journal of Nuclear Medicine 41 (2000): 1673–1681. 11037997

[27] M. L. J. Smits , M. Elschot , M. A. A. J. van den Bosch , et al., “In Vivo Dosimetry Based on SPECT and MR Imaging of ^166^Ho‐Microspheres for Treatment of Liver Malignancies,” Journal of Nuclear Medicine 54 (2013): 2093–2100. 24136931 10.2967/jnumed.113.119768

[28] J. F. Prince , M. A. A. J. van den Bosch , J. F. W. Nijsen , et al., “Efficacy of Radioembolization With ^166^Ho‐Microspheres in Salvage Patients With Liver Metastases: A Phase 2 Study,” Journal of Nuclear Medicine 59 (2018): 582–588. 28916623 10.2967/jnumed.117.197194

[29] L. D. J. C. Vega , P. L. Esquinas , C. Rodríguez‐Rodríguez , et al., “Radioembolization of Hepatocellular Carcinoma With Built‐In Dosimetry: First in Vivo Results With Uniformly‐Sized, Biodegradable Microspheres Labeled With ^188^Re,” Theranostics 9 (2019): 868–883. 30809314 10.7150/thno.29381 PMC6376476

[30] T. Lu and Q. Chen , “Independent Gradient Model Based on Hirshfeld Partition: A New Method for Visual Study of Interactions in Chemical Systems,” Journal of Computational Chemistry 43 (2022): 539–555. 35108407 10.1002/jcc.26812

[31] R. G. Selwyn , M. A. Avila‐Rodriguez , A. K. Converse , et al., “ ^18^F‐Labeled Resin Microspheres as Surrogates for ^90^Y Resin Microspheres Used in the Treatment of Hepatic Tumors: A Radiolabeling and PET Validation Study,” Physics in Medicine and Biology 52 (2007): 7397–7408. 18065846 10.1088/0031-9155/52/24/013

[32] S. Ho , W. Y. Lau , T. W. T. Leung , M. Chan , P. J. Johnson , and A. K. C. Li , “Clinical Evaluation of the Partition Model for Estimating Radiation Doses From Yttrium‐90 Microspheres in the Treatment of Hepatic Cancer,” European Journal of Nuclear Medicine 24 (1997): 293–298. 9143467 10.1007/BF01728766

[33] W. Bult , H. de Leeuw , O. M. Steinebach , et al., “Radioactive Holmium Acetylacetonate Microspheres for Interstitial Microbrachytherapy: An in Vitro and in Vivo Stability Study,” Pharmaceutical Research 29 (2012): 827–836. 22068276 10.1007/s11095-011-0610-7 PMC3281200

[34] W. Bult , M. A. D. Vente , E. Vandermeulen , et al., “Microbrachytherapy Using Holmium‐166 Acetylacetonate Microspheres: A Pilot Study in a Spontaneous Cancer Animal Model,” Brachytherapy 12, no. 2441 (2013): 171–177. 22999975 10.1016/j.brachy.2012.08.001

[35] R. C. Bakker , M. G. E. H. Lam , S. A. van Nimwegen , A. J. W. P. Rosenberg , R. J. J. van Es , and J. F. W. Nijsen , “Intratumoral Treatment With Radioactive Beta‐Emitting Microparticles: A Systematic Review,” Journal of Radiation Oncology 6 (2017): 323–341. 29213358 10.1007/s13566-017-0315-6 PMC5700992

[36] S. A. van Nimwegen , R. C. Bakker , J. Kirpensteijn , et al., “Intratumoral Injection of Radioactive Holmium (^166^Ho) Microspheres for Treatment of Oral Squamous Cell Carcinoma in Cats,” Veterinary and Comparative Oncology 16 (2018): 114–124. 28480610 10.1111/vco.12319

[37] R. C. Bakker , R. J. J. van Es , A. J. W. P. Rosenberg , et al., “Intratumoral Injection of Radioactive Holmium‐166 Microspheres in Recurrent Head and Neck Squamous Cell Carcinoma,” Nuclear Medicine Communications 39 (2018): 213–221. 29309367 10.1097/MNM.0000000000000792 PMC5815636

[38] L. Bodei , K. Herrmann , H. Schoder , A. M. Scott , and J. S. Lewis , “Radiotheranostics in Oncology: Current Challenges and Emerging Opportunities,” Nature Reviews Clinical Oncology 19 (2022): 534–550. 10.1038/s41571-022-00652-y PMC10585450 35725926

[39] W. Song , X. Zhang , Y. Song , et al., “Enhancing Photothermal Therapy Efficacy by in Situ Self‐Assembly in Glioma,” ACS Applied Materials Interfaces 15 (2022): 57–66. 36206382 10.1021/acsami.2c14413 PMC9839507

[40] B. Morgan , A. S. Kennedy , V. Lewington , B. Jones , and R. A. Sharma , “Intra‐Arterial Brachytherapy of Hepatic Malignancies: Watch the Flow,” Nature Reviews Clinical Oncology 8 (2011): 115–120. 10.1038/nrclinonc.2010.153 20924355

[41] M. J. Frisch , G. W. Trucks , H. B. Schlegel , et al., “aussian 09, Revision A.02; Gaussian, Inc.: Wallingford, CT,” (2009).

[42] C. Lee , W. Yang , and R. G. Parr , “Development of the Colle‐Salvetti Correlation‐Energy Formula Into a Functional of the Electron Density,” Physical Review B 37 (1988): 785–789. 10.1103/physrevb.37.785 9944570

[43] T. Lu and F. Chen , “Multiwfn: A Multifunctional Wavefunction Analyzer,” Journal of Computational Chemistry 33 (2012): 580–592. 22162017 10.1002/jcc.22885

[44] W. Humphrey , A. Dalke , and K. Schulten , “VMD: Visual Molecular Dynamics,” Journal of Molecular Graphics 14 (1996): 33–38. 8744570 10.1016/0263-7855(96)00018-5

