# Supplementary material for: Ultra‐Radiostable Covalent Conformationally Interlocked Networks Enabling a Universal Radiometal‐Labeling Platform for Cancer Radioembolization
Source: Adv Sci (Weinh). 2026 Jun 23:e76278. Online ahead of print. doi: 10.1002/advs.76278 (PMC13336425; doi:10.1002/advs.76278)
Supplement: Supplementary file 1 — Supporting File: advs76278‐sup‐0001‐SuppMat.docx [file ADVS-9999-e76278-s001.docx]

**Supporting Information for**

**Ultra-radiostable covalent conformationally interlocked networks enabling a universal radiometal-labeling platform for cancer radioembolization**

Xiao Xu^1,^*, Zhenwen Zhao^2,#^, Yangjie Wang^3,#^, Zhigang Liu^1^, Zhijie Liu^1^, Xiaoling Li^1^, Zhichen Mao^1,4^, Lu Xu^5^, Gan Lin^2^, Gang Liu^2,^*, Hongjuan Ma^4,^*

^1^ Cancer Center, Guangdong Engineering Research Center of Boron Neutron Therapy and Application in Malignant Tumors, The Tenth Affiliated Hospital, Southern Medical University (Dongguan People's Hospital), Southern Medical University, Dongguan 523059, China.

^2^ State Key Laboratory of Vaccines for Infectious Diseases, Xiang An Biomedicine Laboratory, National Innovation Platform for Industry-Education Integration in Vaccine Research, School of Public Health, Xiamen University, Xiamen, 361102, China

^3^ Shanghai Advanced Research Institute, Chinese Academy of Sciences, Shanghai 201210, China

^4^ Shanghai Applied Radiation Institute, School of Environmental and Chemical Engineering, Shanghai University, Shanghai 200444, China

^5^ Shanghai Institute of Applied Physics, Chinese Academy of Sciences, Shanghai, 201800, China

*Corresponding authors: Xiao Xu (xiaoxu721@smu.edu.cn), Gang Liu (gangliu.cmitm@xmu.edu.cn), and Hongjuan Ma (hongjuanma@shu.edu.cn)


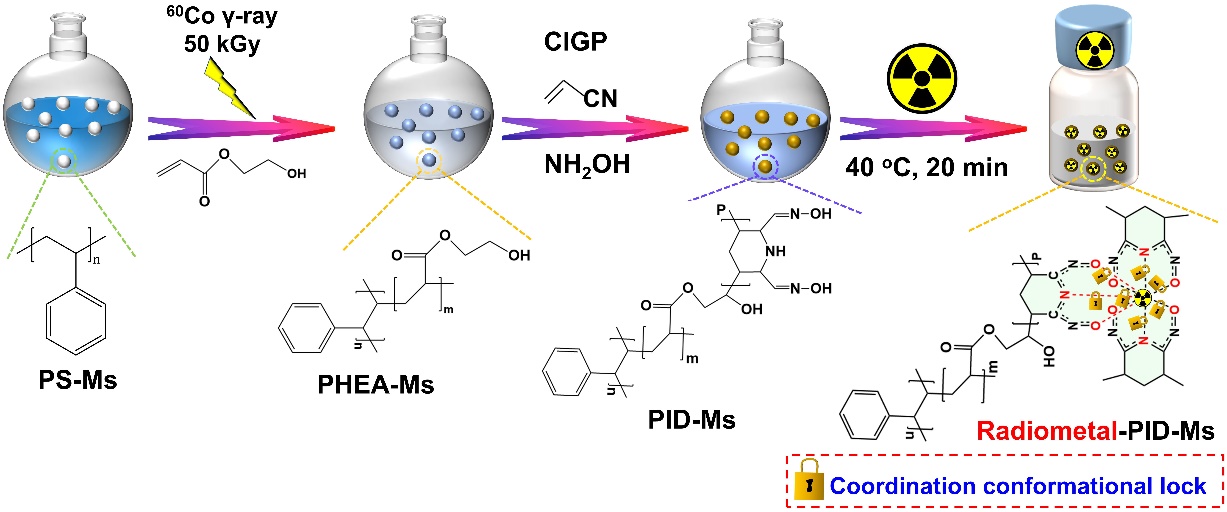


**Fig. S1 Schematic diagram of the radiosynthesis of radiometal-PID-Ms.** Chemical structural evolution of grafted chains in PID-Ms induced by ^60^Co radiation graft polymerization combined with Ce^4+^ initiated graft polymerization. Subsequently, Radiometal labeled PID-Ms were obtained as radiometal-PID-Ms.


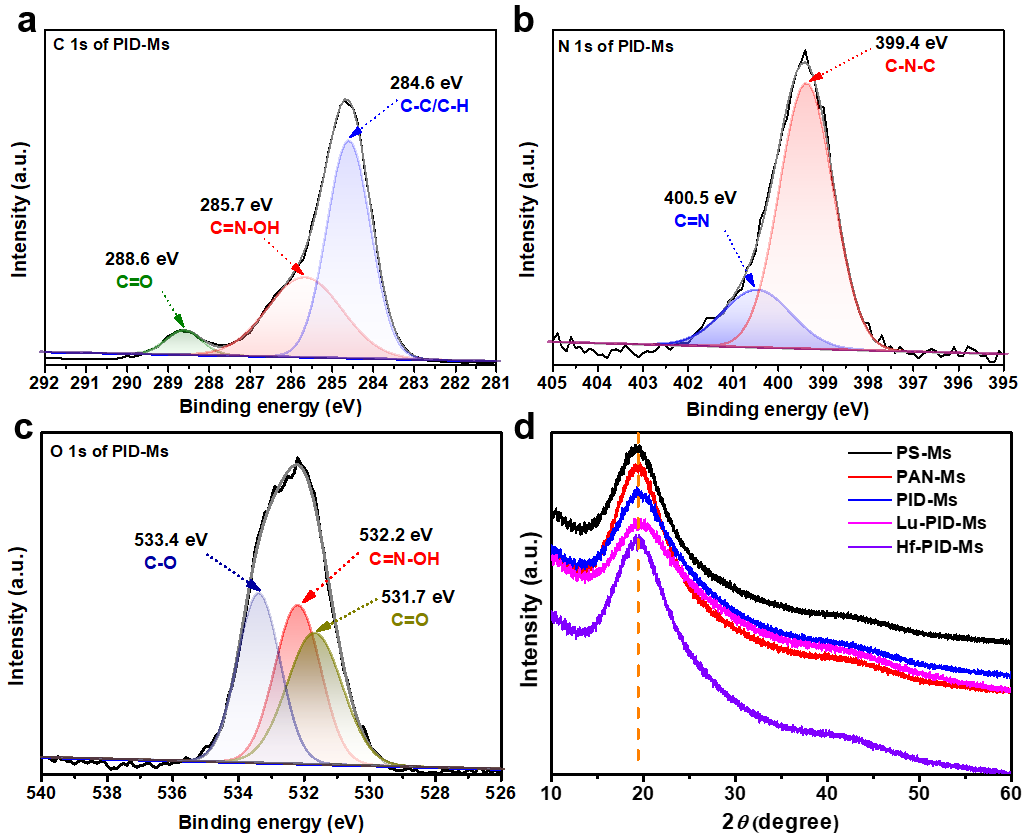


**Fig. S2** High-resolution XPS of (**c**) C 1s, (**d**) N 1s, and (**e**) O 1s for PID-Ms, respectively. (**d**) XRD patterns of the PS-Ms, PAN-Ms, PID-Ms, Lu-PID-Ms, and Hf-PID-Ms, respectively.


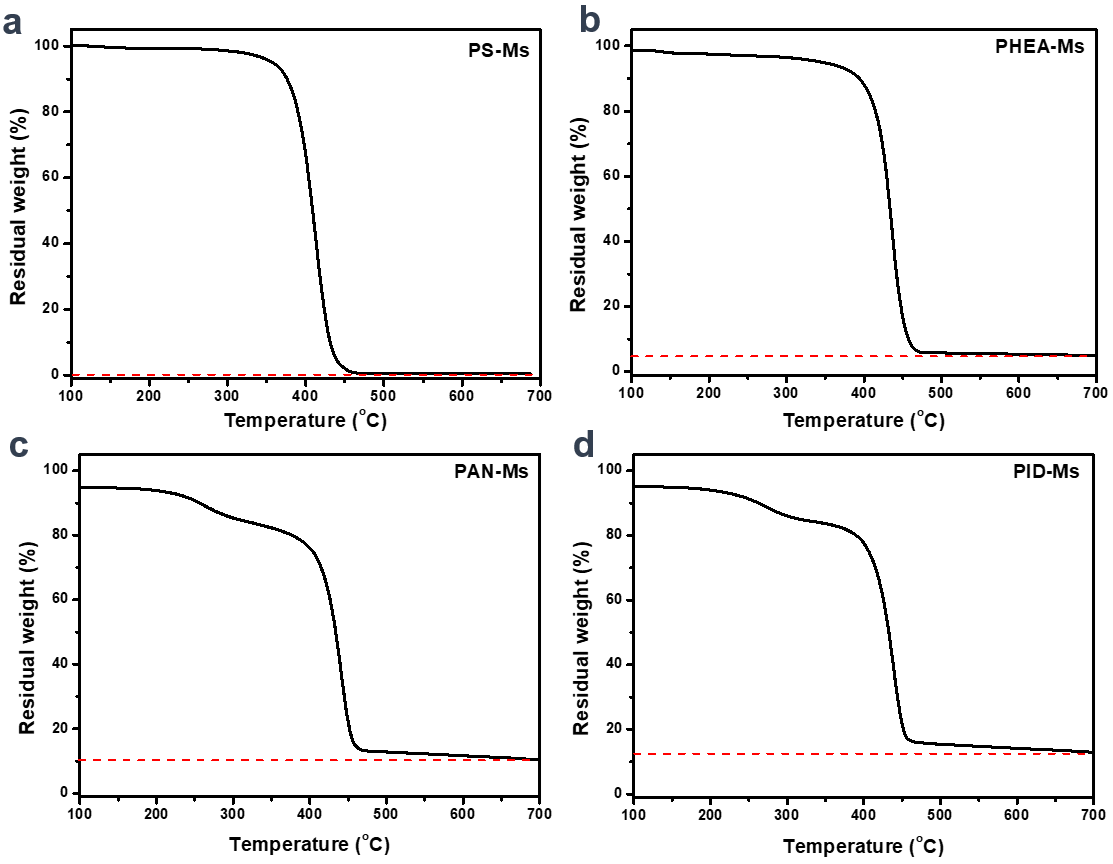


**Fig. S3** TGA curves of PS-Ms (**a**), PHEA-Ms (**b**), PAN-Ms (**c**), and PID-Ms (**d**) in an N_2_ atmosphere.


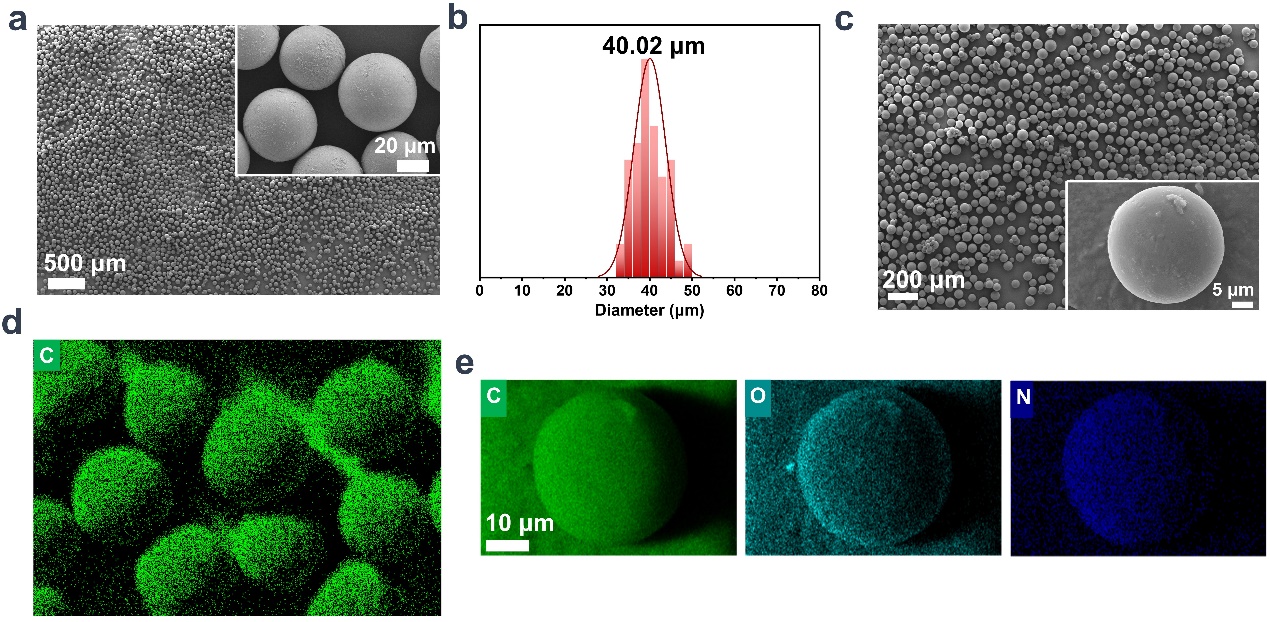


**Fig. S4 a** SEM images of PS-Ms. **b** Corresponding to diameter distribution of PS-Ms. **c** SEM images of PAN-Ms. **d** EDS mapping of PS-Ms. **e** EDS mapping of PAN-Ms.


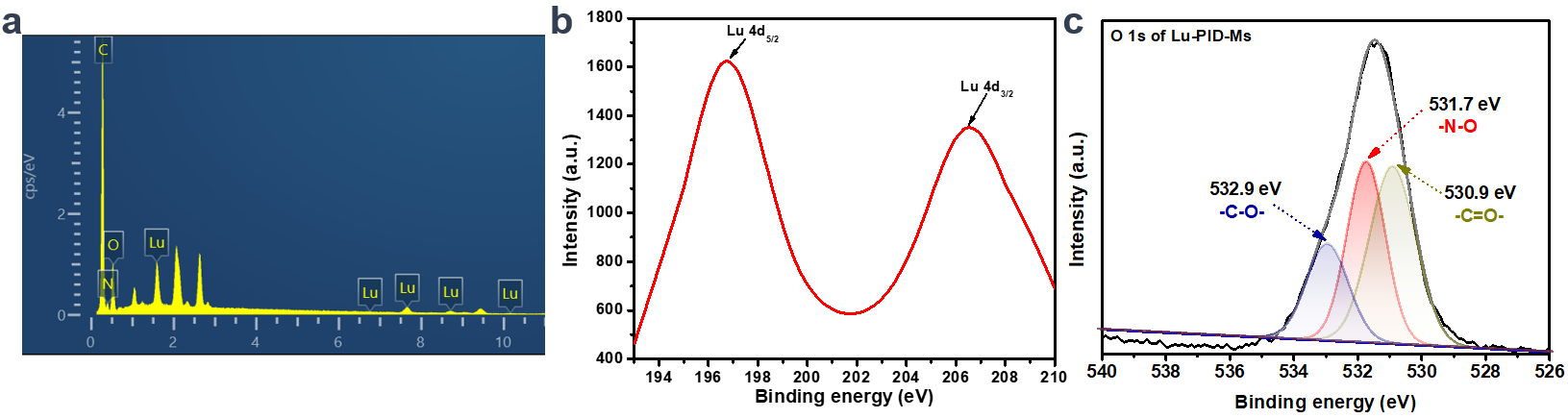
**Fig. S5 a**. EDX spectrum of Hf-PID-Ms. **b** XPS spectrum of Lu-PID-Ms. **c** High-resolution XPS of O 1s for Lu-PID-Ms.


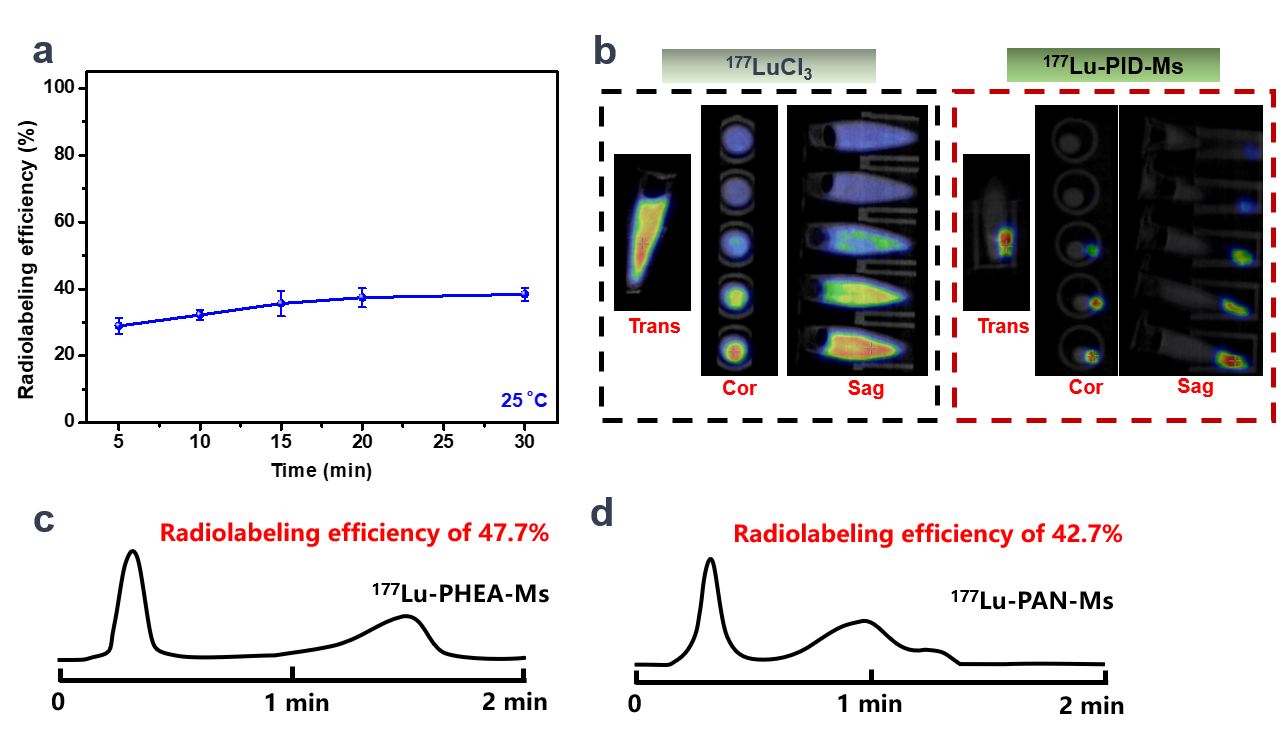


**Fig. S6 a** Radiolabeling efficiency of ^177^Lu-PID-Ms as a function of reaction time at 25^o^C. **b** Transaxial slice, coronal slice, and sagittal slice of ^177^LuCl_3_ and ^177^Lu-PID-Ms in saline. Radio-TLC analysis of (**c**) ^177^Lu-PHEA-Ms and (**d**) ^177^Lu-PAN-Ms.


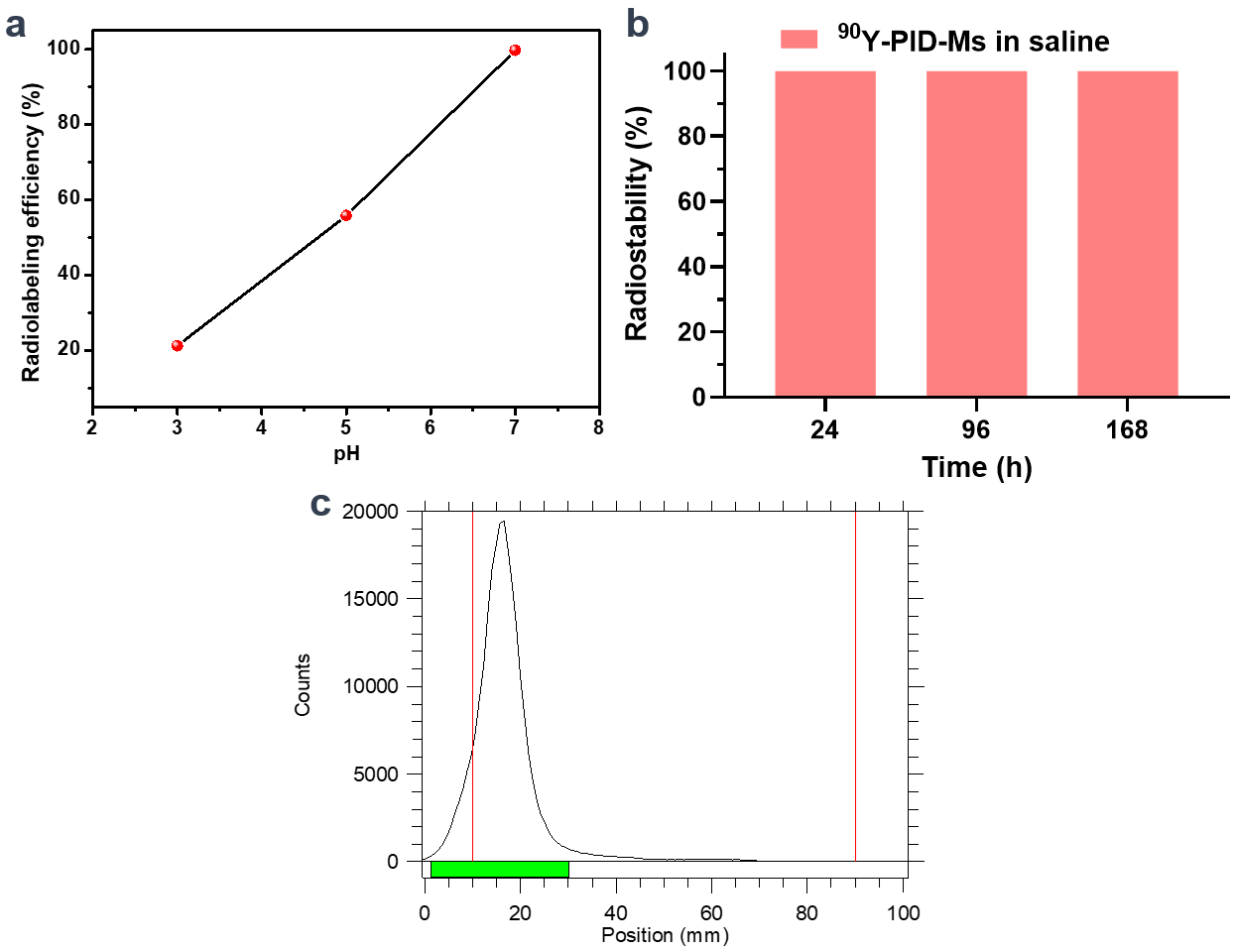


**Fig. S7 a** Radiolabeling efficiency of the ^90^Y-PID-Ms as a function of pH. **b** *In vitro* radiostability of the ^90^Y-PID-Ms in saline at different contacting periods. **c** Radio-TLC analysis of ^188^Re-PID-Ms.


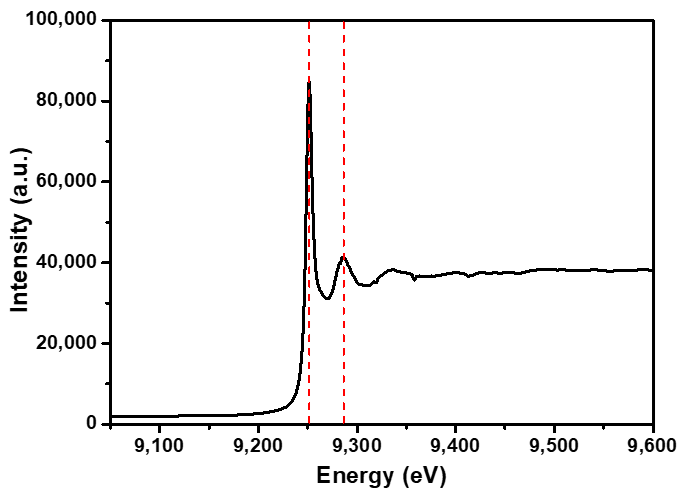


**Fig. S8** *L*_III_-edge EXAFS profiles of the Lu-PID-Ms.


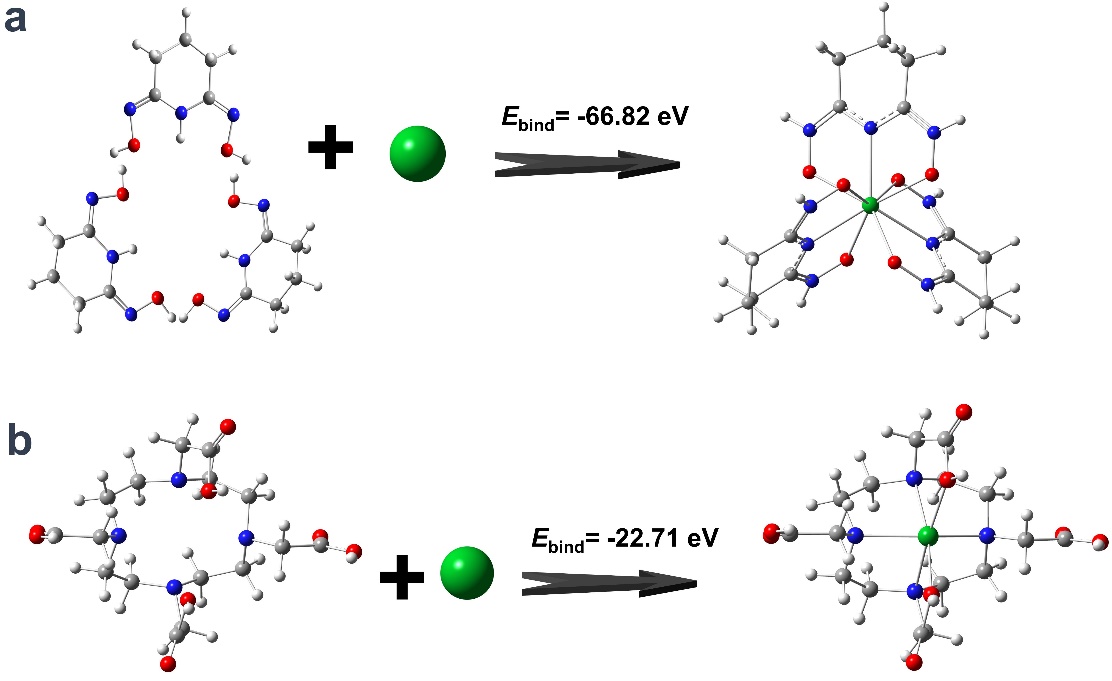


**Fig. S9** DFT calculation for the coordination complexation of Lu with PID ligand and DOTA ligand.


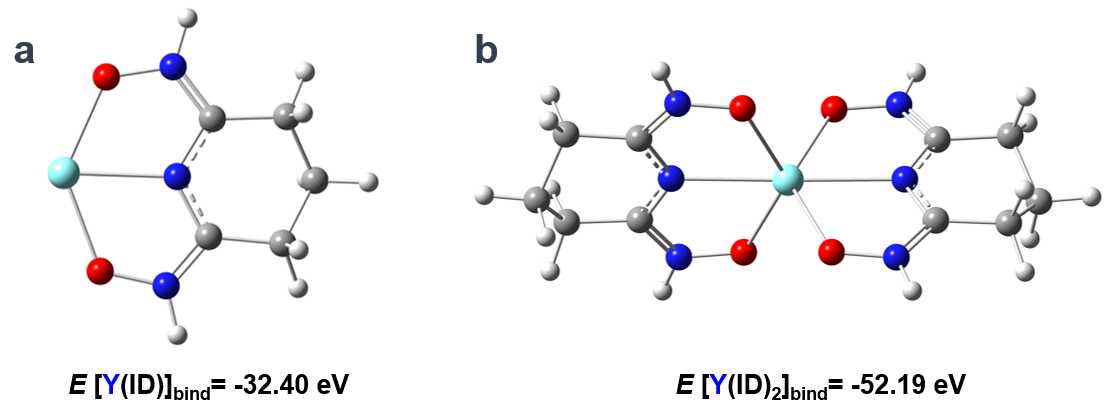


**Fig. S10** Coordination configuration and binding energy of (**a**) [Y(ID)] complex and (**b**) [Y(ID)_2_] complex .


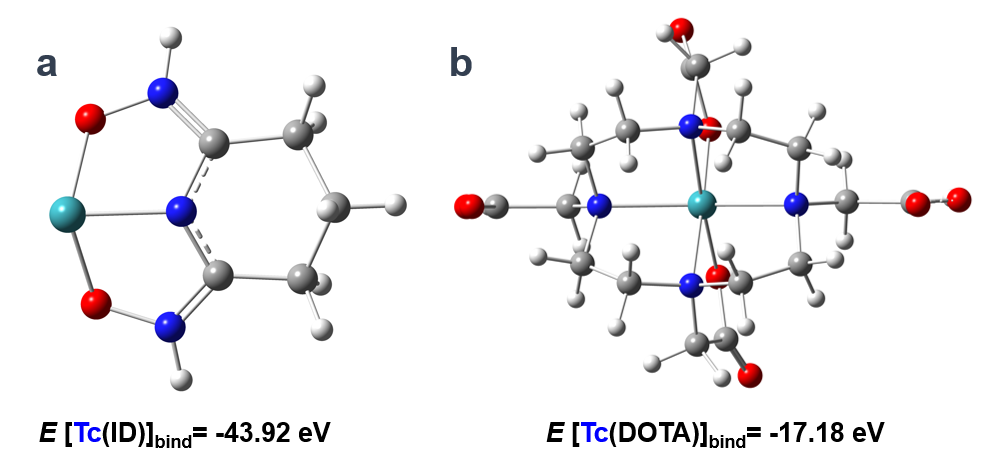


**Fig. S11** Coordination configuration and binding energy of (**a**) [Tc(ID)] complex and (**b**) [Tc(DOTA)] complex .


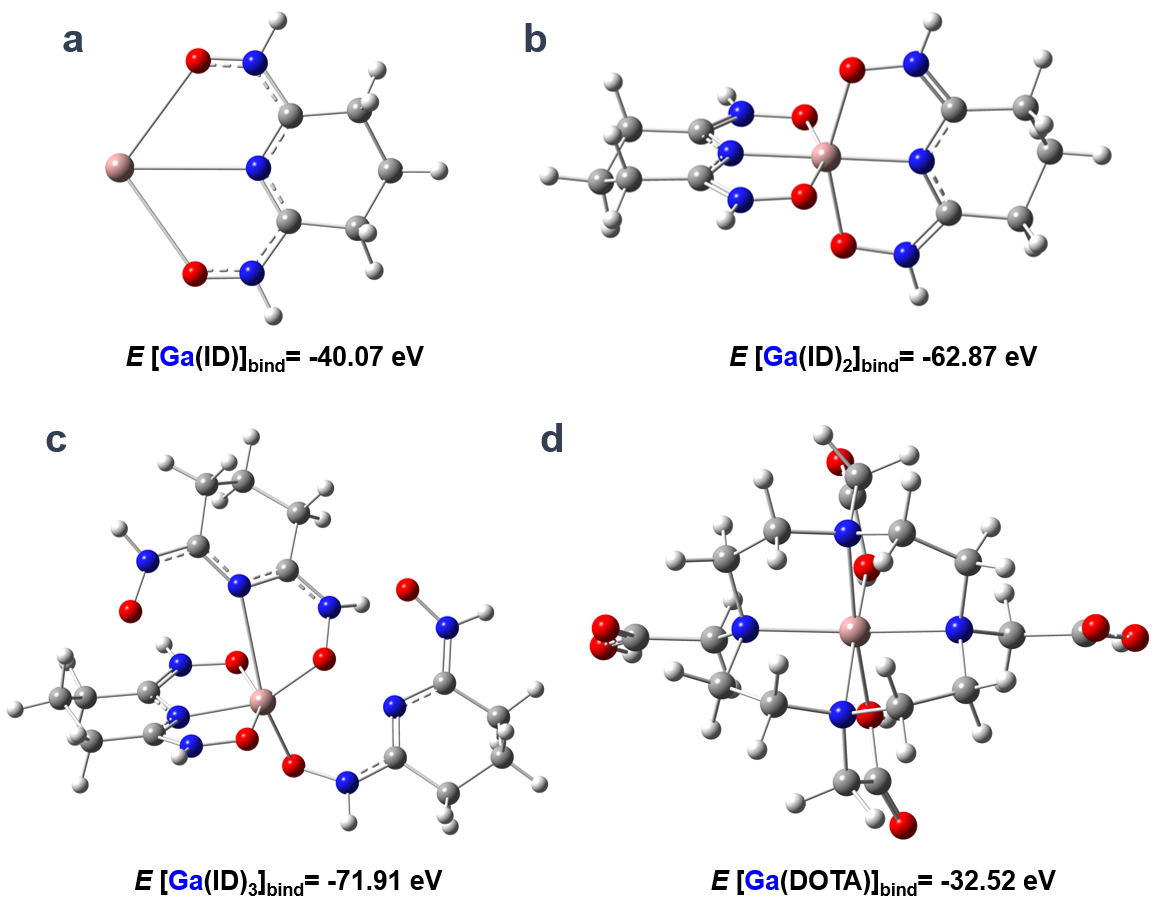


**Fig. S12** Coordination configuration and binding energy of (**a**) [Ga(ID)] complex, (**b**) [Ga(ID)_2_] complex, (**c**) [Ga(ID)_3_] complex, and (**d**) [Ga(DOTA)] complex.


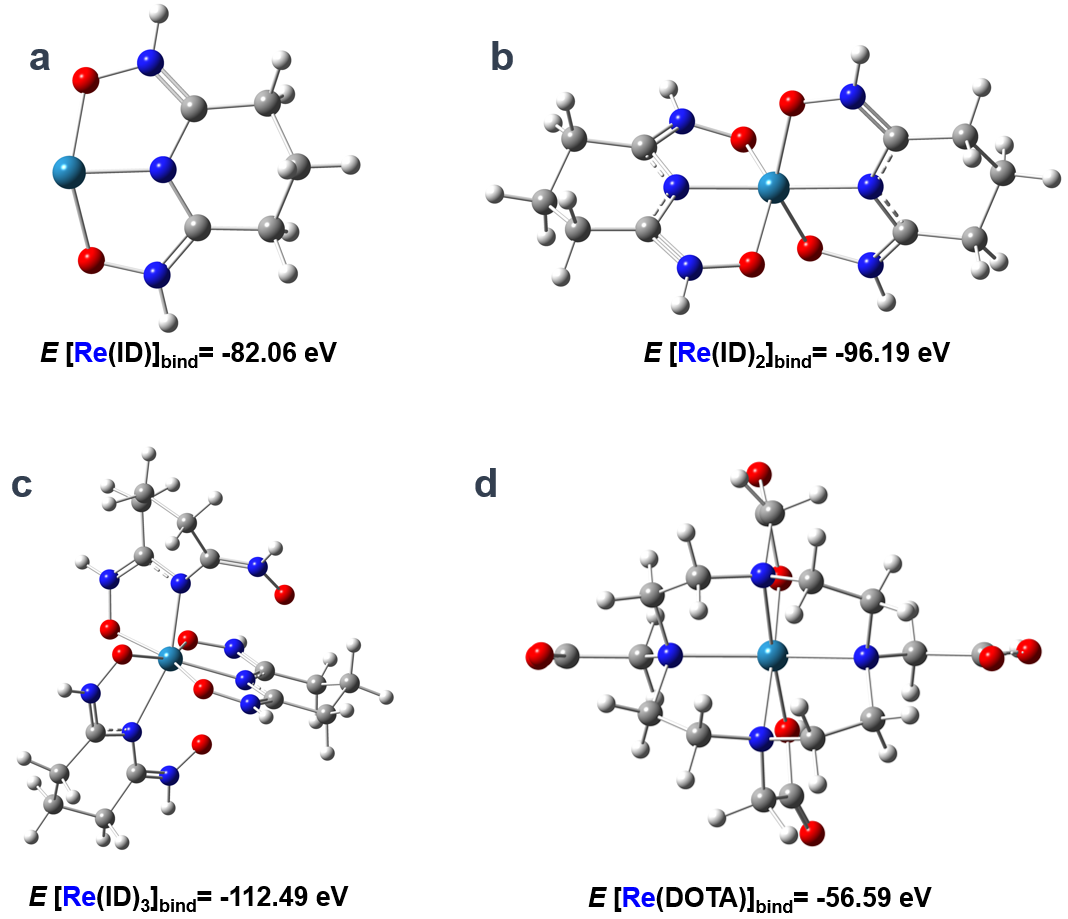


**Fig. S13** Coordination configuration and binding energy of (**a**) [Re(ID)] complex, (**b**) [Re(ID)_2_] complex, (**c**) [Re(ID)_3_] complex, and (**d**) [Re(DOTA)] complex.


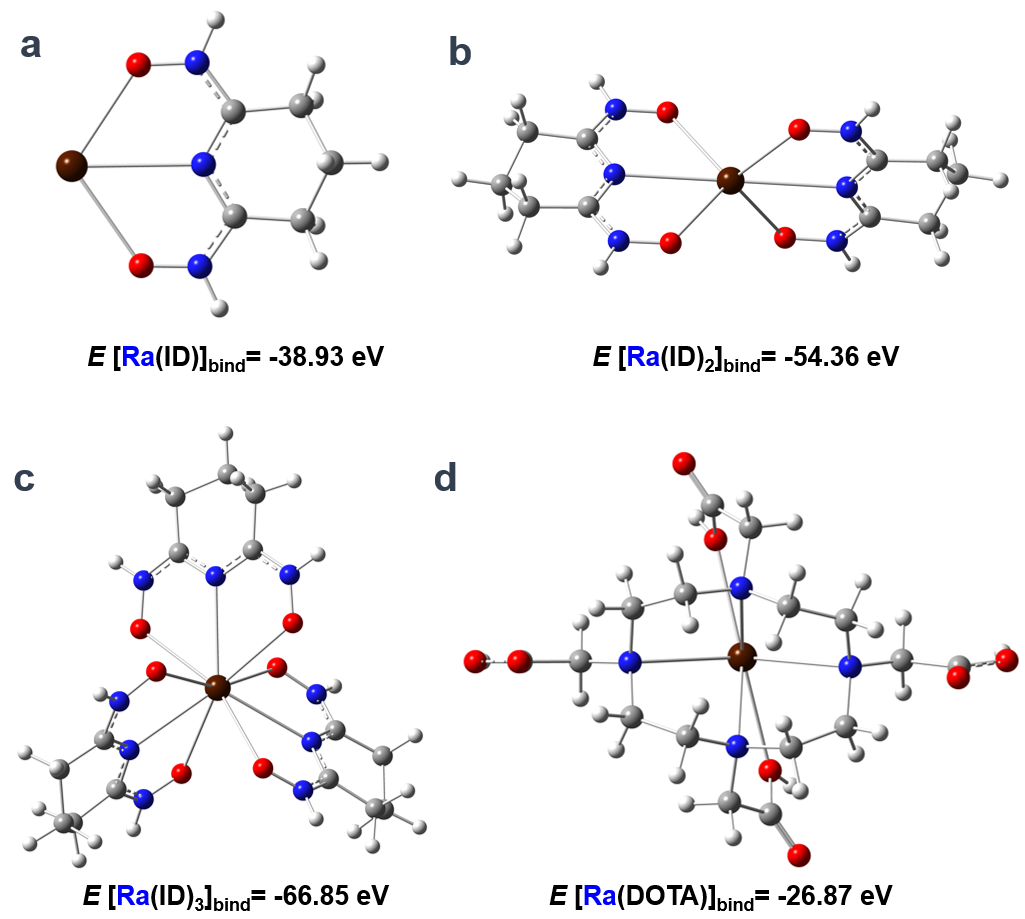


**Fig. S14** Coordination configuration and binding energy of (**a**) [Ra(ID)] complex, (**b**) [Ra(ID)_2_] complex, (**c**) [Ra(ID)_3_] complex, and (**d**) [Ra(DOTA)] complex.


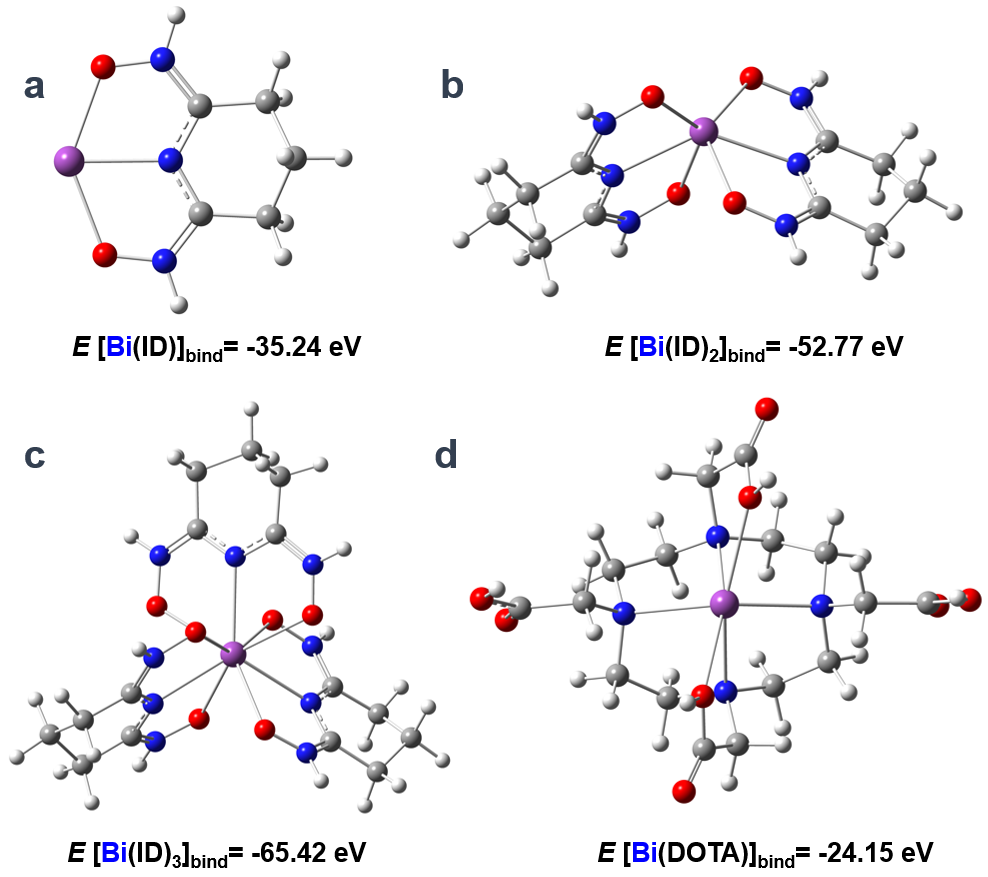


**Fig. S15** Coordination configuration and binding energy of (**a**) [Bi(ID)] complex, (**b**) [Bi(ID)_2_] complex, (**c**) [Bi(ID)_3_] complex, and (**d**) [Bi(DOTA)] complex.


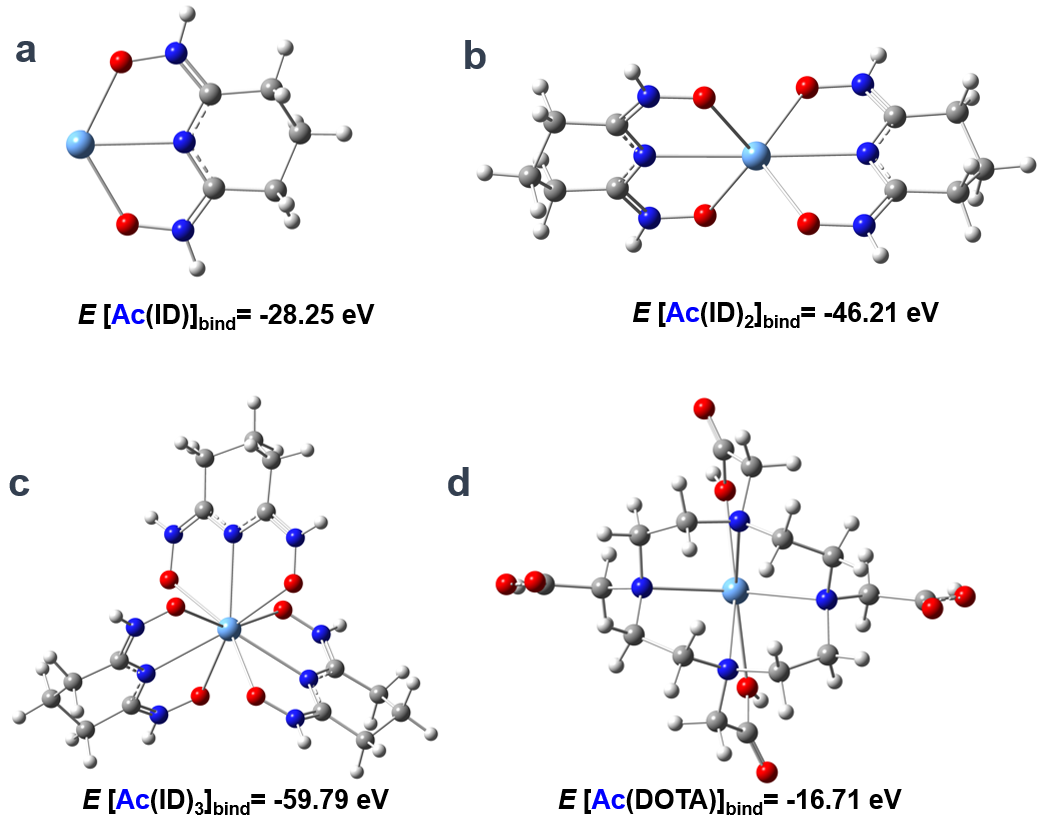
 **Fig. S16** Coordination configuration and binding energy of (**a**) [Ac(ID)] complex, (**b**) [Ac(ID)_2_] complex, (**c**) [Ac(ID)_3_] complex, and (**d**) [Ac(DOTA)] complex.


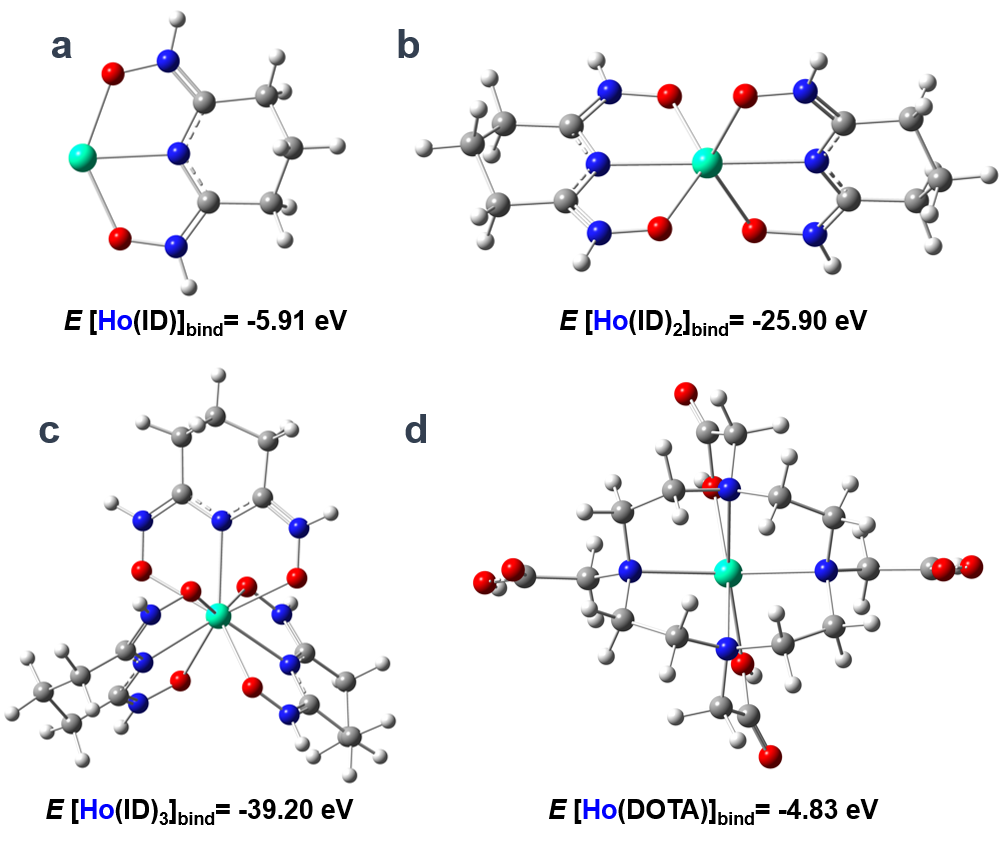


**Fig. S17** Coordination configuration and binding energy of (**a**) [Ho(ID)] complex, (**b**) [Ho(ID)_2_] complex, (**c**) [Ho(ID)_3_] complex, and (**d**) [Ho(DOTA)] complex.


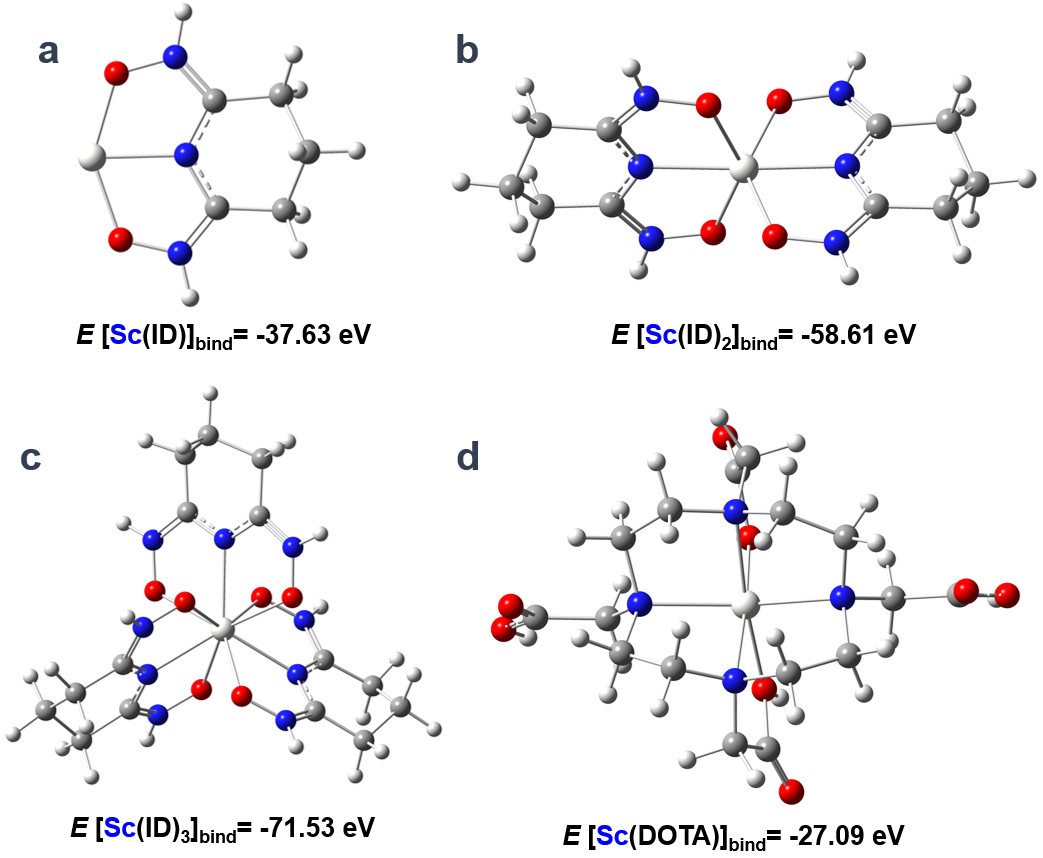


**Fig. S18** Coordination configuration and binding energy of (**a**) [Sc(ID)] complex, (**b**) [Sc(ID)_2_] complex, (**c**) [Sc(ID)_3_] complex, and (**d**) [Sc(DOTA)] complex.


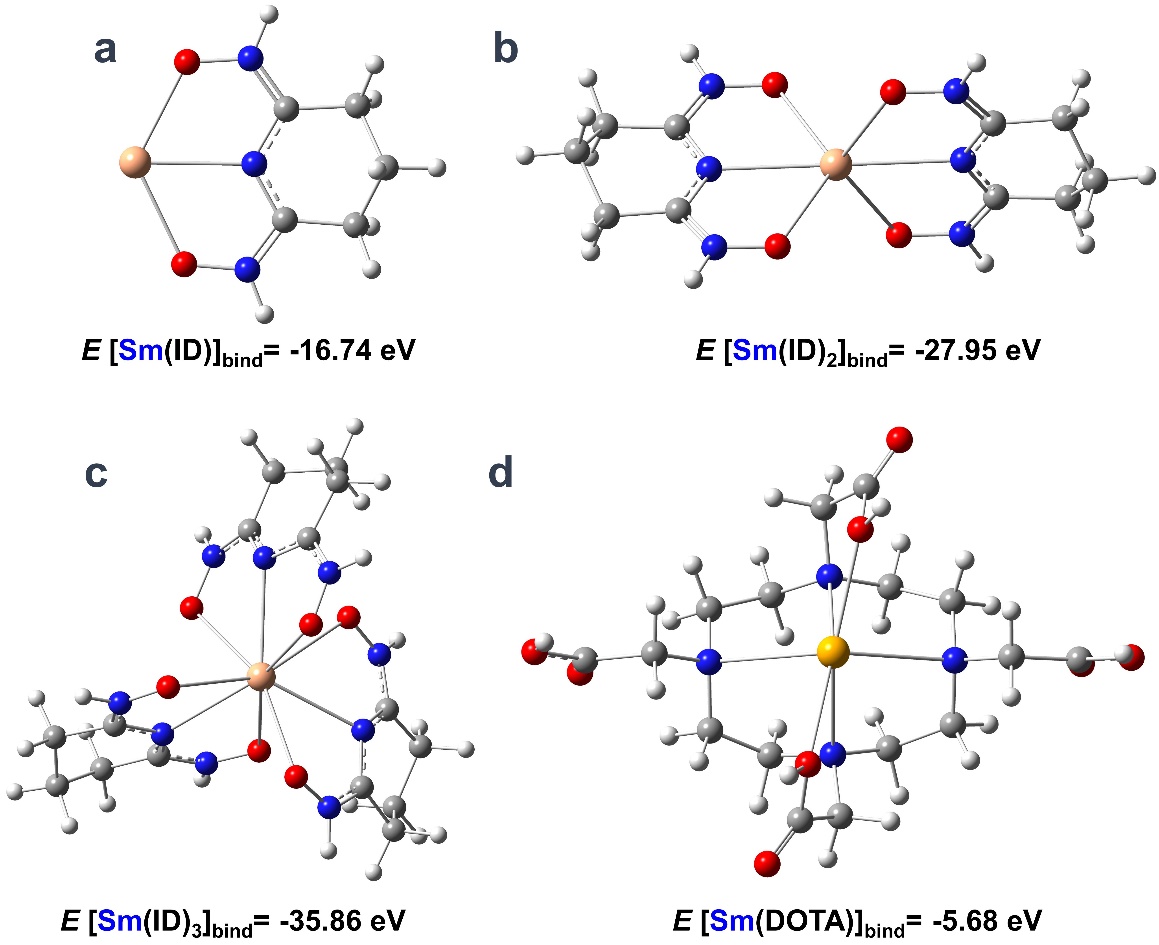


**Fig. S19** Coordination configuration and binding energy of (**a**) [Sm(ID)] complex, (**b**) [Sm(ID)_2_] complex, (**c**) [Sm(ID)_3_] complex, and (**d**) [Sm(DOTA)] complex.


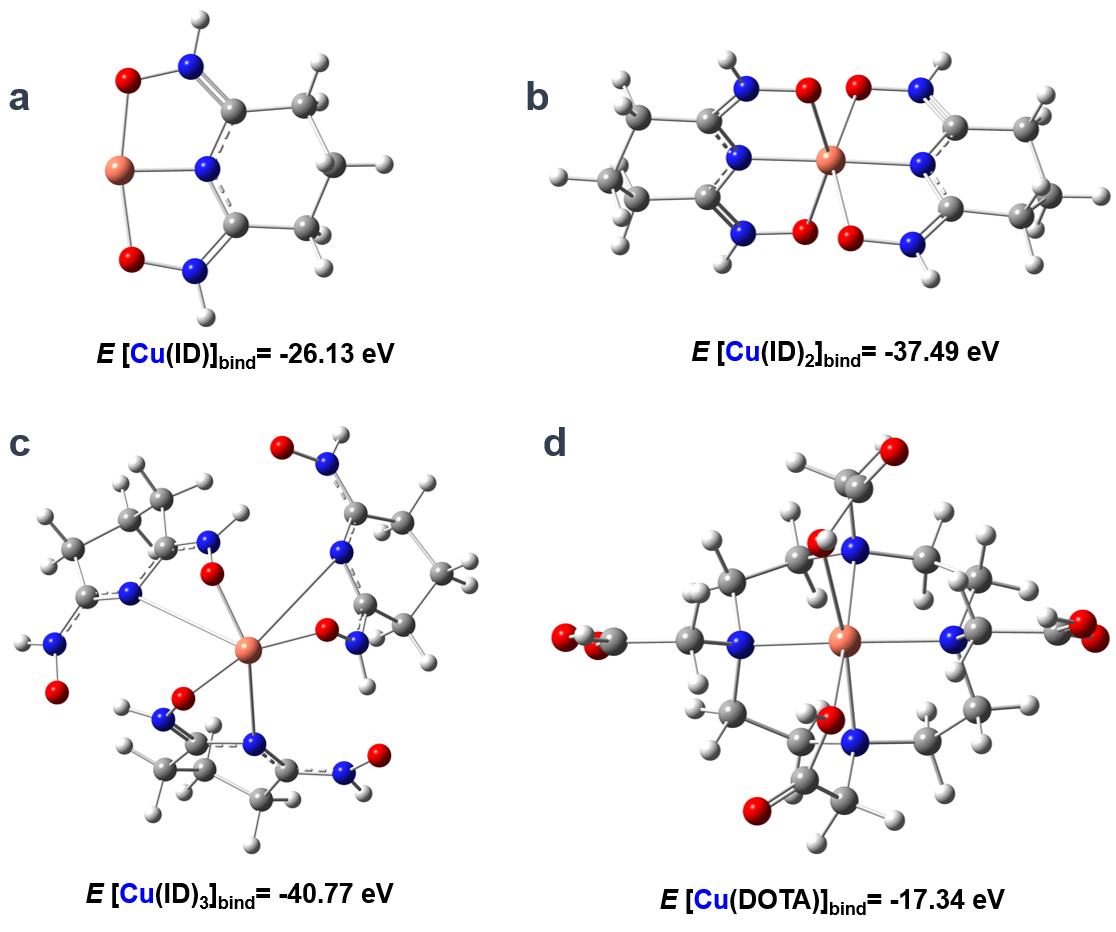


**Fig. S20** Coordination configuration and binding energy of (**a**) [Cu(ID)] complex, (**b**) [Cu(ID)_2_] complex, (**c**) [Cu(ID)_3_] complex, and (**d**) [Cu(DOTA)] complex.


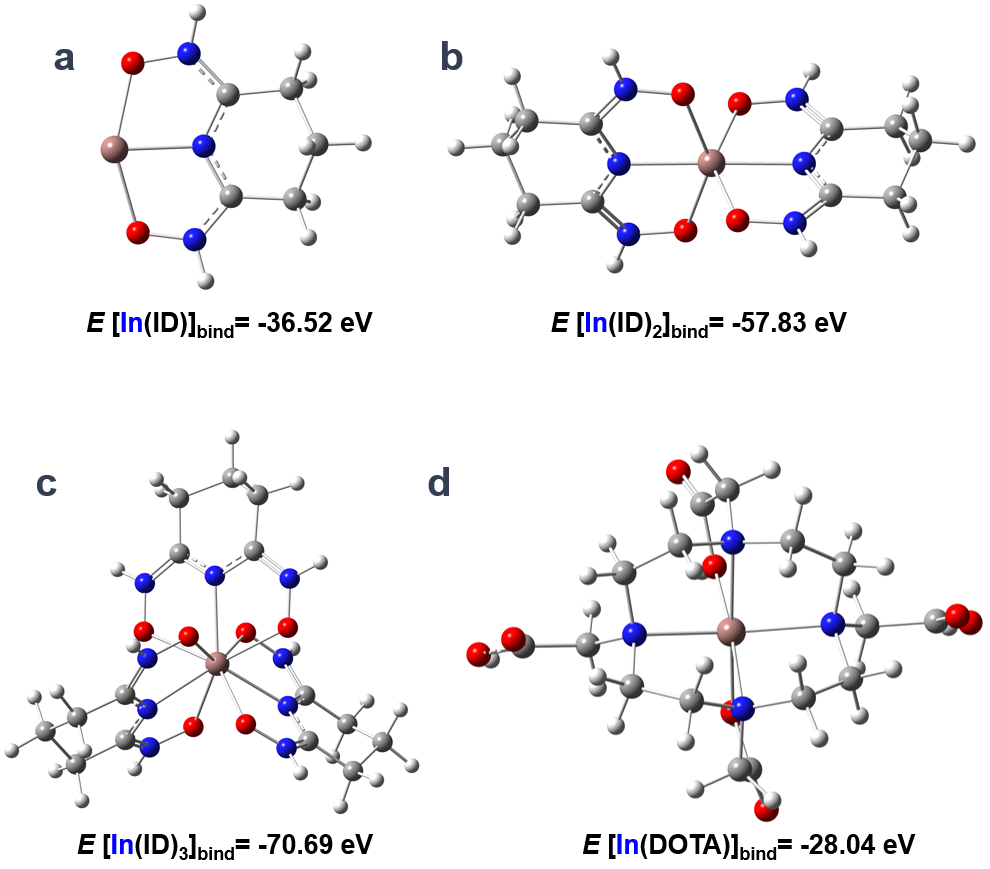
 **Fig. S21** Coordination configuration and binding energy of (**a**) [In(ID)] complex, (**b**) [In(ID)_2_] complex, (**c**) [In(ID)_3_] complex, and (**d**) [In(DOTA)] complex.


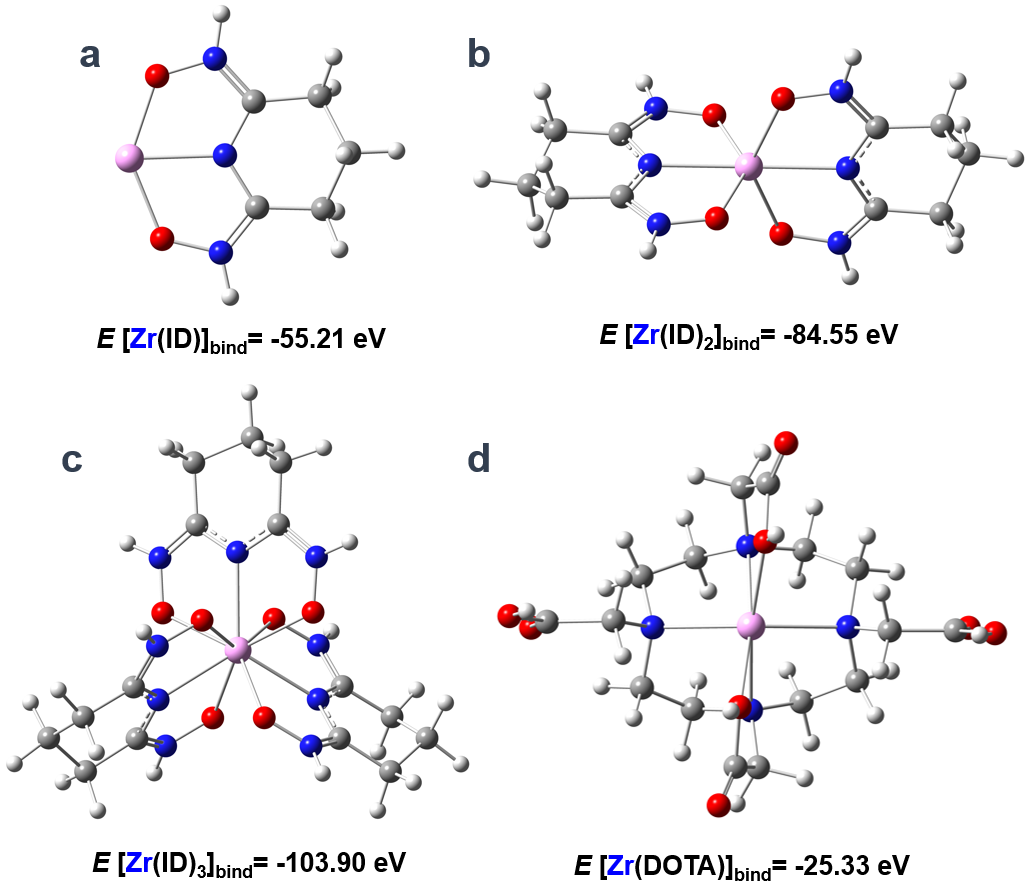


**Fig. S22** Coordination configuration and binding energy of (**a**) [Zr(ID)] complex, (**b**) [Zr(ID)_2_] complex, (**c**) [Zr(ID)_3_] complex, and (**d**) [Zr(DOTA)] complex.


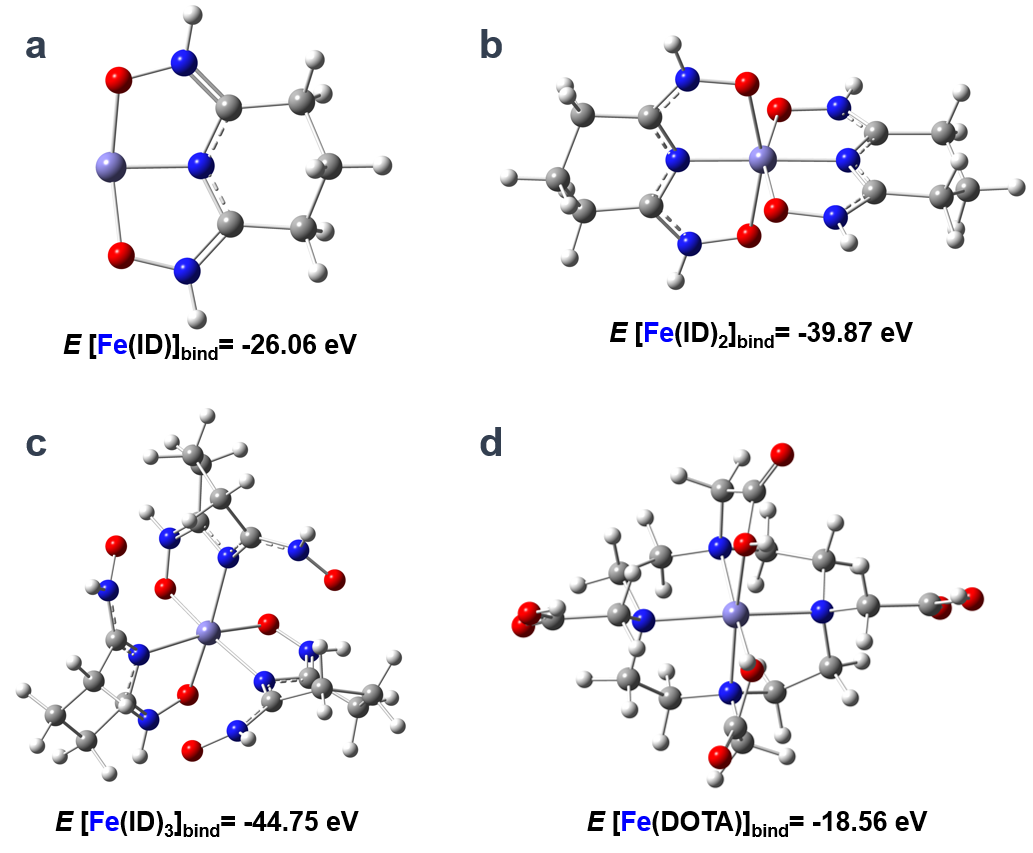
 **Fig. S23** Coordination configuration and binding energy of (**a**) [Fe(ID)] complex, (**b**) [Fe(ID)_2_] complex, (**c**) [Fe(ID)_3_] complex, and (**d**) [Fe(DOTA)] complex.


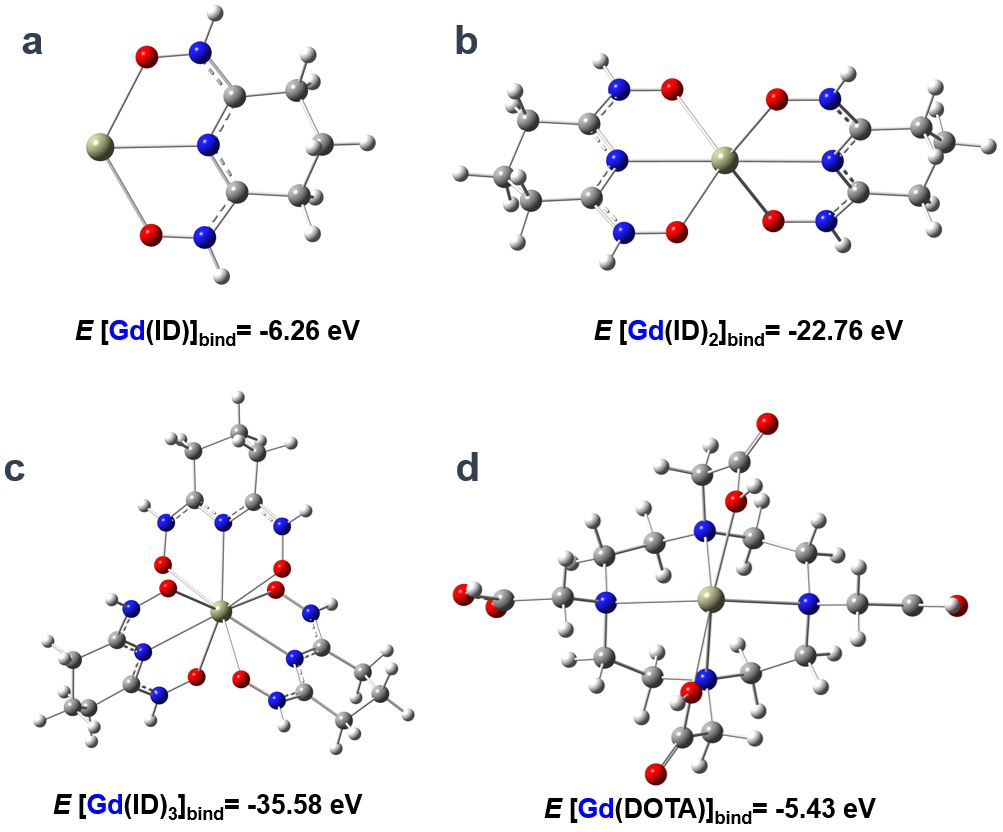


**Fig. S24** Coordination configuration and binding energy of (**a**) [Gd(ID)] complex, (**b**) [Gd(ID)_2_] complex, (**c**) [Gd(ID)_3_] complex, and (**d**) [Gd(DOTA)] complex.


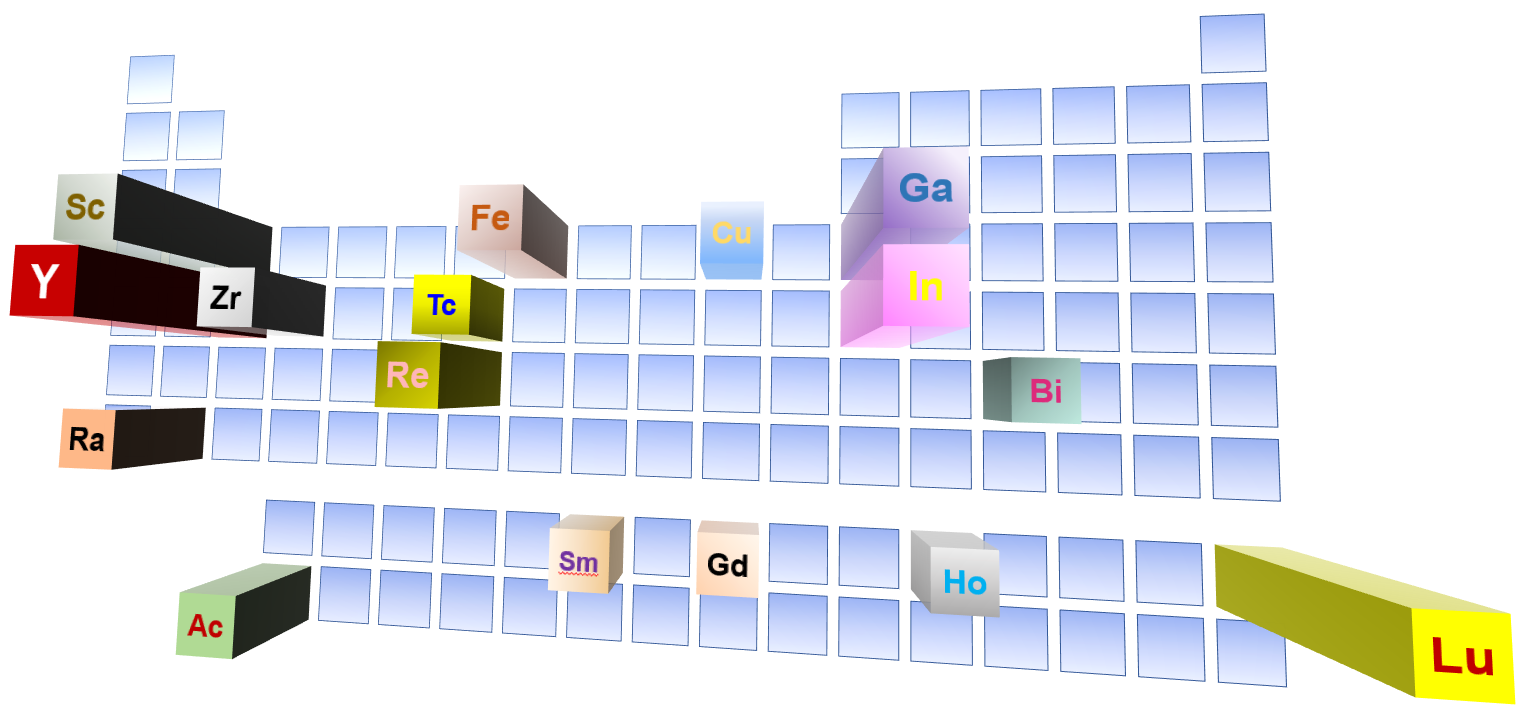


**Fig. S25** The DFT calculation in this work involves a map of the distribution of the positions of all the metallic elements in the periodic table.


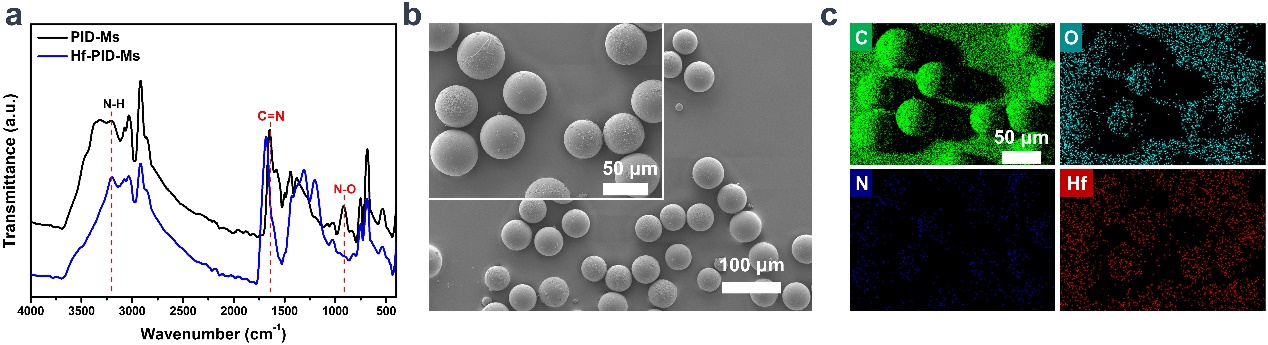


**Fig. S26 a** FT-IR spectra of PID-Ms and Hf-PID-Ms. **b** SEM images of Hf-PID-Ms at different magnifications. **c** EDS mapping of Hf-PID-Ms.


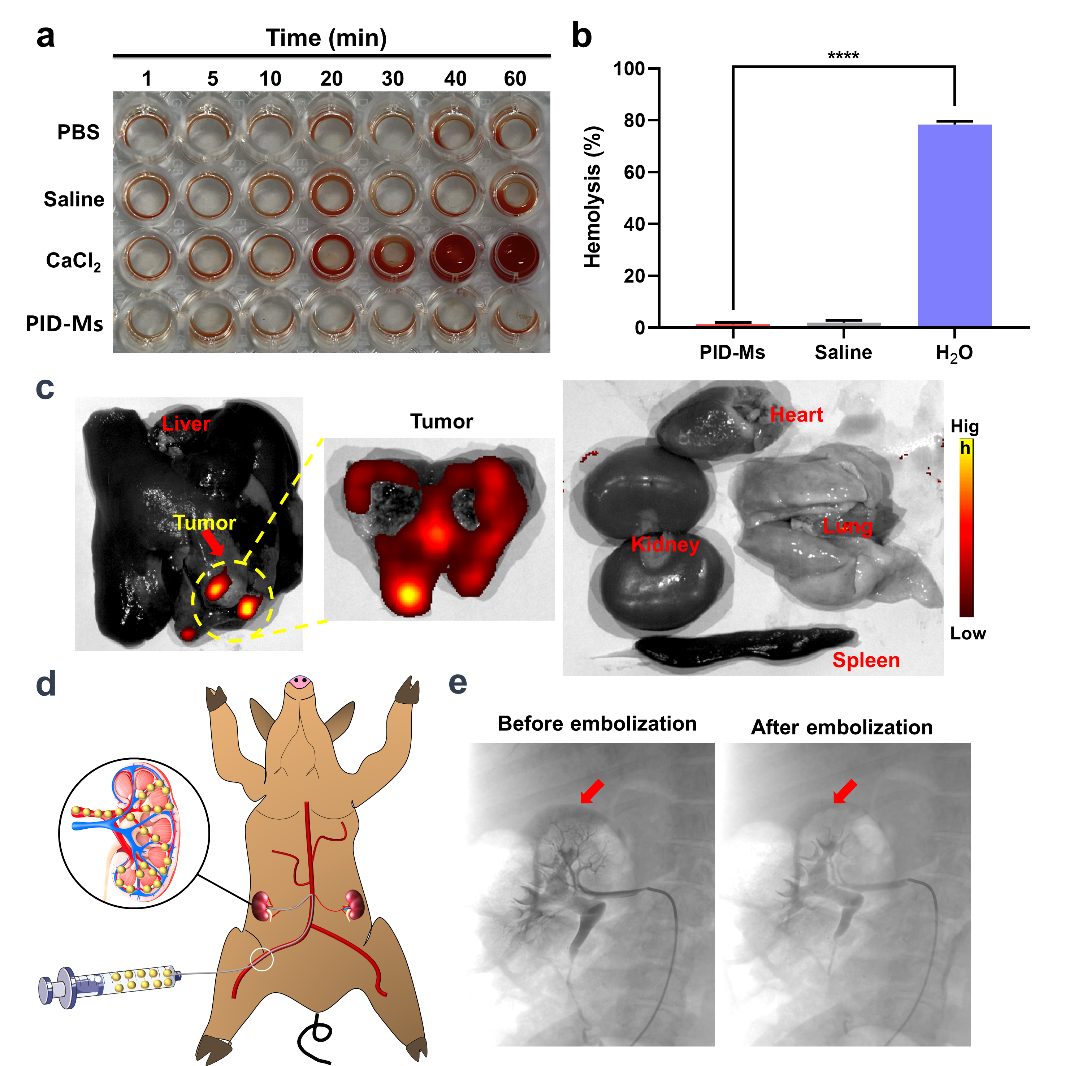


**Fig. S27** ***In vitro* and *In vivo* biocompatibility evaluation of PID-Ms. a** Human blood coagulation test for PID-Ms. **b** Human blood hemolysis test for PID-Ms. **c** *In vivo* imaging system (IVIS) imaging was performed to monitor the biodistribution of PID-Ms on VX2 liver tumor-bearing rabbits. The IVIS images of excised rabbit VX2 liver tumor, Liver, Heart, spleen, lung, and kidney after DSA-guided embolization of Cy 5.5-labeled PID-Ms (10 mg/mL, 0.5 mL) for 5 days. **d** Schematic demonstrating embolization in the pig model of PID-Ms. **e** DSA images of the pig's right kidney before and post-embolization. All the data are presented as mean ± SD (n = 3). Statistical significances were calculated via Welch’s t-test or Mann−Whitney U test. **p* < 0.05, ***p* < 0.01, and ****p* < 0.001.


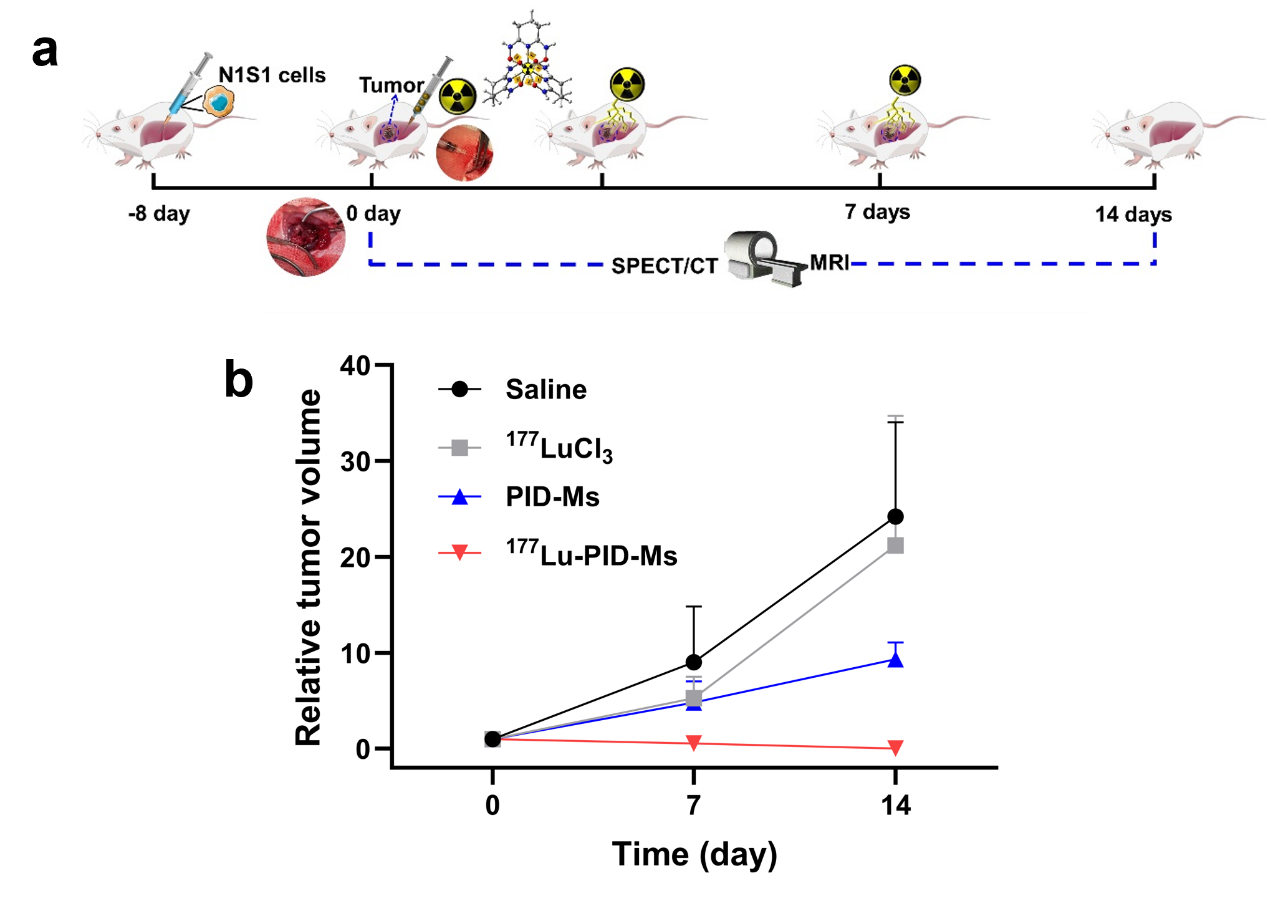


**Fig. S28** Schematic illustration shows the *in vivo* radioembolization procedure of rat N1S1 orthotopic live tumor model.


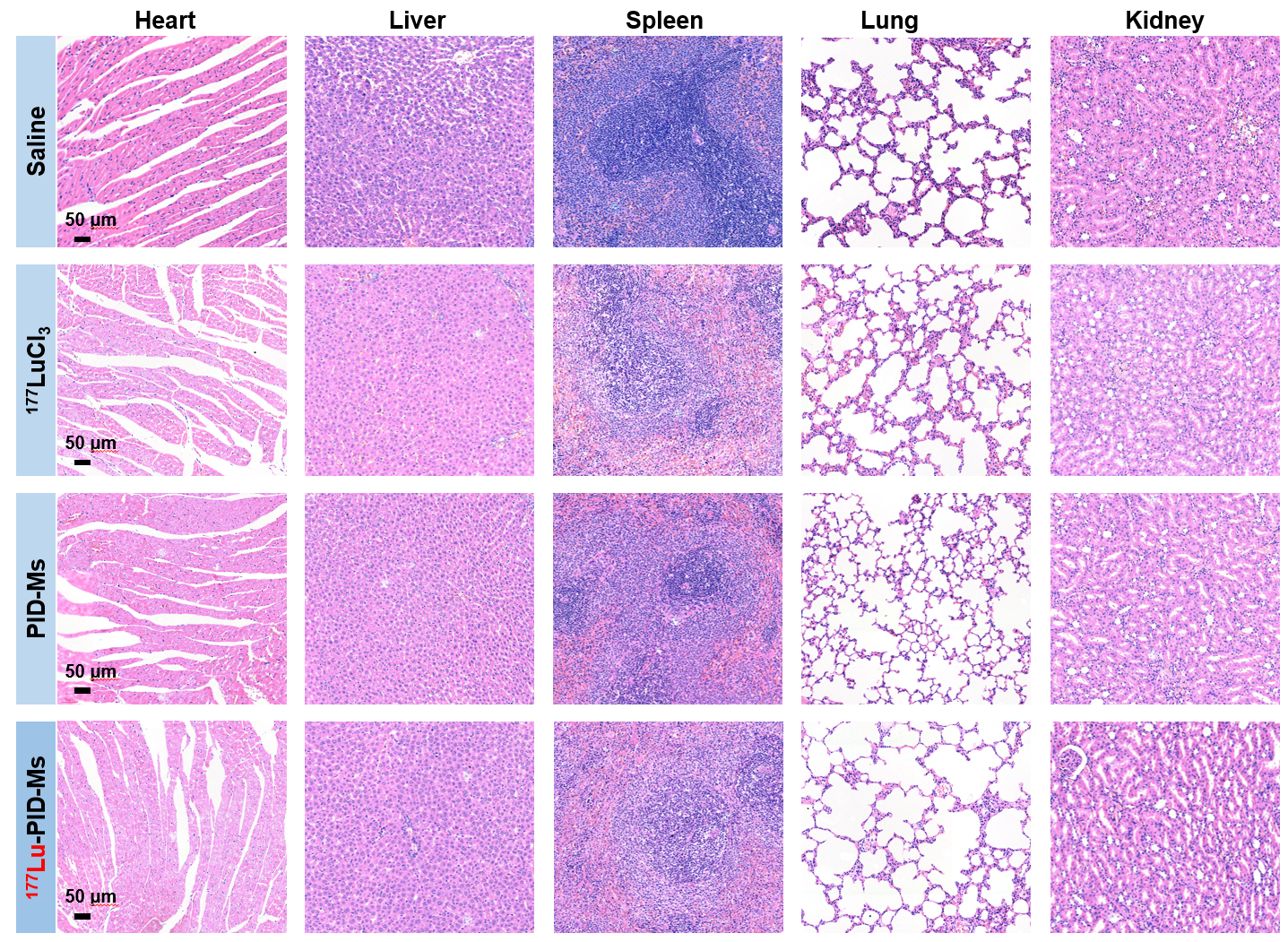


**Fig. S29** H&E staining of the major organs in rats N1S1 liver tumor model after therapy with saline, free ^177^LuCl_3_, PID-Ms, and ^177^Lu-PID-Ms, respectively, at 10 days.


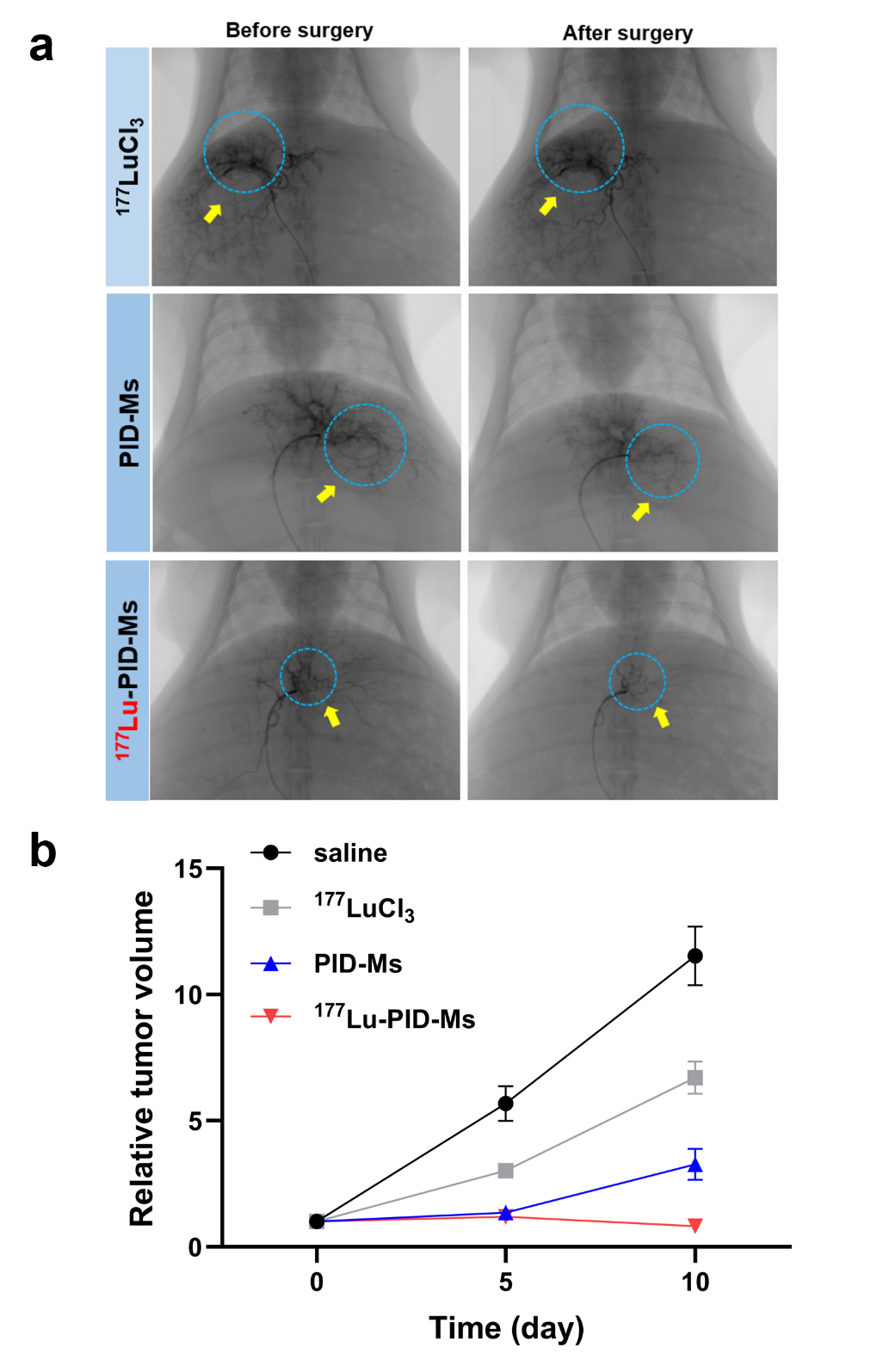


**Fig. S30 a** DSA images of the orthotopic liver VX2 tumor-bearing rabbit before and post-surgery of ^177^LuCl_3_, PID-Ms, and ^177^Lu-PID-Ms. Iohexol was used as a contrast agent. **b** MRI monitoring of relative tumor volume growth curve during 10 days of treatment in the orthotopic model of rabbit VX2 liver cancer.


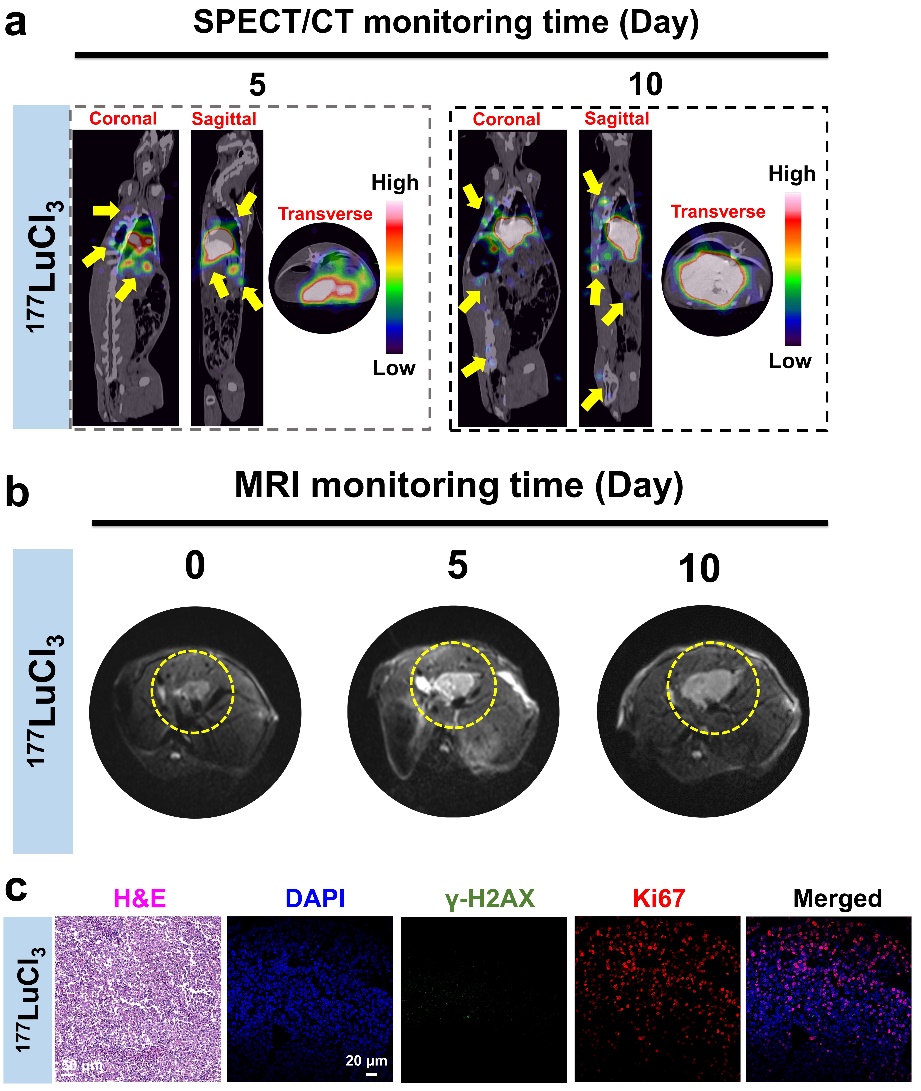


**Fig. S31 a** Representative SPECT/CT images of orthotopic liver VX2 tumor-bearing rabbit at 5 and 10 days after DSA-guided precision delivery of ^177^LuCl_3_ for tumor TARE therapy. **b** Representative MRI imaging of orthotopic liver VX2 tumor-bearing rabbit after therapy with the free ^177^LuCl_3_ for 0, 5, and 10 days. **c** H&E, Ki67 and γ-H2AX staining of representative resected tumor lesions in orthotopic liver VX2 tumor-bearing rabbit model after therapy with free ^177^LuCl_3_, at 10 days.


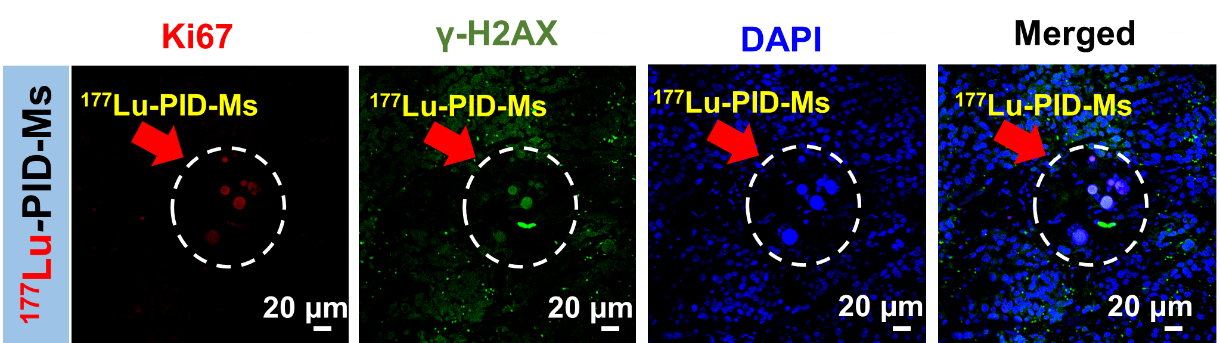


**Fig. S32** Ki67 and γ-H2AX staining of representative resected tumor lesions in orthotopic liver VX2 tumor-bearing rabbit model after therapy with ^177^Lu-PID-Ms, respectively, at 10 days.


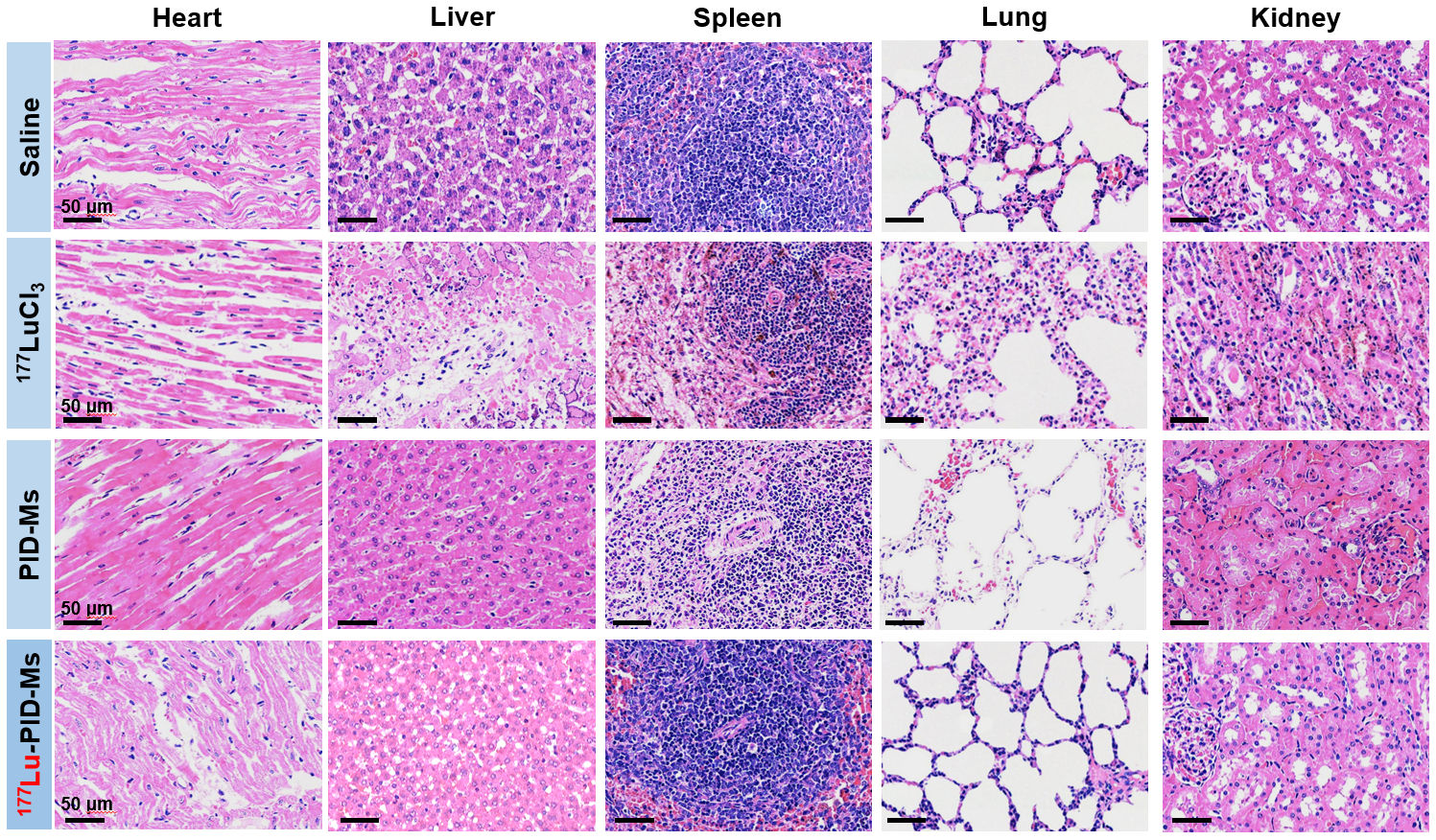


**Fig. S33** H&E staining of the major organs tissue in each group 10 days after TARE with saline, ^177^LuCl_3_, PID-Ms, and ^177^Lu-PID-Ms, respectively.

**Table S1. XPS elemental analyses of the PS, PID-Ms, and Lu-PID-Ms.**

| Sample code | Atomic concentration (%) | | | |
| --- | --- | --- | --- | --- |
|  | C | N | O | Lu |
| **PS** | **100** | **-** | **-** | **-** |
| **PS-*g*-PAN** | 81.57 | 2.39 | 16.04 | - |
| **PID-Ms** | 77.72 | 6.36 | 15.92 | - |
| **Lu-PID-Ms** | 71.75 | 5.74 | 20.91 | 1.6 |

**Table S2. Fitting results for EXAFS of Lu bound PID ligand in the PID-Ms.**

| **Scattering Path** | **CN** | **R(Å)** | **error** | σ^2^ **(Å^2^)** | **ΔE_0_/eV** | **amp** | **R-Factor** |
| --- | --- | --- | --- | --- | --- | --- | --- |
| Lu→O_1_ | 3±0.9 | 2.32 | -0.04 | 0.005 | 2.0±1.6 | 0.95 | 0.01 |
| Lu→O_2_ | 3±0.9 | 2.36 | -0.04 | 0.005 |  |  |  |
| Lu→N_1_ | 3±0.9 | 2.52 | 0.04 | 0.006 | 14.5±0.1 |  |  |
| Lu→N_2_ | 3±0.9 | 3.50 | 0.14 | 0.02 |  |  |  |
| Lu→N_3_ | 3±0.9 | 3.57 | 0.14 | 0.02 |  |  |  |
| Lu→C | 6±1.8 | 3.31 | -0.15 | 0.006 | 2.0±1.6 |  |  |

**Table S3. Comparison of the proposed PID-Ms with commercial TARE microspheres**

| **Feature** | **This work:^177^Lu-PID-Ms** | **Commercial ^90^Y glass microspheres (TheraSphere™)** | **Commercial ^90^Y resin microspheres (SIR‑Spheres®)** | **Commercial ^166^Ho PLA microspheres (QuiremSpheres®)** |
| --- | --- | --- | --- | --- |
| **Matrix material** | Poly(imide dioxime) (PID) conjugated polymer | Non‑degradable glass | ^90^Y‑loaded cation exchange resin | Polylactic acid (degradable) |
| **Size (µm)** | ~40 (monodisperse) | 20–30 | 20–60 (broad) | 15–50 (broad) |
| **Density (g/mL)** | 1.15 (close to blood) | 2.2–2.5 | 1.6 | 1.4 |
| **Radionuclide** | ^177^Lu, ^90^Y, ^188^Re, ^68^Ga, ^99m^Tc, and others | Only ^90^Y | Only ^90^Y | Only ^166^Ho |
| **Reactor dependence** | No high‑flux reactor needed; compatible with generators or low‑flux reactors | Requires high‑flux reactor | Requires high‑flux reactor | Requires high‑flux reactor |
| **Labeling conditions** | 40 °C, 20 min, pH 7, no high temperature | Nuclide embedded during production | Ion‑exchange loading during production | Nuclide embedded during production |
| **Clinical labeling flexibility** | On‑demand (hospital pharmacy, ready‑to‑use) | No (fixed at production) | No (fixed at production) | No (fixed at production) |
| **Shelf life** | Adjustable (dry state long‑term stable, labeled before use) | Short (radionuclide decay) | Short (radionuclide decay) | Short (radionuclide decay) |
| **In vivo stability** | Ultra‑high (<0.01% free nuclide at 240 h) | High (glass non‑degradable, but possible nuclide leaching) | Moderate (resin may degrade) | High (but nuclide release upon polymer degradation) |
| **SPECT/PET imaging capability** | Yes (^177^Lu, ^99m^Tc, ^68^Ga direct imaging) | Bremsstrahlung SPECT only (poor image quality) | Bremsstrahlung SPECT only | Yes (^166^Ho γ‑emission enables SPECT) |
| **Theranostic capability** | Yes (flexible combination of therapeutic and diagnostic nuclides) | No | No | Limited (mainly therapeutic) |
| **Published antitumor data** | Rat & rabbit VX2 orthotopic liver models, T/N ratio >190 | Extensive clinical data | Extensive clinical data | Preclinical & limited clinical data |

**Table S4. Comparison of the radiolabeling strategy used in this work with existing representative chelation systems**

| **Feature** | **This work: PID‑Ms** | **Macrocyclic chelator (DOTA / NOTA)** | **Acyclic chelator (DTPA)** | **Novel macrocyclic/acyclic (macropa, H_4_octapa, etc.)** |
| --- | --- | --- | --- | --- |
| **Ligand type** | Conjugated poly(imide dioxime) ligand | Small‑molecule macrocycle | Small‑molecule acyclic | Small‑molecule (macrocyclic or acyclic, rationally designed) |
| **Applicable radiometals** | Broad (Lu, Y, Re, Ga, Tc, Ho, Ac, Bi, Ra, Sc, Cu, In, Zr, Fe, Gd, etc.) | Broad, but requires ionic radius matching | Broad, but low stability | Optimized for specific metals (e.g., Ac, Pb, Bi) |
| **Labeling temperature** | 40 °C | Typically 80–95 °C | Room temperature or 37°C | Room temperature to 60°C (ligand‑dependent) |
| **Labeling time** | 20 min ( >99 %) | 30–60 min (at high temperature) | 5–30 min | 10–30 min |
| **Labeling pH** | 6–7 (mild) | 4–6 (buffer required) | 5–7 | 5–8 |
| **In vitro stability (physiological)** | Ultra‑high kinetic/thermodynamic inertness (<0.01% free nuclide at 240 h) | High (but some nuclides like ^90^Y may leach) | Low (prone to metal ion exchange) | Moderate to high (ligand‑dependent) |
| **In vivo stability (animal models)** | Excellent (T/N ratio >190, no accumulation in normal organs) | Good (but some nuclide release in bone/liver reported) | Poor (gradually abandoned clinically) | Good (limited long‑term data) |
| **Compatibility with heat‑sensitive targeting vectors** | Compatible (low‑temperature labeling) | Not compatible (high temperature required unless pre‑conjugated) | Compatible | Partially compatible (low‑temperature designs available) |
| **Tunability** | High (polymer architecture can be designed: small molecule, block copolymer, nanoparticle, etc.) | Limited (only side‑chain modifications) | Limited | High (adjustable via side chains) |
| **Ability to form “covalent conformational interlocked network”** | Yes (radiometal‑induced 3D crosslinking) | No | No | No (usually single‑site coordination) |

**Table S5. Detailed radiolabeling conditions of PID-Ms with five radionuclides**

| Parameter | ^177^Lu-PID-Ms | ^90^Y-PID-Ms | ^68^Ga-PID-Ms | ^99m^Tc-PID-Ms | ^188^Re-PID-Ms |
| --- | --- | --- | --- | --- | --- |
| Radionuclide properties | | | | | |
| Half-life | 6.65 days | 64.1 h | 68 min | 6.01 h | 17.0 h |
| Decay mode | β⁻ / γ | β⁻ (pure) | β⁺ / EC | γ / IT | β⁻ / γ |
| Primary energy | 497 keV (β), 208/113 keV (γ) | 2.28 MeV (β) | 1.92 MeV (β⁺) | 140 keV (γ) | 2.12 MeV (β), 155 keV (γ) |
| Labeling conditions (PID-Ms) | | | | | |
| Ligand amount | 10 mg | 10 mg | 10 mg | 10 mg | 10 mg |
| Radionuclide form | ^177^LuCl_3_ (0.04 M HCl) | ^90^YCl_3_ (0.04 M HCl) | ^68^GaCl_3_ (0.1 M HCl) | Na^99m^TcO_4_ (saline) | Na^188^ReO_4_ (saline) |
| Radioactivity per sample | 37 MBq | 18.5 MBq | 37 MBq | 37 MBq | 37 MBq |
| Reducing agent | — | — | — | SnCl_2_ (1 mg/mL, 0.1 M HCl) | SnCl_2_ (1 mg/mL, 0.1 M HCl) |
| Elution/washing | | | | | |
| Neutralization | 0.04 M NaOH | 0.04 M NaOH | — | — | — |
| Washing solution | Saline | Saline | Saline | Saline | Saline |
| Co-ligand/buffer | | | | | |
| Buffer | Deionized water | Acetate buffer | Deionized water | Deionized water | Deionized water |
| Final pH | 7.0 | 7.0 | 7.0 | 7.0 | 7.0 |
| Reaction parameters | | | | | |
| Temperature | 40 °C | 40 °C | 40 °C | 40 °C | 40 °C |
| Reaction time | 20 min | 20 min | 20 min | 20 min | 20 min |
| Performance | | | | | |
| Labeling efficiency | 99.99% | 99.70% | 99.99% | 99.98% | 99.99% |
| In vitro stability (saline) | <0.01% free at 240 h (37 °C) | <0.01% free at 168 h (37 °C) | N.D. | N.D. | N.D. |
| In vitro stability (serum) | <0.01% free at 240 h (37 °C) | N.D. | N.D. | N.D. | N.D. |
| Key references (commercial/standard chelator conditions for comparison) | | | | | |
| DOTA labeling conditions | 80 °C, 20 min, pH 4–4.5 | 80 °C, 20 min, pH 4.5 | 95 °C, 5 min, pH 4.5–4.8 | N/A | 95 °C, 45 min, pH 4–5 |
| NOTA labeling conditions | — | — | RT, 10 min, pH 3.5 or 6.5 | — | — |
| HYNIC labeling conditions | — | — | — | 100 °C, 15 min, pH 3 or 5 | 100 °C, 3 h, pH 2.9 (for DMSA) |
